# Supplementary material for: Temporal dynamics of collateral RNA cleavage by LbuCas13a in human cells
Source: Commun Biol. 2026 Jan 19;9:233. doi: 10.1038/s42003-026-09511-3 (PMC12901986; doi:10.1038/s42003-026-09511-3)
Supplement: Supplementary file 2 — Supplementary Information [file 42003_2026_9511_MOESM2_ESM.pdf]

Temporal dynamics of collateral RNA cleavage by LbuCas13a in human cells

Jorik Frederik Bot[1,2 ‡], Zhihan Zhao[1,2,3 ‡], Mengyuan Li[1,2], Darnell Kammeron[1,2], Peng Shang[1,2, *] and Niels Geijsen[1,2, *]

[1] Dept. of Anatomy & Embryology, Leiden University Medical Center, Einthovenweg 20, 2300 RC, Leiden, The Netherlands.

[2] The Novo Nordisk Foundation Center for Stem Cell Medicine (reNEW), Leiden node, Leiden, The Netherlands

[3] Biomedical Pioneering Innovation Center, Peking-Tsinghua Center for Life Sciences, Peking University Genome Editing Research Center, State Key Laboratory of Gene Function and Modulation Research, School of Life Sciences, Peking University, Beijing 100871, China

‡ These authors contributed equally to this work.

* Leiden University Medical Center, Dept. of Anatomy & Embryology, Building 2, Einthovenweg 20, 2300 RC Leiden, The Netherlands. +31-(0)71-526 9302, p.shang@lumc.nl and n.geijsen@lumc.nl

a

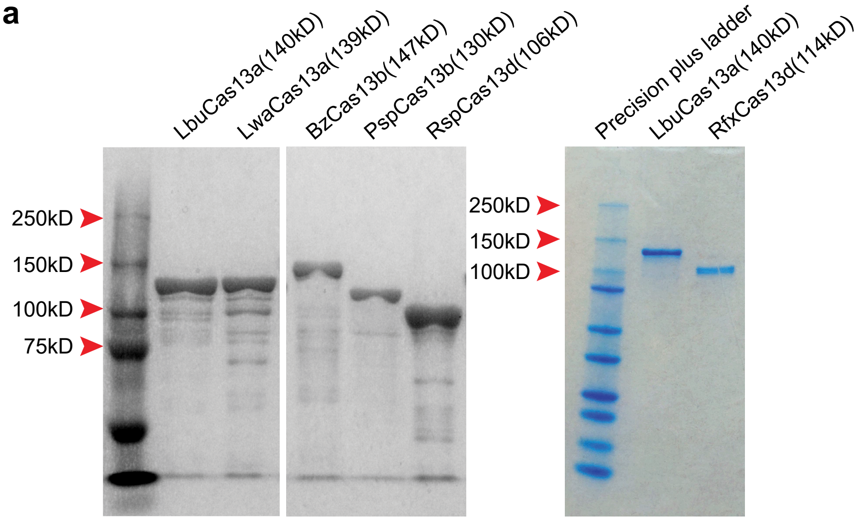

b

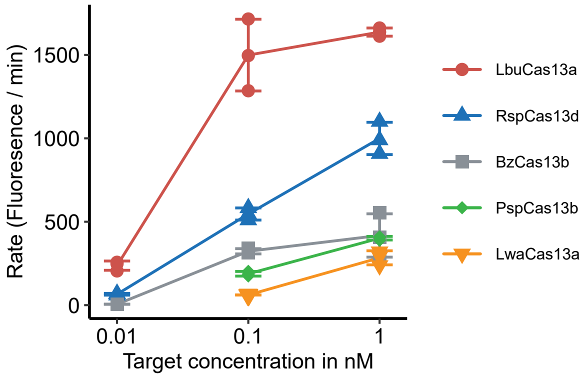

c

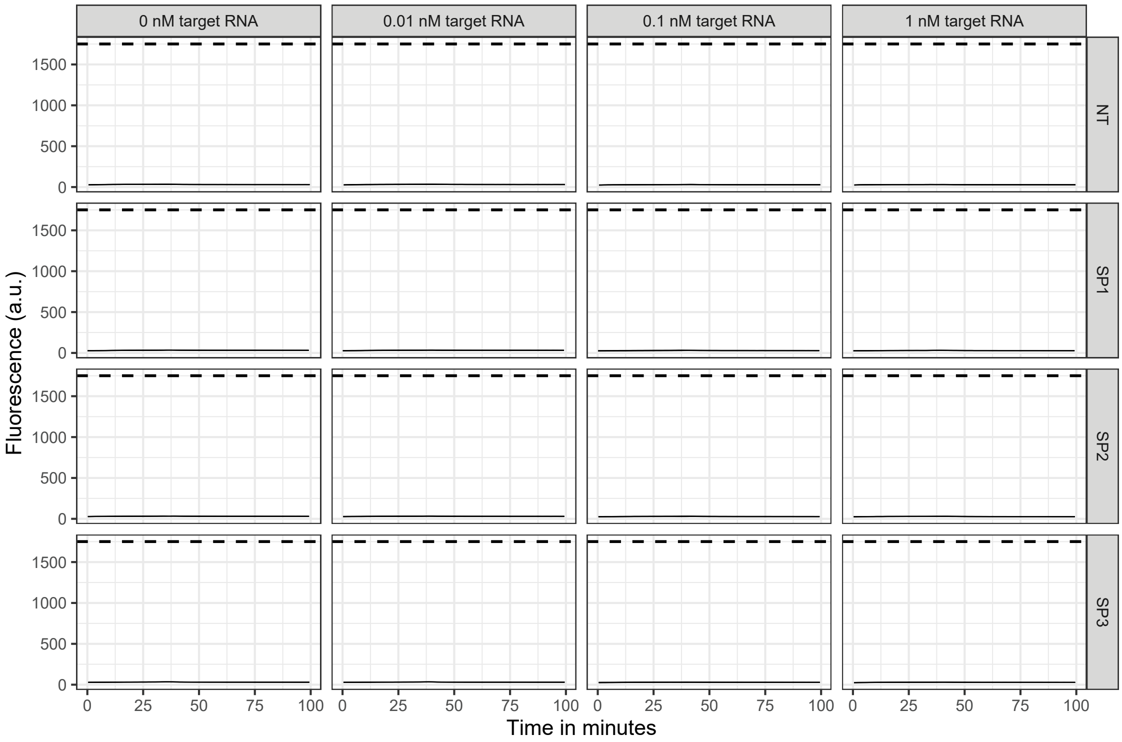

J. F. Bot et al. Supplementary Figure 1

Supplementary Figure 1. SDS-PAGE of recombinant proteins and RNaseAlert assays. **a** SDS-PAGE gels of Cas13 orthologs after HIS-tag purification followed by fast protein liquid chromatography (FPLC) gel filtration. All proteins have the expected size. **b** IDT RNaseAlert assay (n=3). Cas13 proteins were incubated with a *dEGFP* targeting guide RNA and three different concentrations of *dEGFP* target RNA. The cleavage rate was calculated by taking the slope of the initial linear increase in fluorescence intensity. Error bars show standard deviation. **c** RfxCas13d does not show any activity in the RNaseAlert assay (n=3). RfxCas13d was incubated with either a non-targeting (NT) or one of three different *dEGFP* targeting guide RNAs (SP1, SP2, SP3), and 0, 0.01, 0.1 or 1 nM dEGFP target RNA. Fluorescence was measured every minute. No increase in fluorescence was observed. The dashed horizontal line shows the maximum fluorescence of the RNase A positive control.

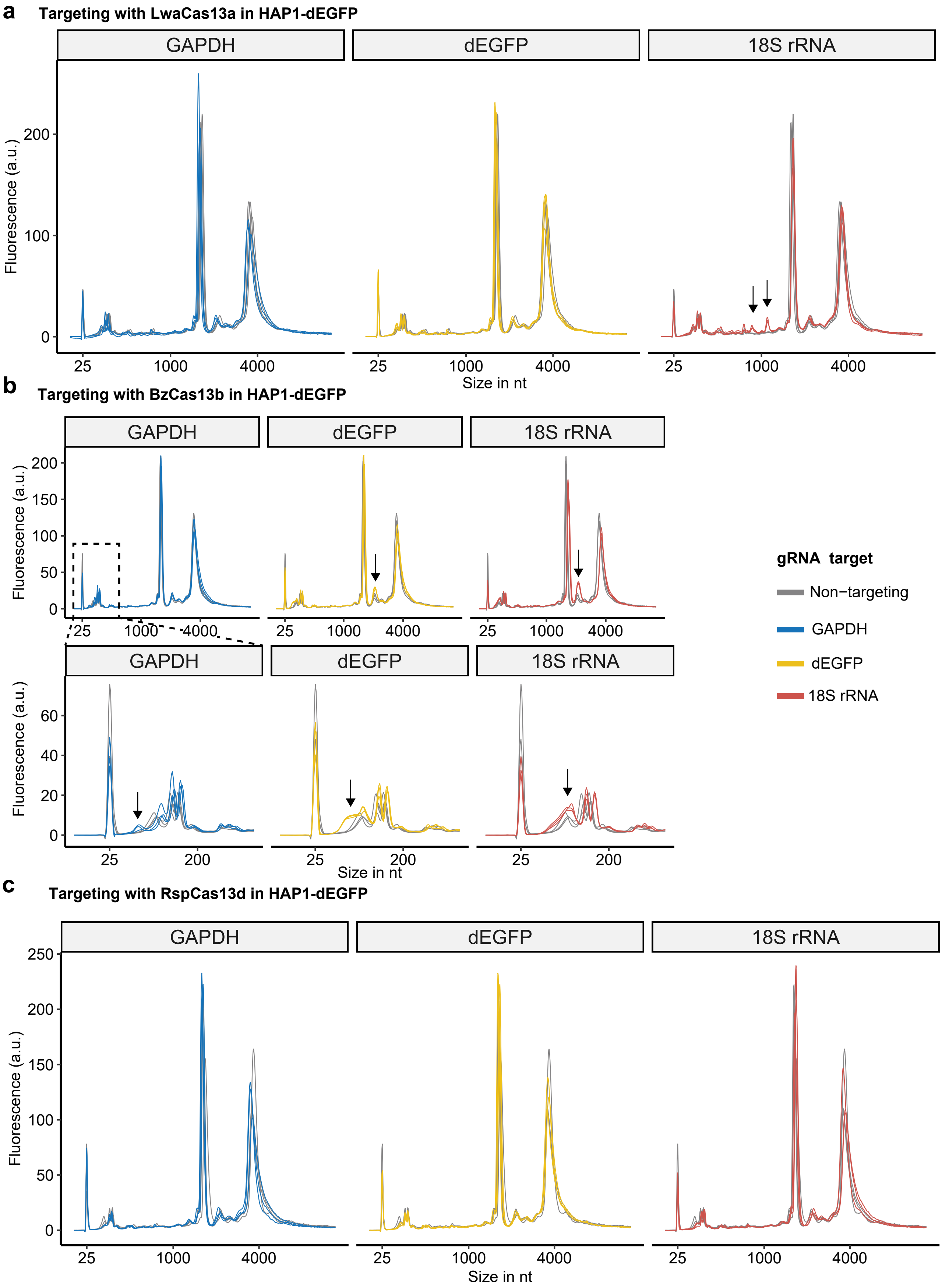

J. F. Bot et al. Supplementary Figure 2

Supplementary Figure 2. Comparing the collateral activity in cells of different Cas13 orthologs. **a** Total RNA profiles after *GAPDH*, *dEGFP* and *18S rRNA* targeting with LwaCas13a in HAP1-dEGFP (n=3). **b** Total RNA profiles after *GAPDH*, *dEGFP* and *18S rRNA* targeting with BzCas13b in HAP1-dEGFP (n=3). **c** Total RNA profiles after *GAPDH*, *dEGFP* and *18S rRNA* targeting with RspCas13d in HAP1-dEGFP (n=3). For all plots, RNA isolated 100 minutes after transfection using iTOP. Fluorescence was adjusted to the total area under the curve, to correct for differences in loaded amount of RNA.

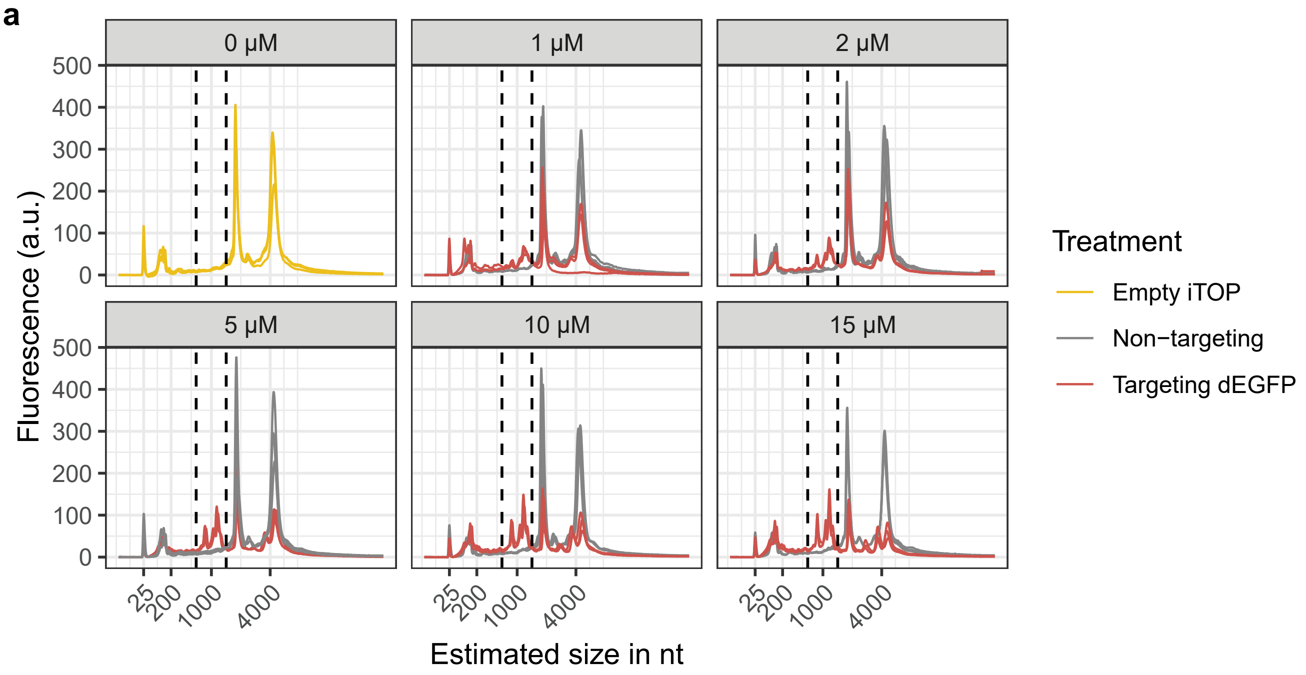
a
0 µM
1 µM
2 µM
5 µM
10 µM
15 µM
Fluorescence (a.u.)
500
400
300
200
100
0
25
200
1000
4000
Estimated size in nt
Treatment
Empty iTOP
Non−targeting
Targeting dEGFP

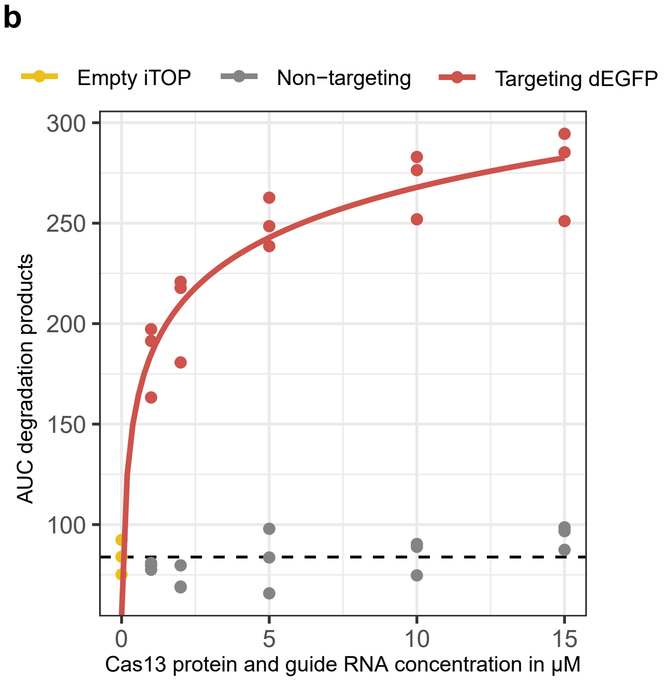
b
Empty iTOP
Non−targeting
Targeting dEGFP
AUC degradation products
300
250
200
150
100
0
5
10
15
Cas13 protein and guide RNA concentration in µM

Supplementary Figure 3. Total RNA degradation by LbuCas13a increases with the RNP concentration. a Total RNA profiles 100 minutes after transfection of HAP1-dEGFP with different concentrations of LbuCas13a recombinant protein and guide RNA, as determined by bioanalyzer (n=3). An equimolar ratio of protein to guide was used. Fluorescence intensity was adjusted to the total area under the curve of each sample. **b** The area under the curve of the degradation products (area between the vertical dashed lines in **a**) increases with the protein and guide concentration (n=3). The trendline follows y = log(x).

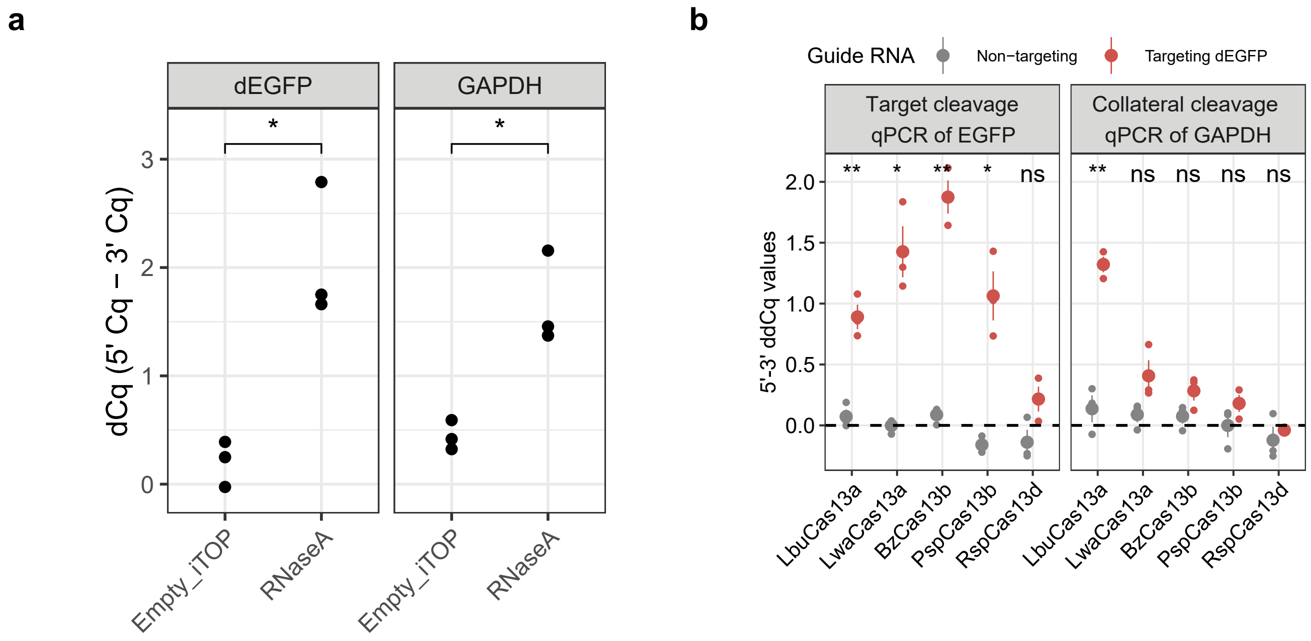

J. F. Bot et al. Supplementary Figure 4

Supplementary Figure 4. Comparing target and collateral RNA cleavage of different Cas13 orthologs using the 5':3' RT-qPCR assay. a 5'-3' RT-qPCR assay (n=3) on HAP1-dEGFP cells transfected with RNase A. RNA was isolated 50 minutes after transfection. P-values calculated by unpaired two-sided Welch's t-test (* < 0.05). RNase A transfections yielded the expected increase in 5' qPCR primer Cq values relative to the 3' qPCR primers. **b** Cleavage of target (*dEGFP*) and collateral (*GAPDH*) RNA as assessed by the 5'-3' RT-qPCR assay (n=3). RNA isolated 50 minutes after transfection of HAP1-dEGFP with Cas13 protein and either a non-targeting or dEGFP targeting guide RNA. Only LbuCas13a showed significant collateral RNA (*GAPDH*) cleavage. P-values calculated by two-sided unpaired Welch's t-test (ns = not significant, * < 0.05, ** < 0.01). Small dots are independent replicates. Big dot and line show mean and standard error of the mean.

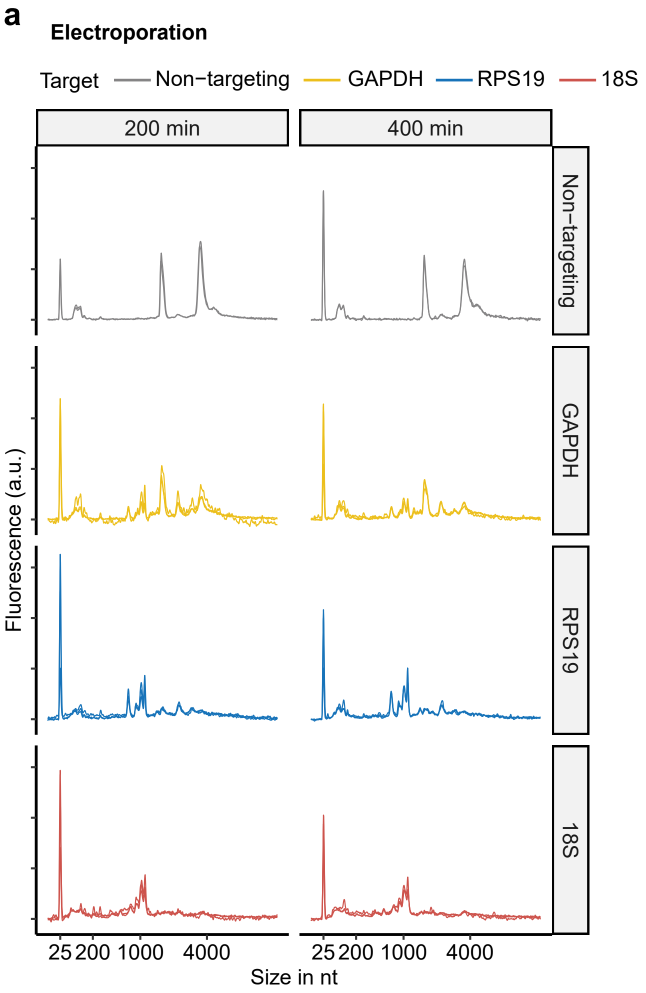

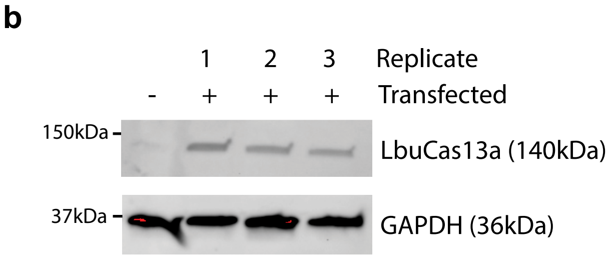

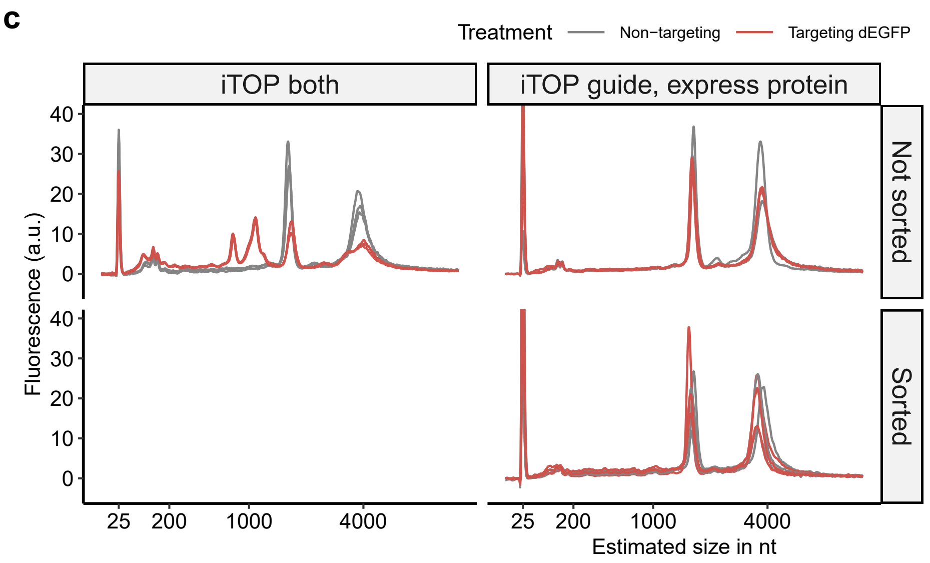

J. F. Bot et al. Supplementary Figure 5

Supplementary Figure 5. RNP delivery by electroporation also results in collateral RNA cleavage. a Total RNA profiles at 200 and 400 minutes after electroporation of HAP1-dEGFP with LbuCas13a and a *GAPDH*, *RPS19*, *18S rRNA* or non-targeting guide RNA (n=3). Fluorescence intensity was adjusted to the total area under the curve of each sample. **b** Western blot after transient transfection of HAP1-dEGFP with an LbuCas13a expression vector. LbuCas13a was HA-tagged and an HA-tag antibody used as primary antibody. Western blot was performed two days after plasmid transfection on unsorted cells with an average transfection efficiency of 9.2%. Three independent transfections were performed. **c** No collateral RNA cleavage was detected on LbuCas13a expressing HAP1-dEGFP cells after transfection with a *dEGFP* targeting guide RNA. Total RNA profiles 100 minutes after iTOP transfection, as determined by bioanalyzer (n=3). Fluorescence intensity was adjusted to the total area under the curve of each sample.

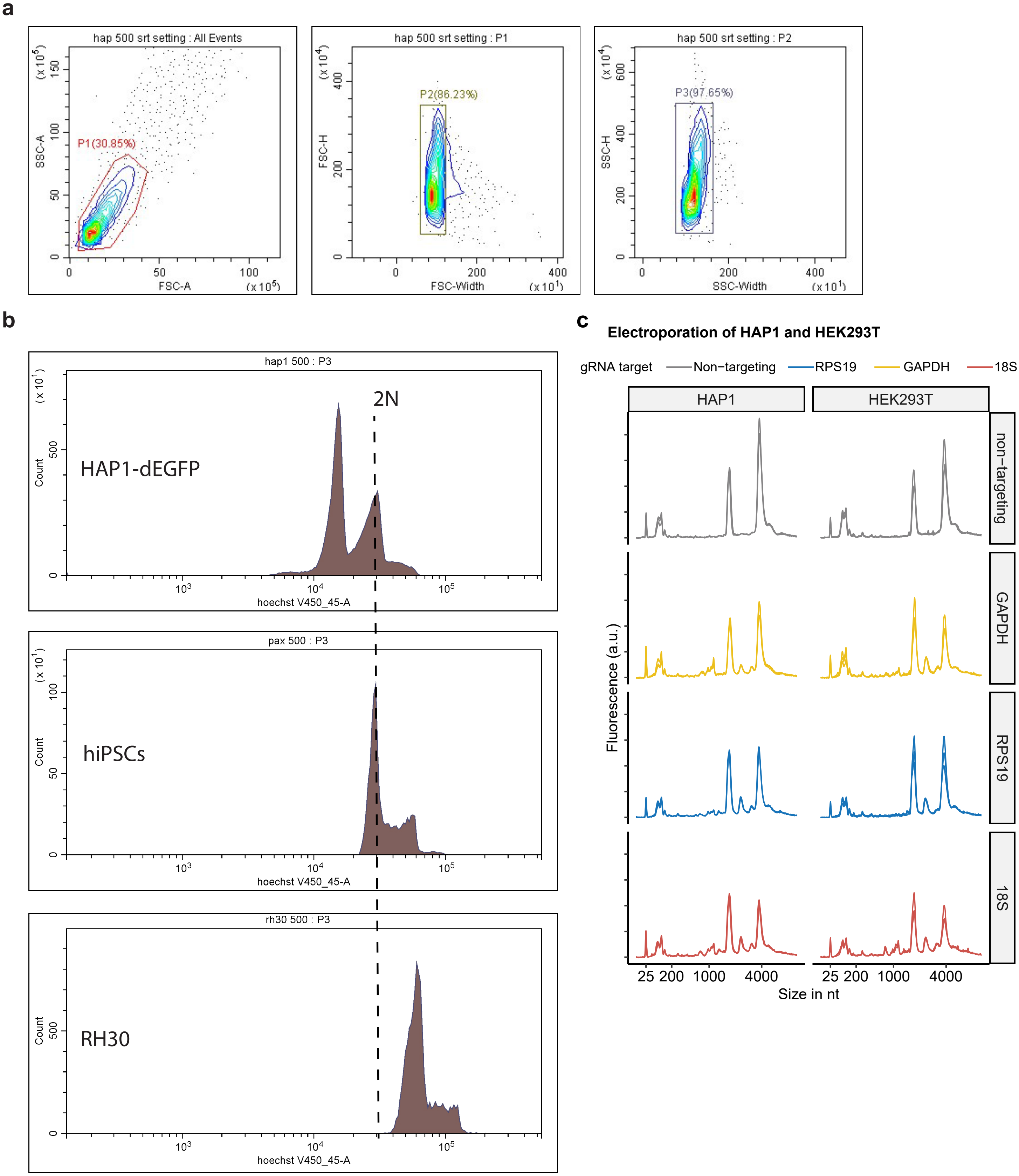

J. F. Bot et al. Supplementary Figure 6

Supplementary Figure 6. HAP1-dEGFP cells are a combination of haploid and diploid cells. a An example of the FACS gating strategy used in **b**. **b** To assess their ploidy, HAP1-dEGFP, hiPSCs and RH30 cells were plated in 6-well plates at 50% confluency. Cells were incubated with 500nM palbociclib for 24 hours to arrest them in the G1 phase. Next they were fixed in 70% ethanol and stained with Hoechst. The Hoechst intensities were compared on the CytoFlex SRT. hiPSCs were from[1]. **c** Total RNA profiles 200 minutes after electroporation of HAP1-dEGFP and HEK293T cells (n=3). Fluorescence intensity was adjusted to the total area under the curve of each sample.

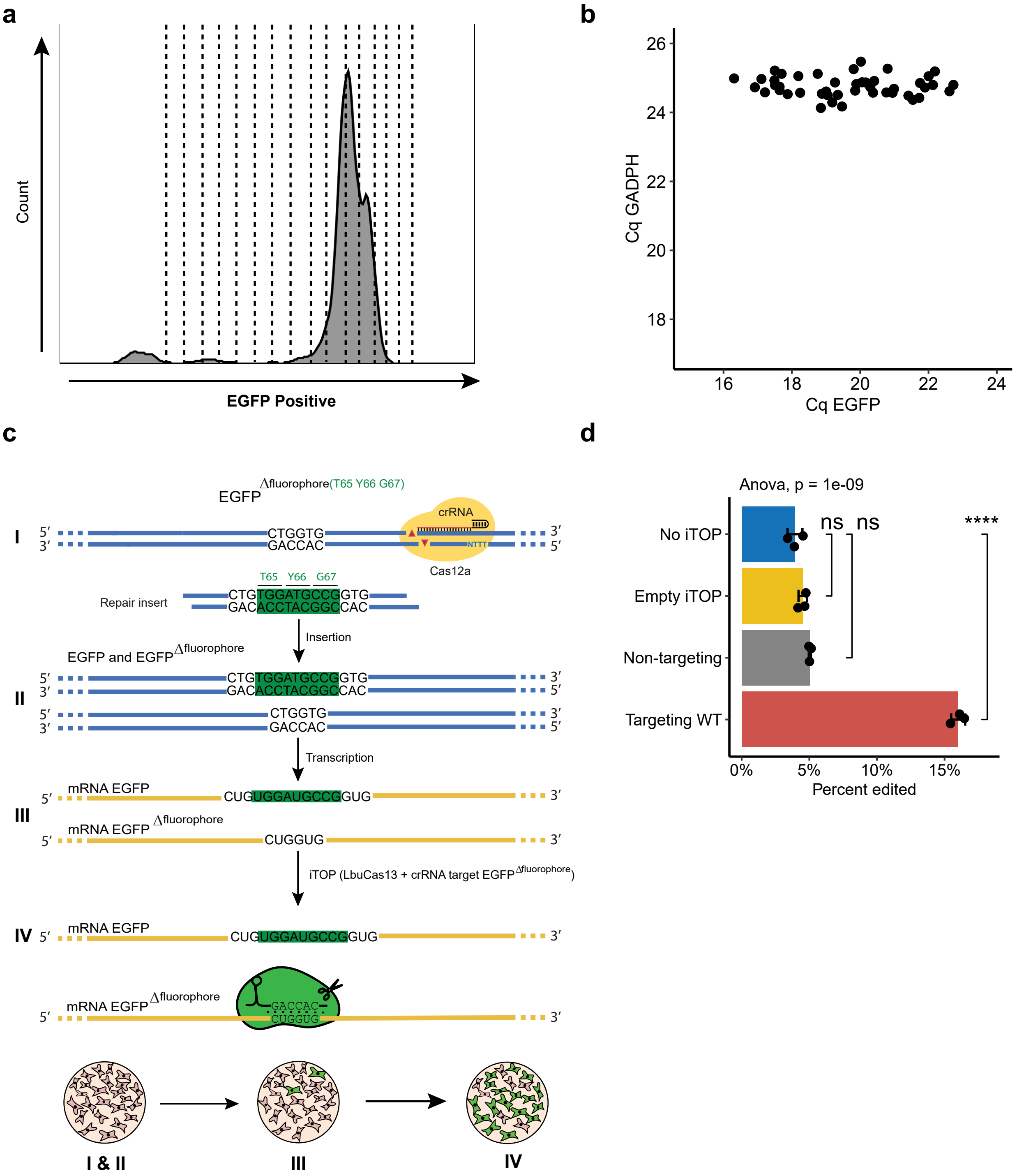

J. F. Bot et al. Supplementary Figure 7

Supplementary Figure 7. Generating lines with various levels of EGFP expression, and Cas12 gene editing strategy and selection. a Gating strategy to sort different EGFP intensities. **b** qPCR on HAP1-EGFP cells gated for different EGFP intensities. A wide range of *EGFP* expression levels were recovered after sorting. **c** Schematic showing the LAHR editing strategy, see Zhao et al. for details[2]. **d** Enrichment of edited cells by targeting unedited cells with LbuCas13a (n=3). The percentage of EGFP positive cells was assess on the BD FACS Canto II two days after selection with LbuCas13a. Error bars show standard deviation. P-values calculated by unpaired Welch's t-test (ns = not significant, **** = P-value < 0.0001).

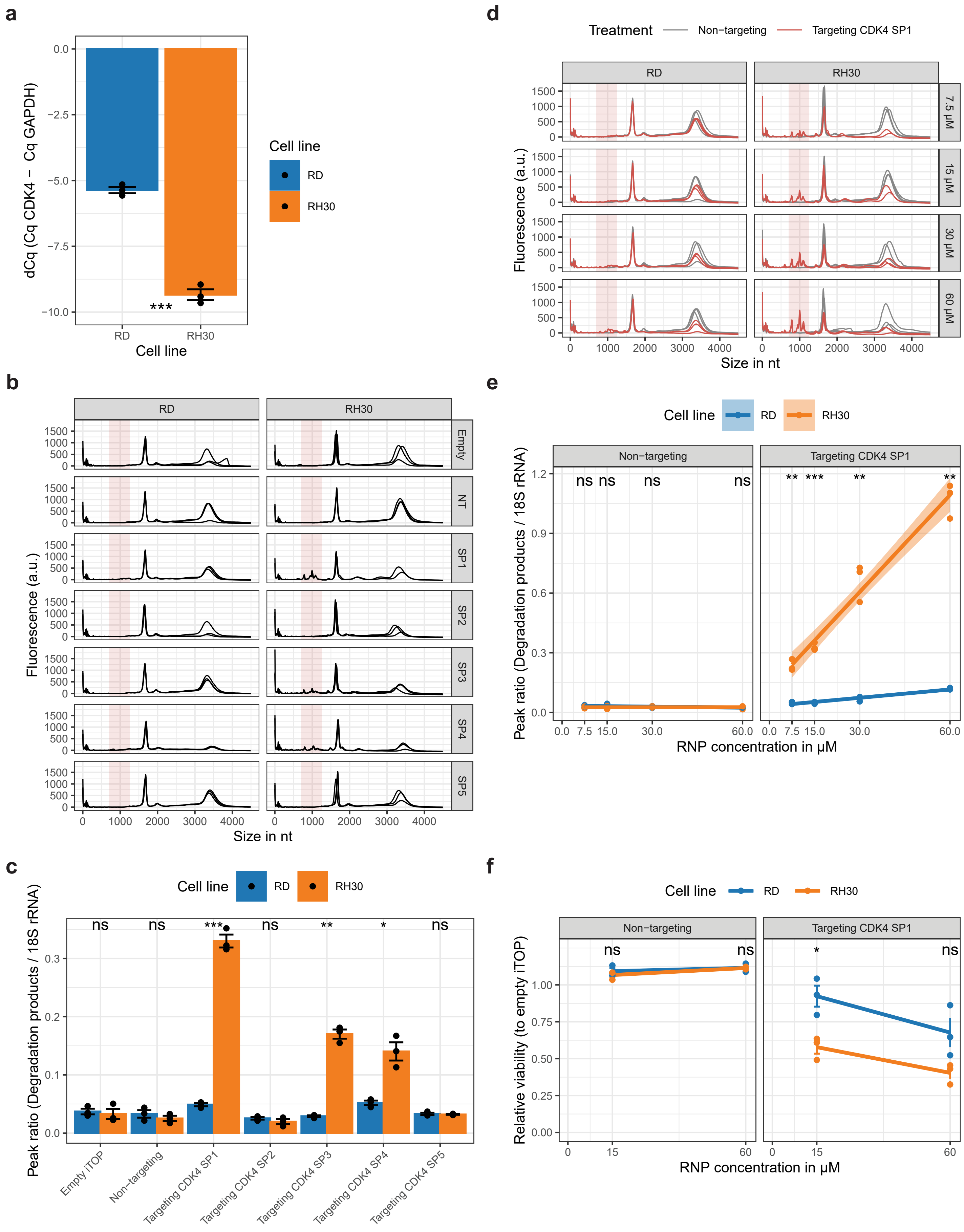

J. F. Bot et al. Supplementary Figure 8

Supplementary Figure 8. Optimization of CDK4 targeting to selectively eliminate RH30 cells. a qPCR confirms overexpression of *CDK4* in RH30 relative to RD (n=3). dCq values are relative to *GAPDH*. Lower dCq values indicate higher *CDK4* expression. Cell lines were compared by two-sided unpaired Welch's t-test (*** = p-value < 0.001) **b** *CDK4* targeting guide RNAs have different levels of collateral activity in RH30, while all showing minimal collateral activity in RD. RD and RH30 cells were transfected with either an empty iTOP (Empty), a transfection with LbuCas13a and a non-targeting guide (NT), or with LbuCas13a and 1 of 5 different *CDK4* targeting guide RNAs (SP1-SP5). Protein and guide concentration were 15 µM for all samples. RNA was collected 100 minutes after iTOP and the total RNA profiles were determined on the Agilent Femto Pulse (n=3 except for RH30 SP1 where n=2 due to a broken column during RNA purification). Light red shaded rectangles indicates the size range between 700 and 1250 nt where most collateral cleavage fragments appear. **c** The *CDK4* SP1 guide RNA shows the highest collateral activity in RH30 and the biggest difference in activity between RH30 and RD. The ratio between the maximum fluorescence intensity of the collateral cleavage fragments between 650 and 1350 nucleotides and the maximum fluorescence intensity of the *18S rRNA* peak (max between 1500 and 2000 nt) is shown (n=3). Cell lines were compared by two-sided unpaired Welch's t-test (ns = not significant, * = p-value < 0.05, ** = p-value < 0.01, *** = p-value < 0.001) **d** The collateral activity after targeting *CDK4* with LbuCas13a and SP1 increases with the protein and guide concentration. RNA was collected 100 minutes after iTOP and the total RNA profiles were determined on the Agilent Femto Pulse (n=3 except for RH30 targeting *CDK4* SP1 7.5 µM and 15 µM where n=2 due to a broken column during RNA purification). Light red shaded rectangles indicates the size range between 700 and 1250 nt where most collateral cleavage fragments appear. **e** The collateral activity of the *CDK4* SP1 targeting guide RNA increases with the protein and guide concentration in both cell lines. RH30 shows a much steeper increase than RD. The ratio between the maximum fluorescence intensity of the collateral cleavage fragments between 650 and 1350 nucleotides and the maximum fluorescence intensity of the 18S rRNA peak (max between 1500 and 2000 nt) is shown (n=3). Cell lines

were compared by two-sided unpaired Welch's t-test (ns = not significant, ** = p-value < 0.01, *** = p-value < 0.001) **f** Reduction in viability after targeting *CDK4* in RD and RH30. Viability was assessed using the CellTiter-Glo assay 2 days after iTOP. Values are shown relative to the empty iTOP control. In all panels, RD and RH30 were compared using an unpaired two-sided Welch's t-test (ns = not significant, * = p-value < 0.05). All error bars and bands show the standard error.

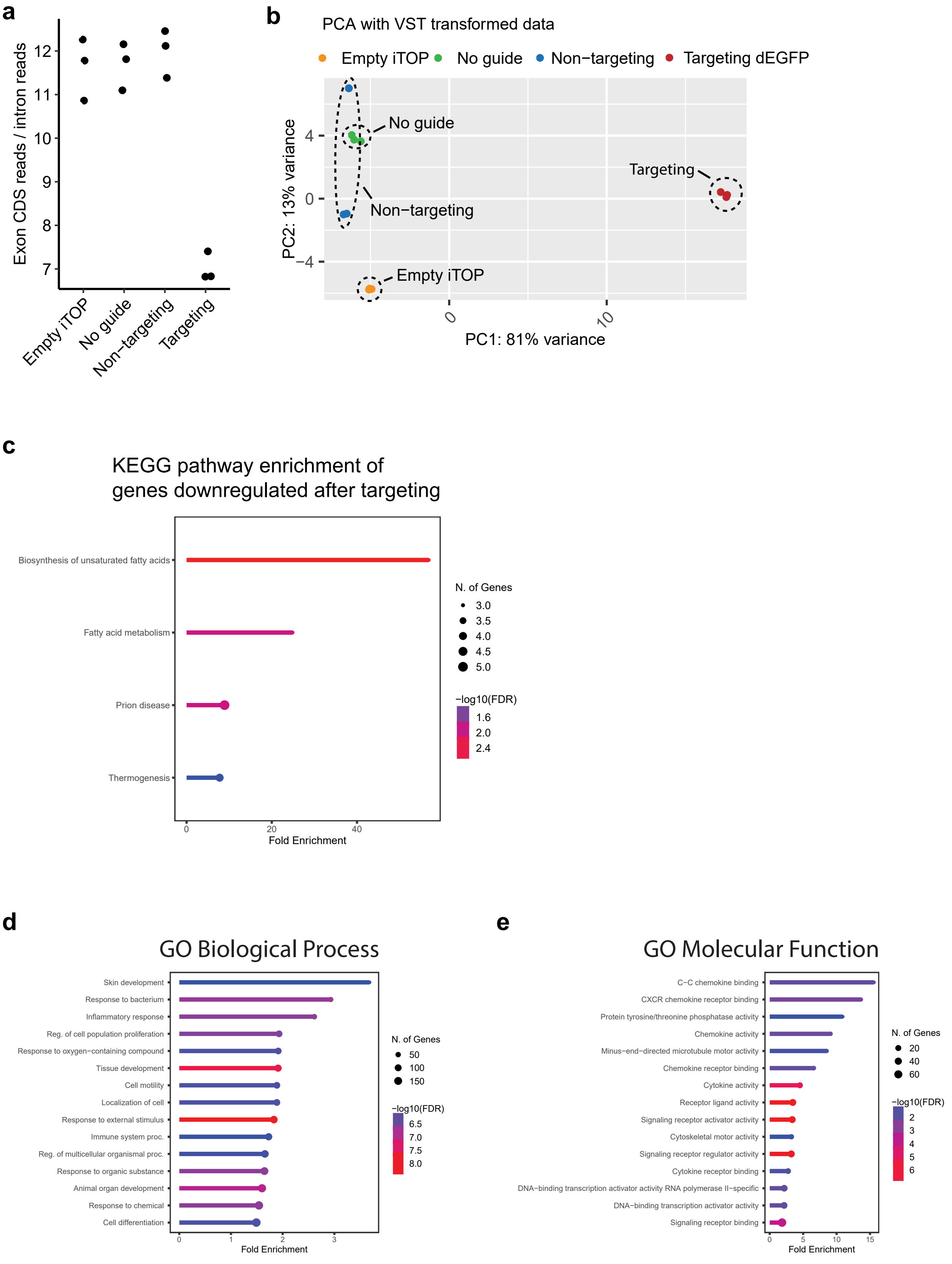

J. F. Bot et al. Supplementary Figure 9

Supplementary Figure 9. RNA-seq data analysis. a Targeting with LbuCas13a increase the abundance reads mapped to introns relative to reads mapped to exons (n=3). **b** PCA plot of the RNA-seq samples using the default setting of the plotPCA function in DESeq2[3]. **c** A ranked list of enriched KEGG pathways in the significantly downregulated genes in response to *dEGFP* targeting with LbuCas13a (adjusted P-value < 0.05 & Log2 FC > 1). Plots generated using ShinyGo[4]. **d** Top enriched GO Biological Process term using the significantly upregulated genes in response to *dEGFP* targeting with LbuCas13a. Plot generated using ShinyGo[4]. **e** Top enriched GO Molecular function term of the significantly upregulated genes in response to *dEGFP* targeting with LbuCas13a. Plot generated using ShinyGo[4].

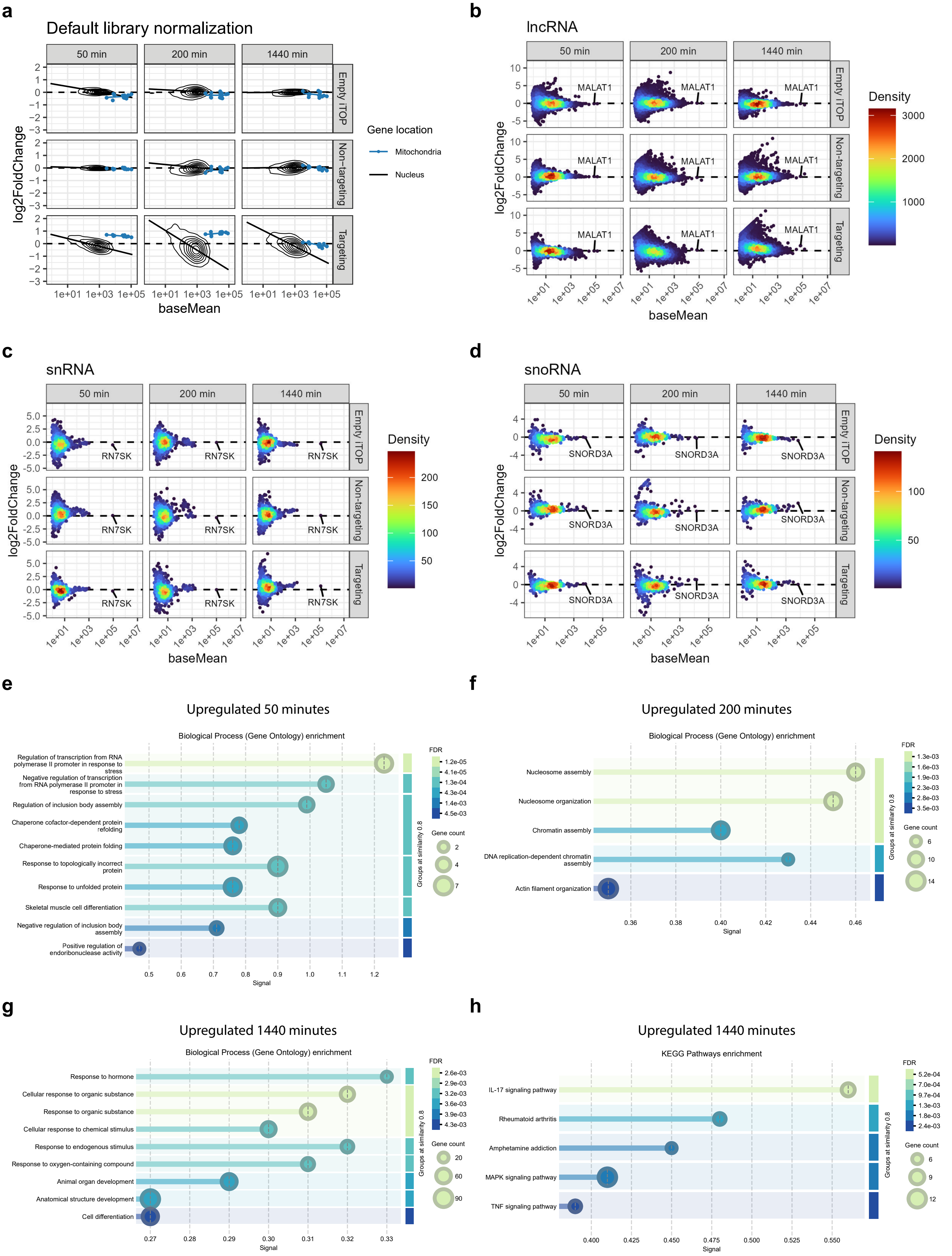

J. F. Bot et al. Supplementary Figure 10

Supplementary Figure 10. Total RNA sequencing with ERCC spike-in RNAs. a Density plots of the log2 Fold Changes versus the baseMean expression for all protein coding transcripts in response to an empty iTOP transfection, transfection with LbuCas13a and a non-targeting guide RNA, or with LbuCas13a and a *dEGFP* targeting gRNA. The default library normalization of DESeq2 was used (see methods). Columns show minutes after iTOP transfection. Experiments performed with HAP1-dEGFP. n=3 except for the empty iTOP condition at 50 minutes after iTOP where n=2 due to one sample failing library prep and sequencing QC. **b**, **c** and **d** Density plots of the log2 Fold Changes versus the baseMean expression for lncRNA, snRNA and snoRNAs transcripts respectively in response to an empty iTOP transfection, transfection with LbuCas13a and a non-targeting guide RNA, or with LbuCas13a and a dEGFP targeting gRNA. Libraries were normalized to ERCC spike-in control RNAs before differential gene expression with DESeq2 (see methods). Columns show minutes after iTOP transfection. Experiments performed with HAP1-dEGFP. n=3 except for the empty iTOP condition at 50 minutes after iTOP where n=2 due to one sample failing library prep and sequencing QC. **e** Enriched GO Biological process terms of genes one Log2 Fold Change above the linear trendline shown in Figure 6a in response to *dEGFP* targeting with LbuCas13a at 50 minutes after transfection. Plot generated on STRING-db.org[5] **f** Enriched GO Biological process terms of genes two Log2 Fold Change above the linear trendline shown in Figure 6a in response to *dEGFP* targeting with LbuCas13a at 200 minutes after transfection. Plot generated on STRING-db.org **g** Enriched GO Biological process terms of genes two Log2 Fold Change above the linear trendline shown in Figure 6a in response to *dEGFP* targeting with LbuCas13a at 1440 minutes after transfection. Plot generated on STRING-db.org **h** Enriched KEGG pathways of genes two Log2 Fold Change above the linear trendline shown in Figure 6a in response to *dEGFP* targeting with LbuCas13a at 1440 minutes after transfection. Plot generated on STRING-db.org

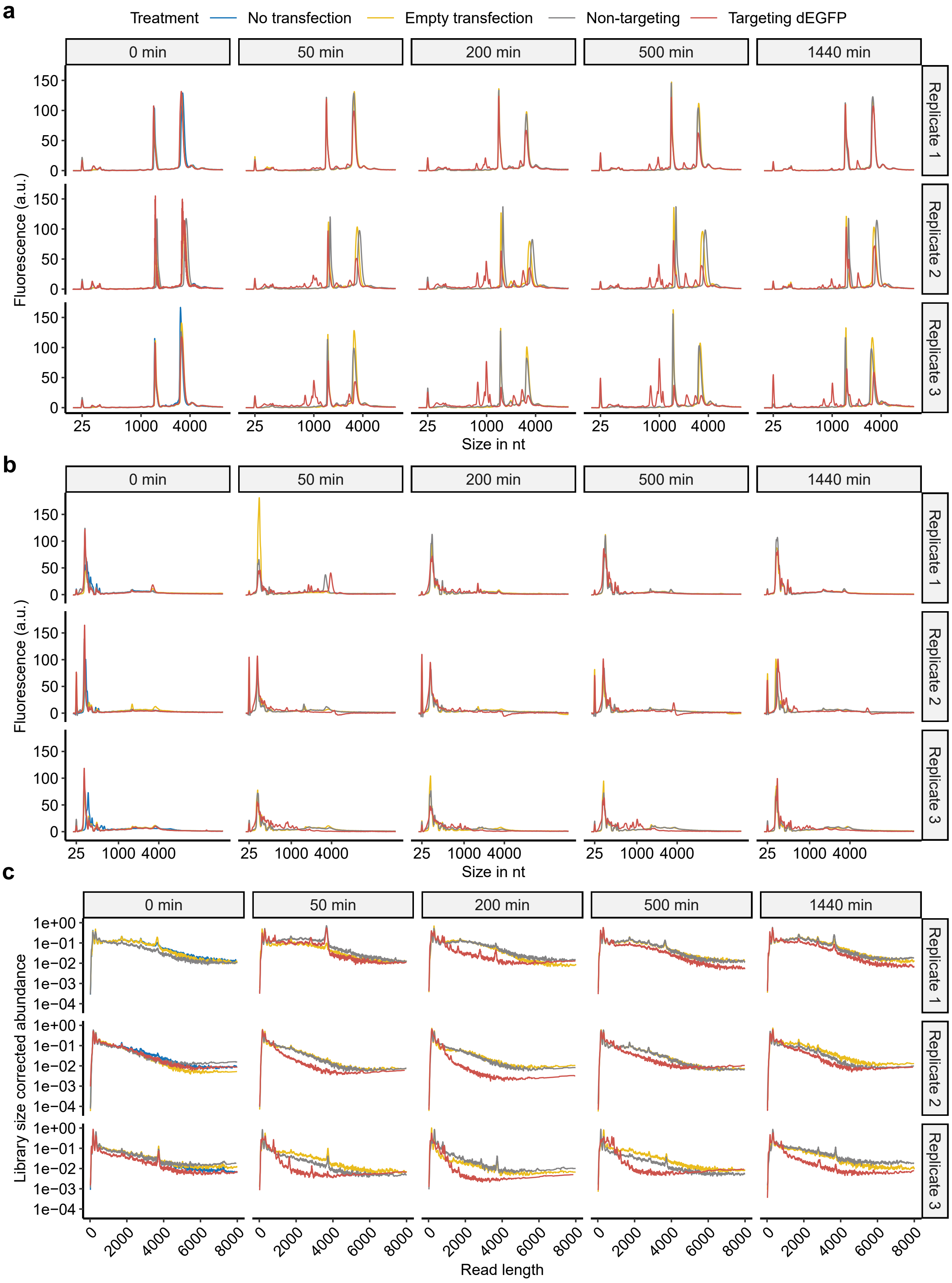

J. F. Bot et al. Supplementary Figure 11

Supplementary Figure 11. Nanopore library preparation and read lengths. a Bioanalyzer total RNA profiles of the samples send for Nanopore sequencing (n=3). Fluorescence was adjusted to the total area under the curve, to correct for differences in loaded amount of RNA. **b** Bioanalyzer RNA profile after *rRNA* depletion (n=3). Fluorescence was adjusted to the total area under the curve, to correct for differences in loaded amount of RNA. Legends same as **a.** **c** Rolling mean (k = 20) of the read lengths, normalized to the total amount of reads in each sample. The spike at ~4000 nt in the 50 minute replicate 1 sample is probably *rRNA* contamination. Legends same as **a.** n=3 except for 0 minutes after iTOP Targeting *dEGFP* where n=2.

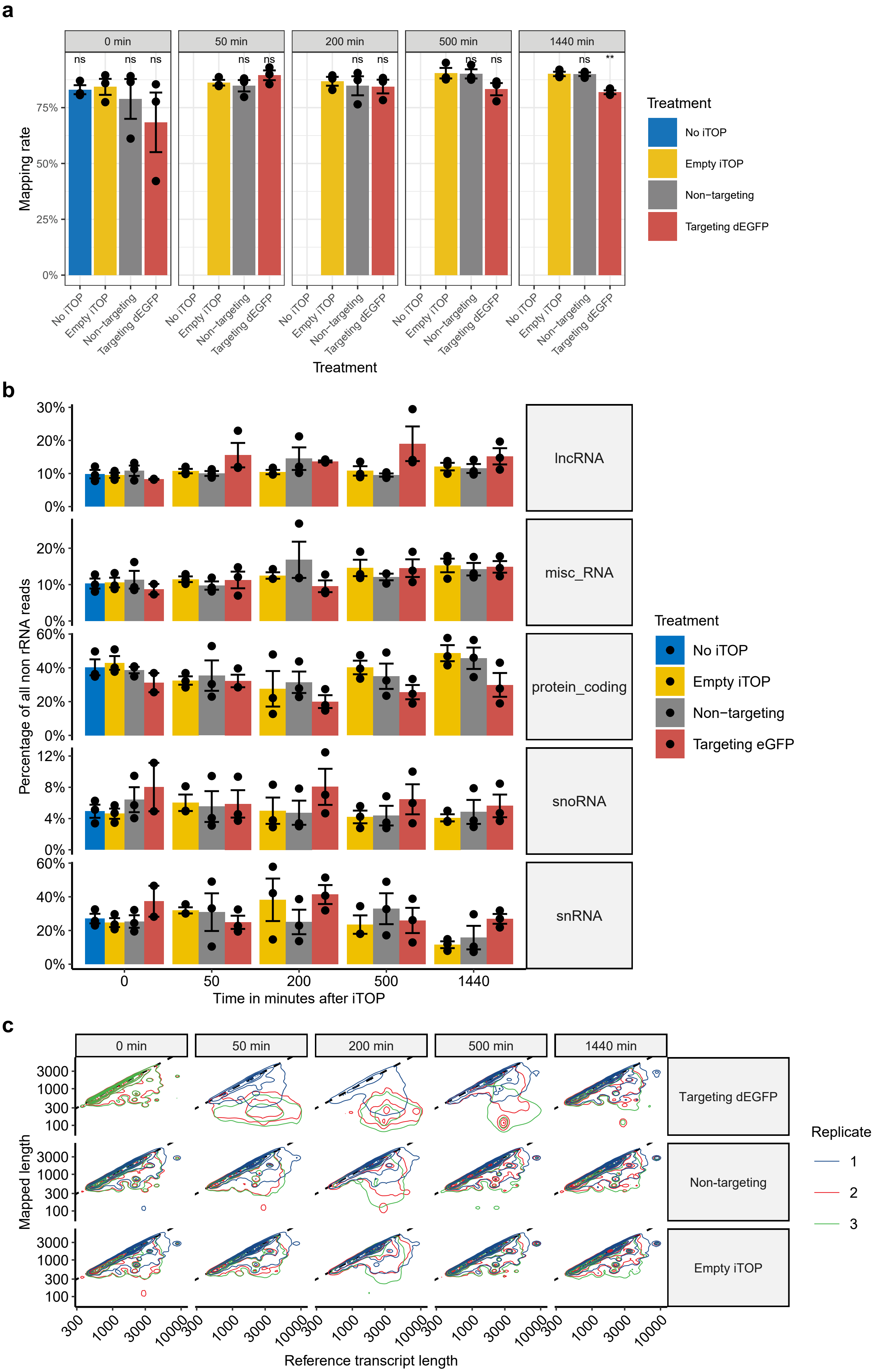

J. F. Bot et al. Supplementary Figure 12

Supplementary Figure 12. Nanopore sequencing analysis. a Mapping rates of the Nanopore sequencing reads (n=3). Reads were aligned to the transcriptome with minimap2, see methods. In replicate 1, the 0 minute after iTOP *dEGFP* targeting sample had a low mapping rate. This sample was excluded from all further analysis. At each timepoint, mapping rates of each treatment were compared to the empty iTOP condition using an unpaired, two-sided Welch's t-test (ns = not significant, ** < 0.01). **b** Percentage of all non *rRNA* reads that mapped to various transcript biotypes (n=3 except for 0 minutes after iTOP Targeting *dEGFP* where n=2). *rRNA* mapped reads were excluded due to variability in rRNA depletion efficiencies. **c** Density plots of mapped length versus reference transcript length by replicate of all reads mapped to protein coding transcripts, excluding histone genes and *WDR74* (n=3 except for 0 minutes after iTOP Targeting *dEGFP* where n=2).

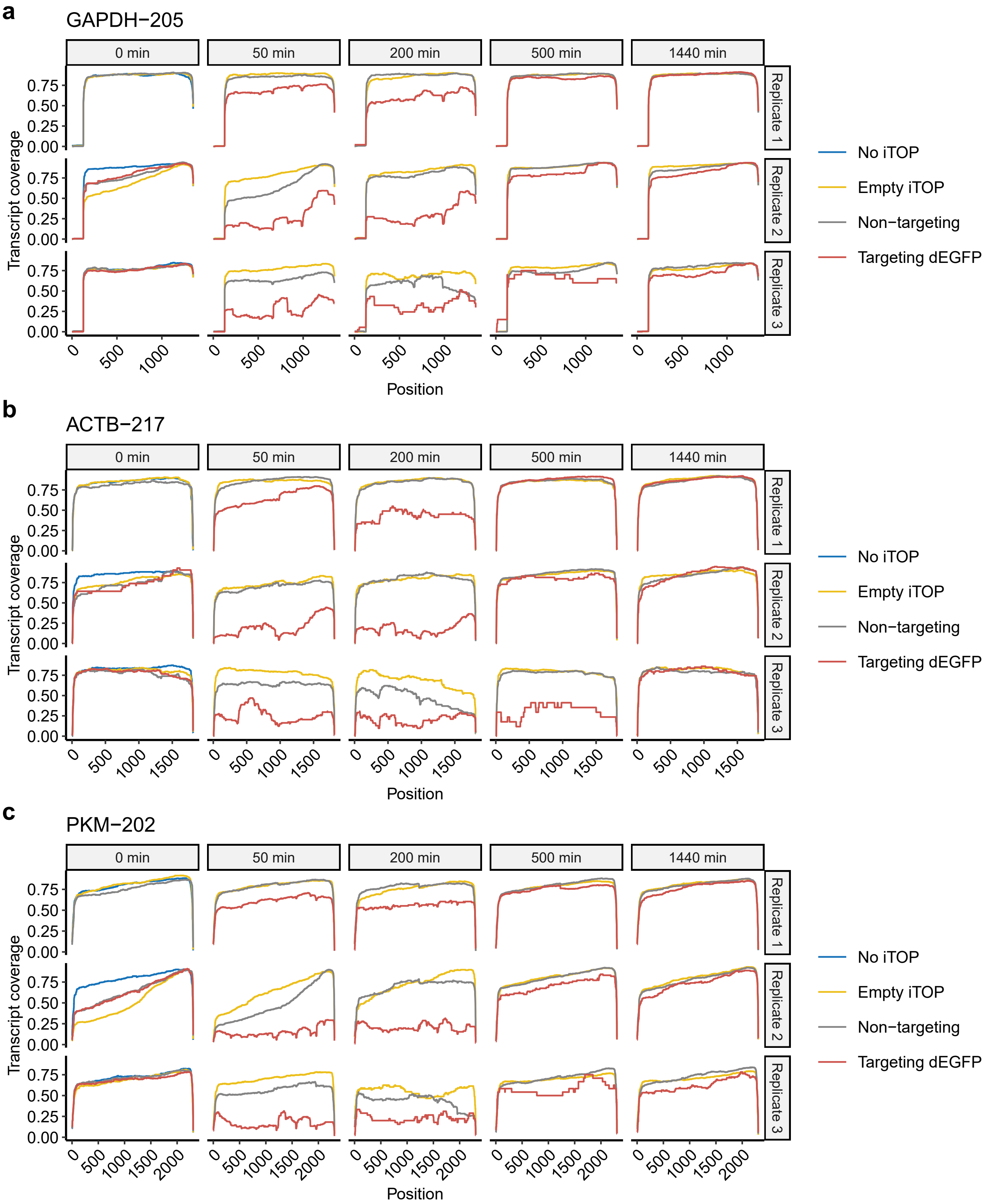

J. F. Bot et al. Supplementary Figure 13

Supplementary Figure 13. Relative transcript coverage of several mRNAs. **a** Nanopore sequencing relative transcript coverage of *GAPDH-205* (ENST00000396861.5), **b** *ACTB-217* (ENST00000646664.1) and **c** *PKM-202* (ENST00000335181.10). Columns show minutes after transfection. Relative transcript coverage values range from 0 to 1. A value of 0 means none of the reads mapped to this transcript cover that specific nucleotide position. A value of 1 means all reads mapped to this transcript cover that specific nucleotide. Thus, the more reads covering only part of the transcript, the lower the overall relative coverage along the entire transcript will be. We suspect dips in transcript coverage are LbuCas13a cleavage sites. n=3 except for 0 minutes after iTOP Targeting *dEGFP* where n=2.

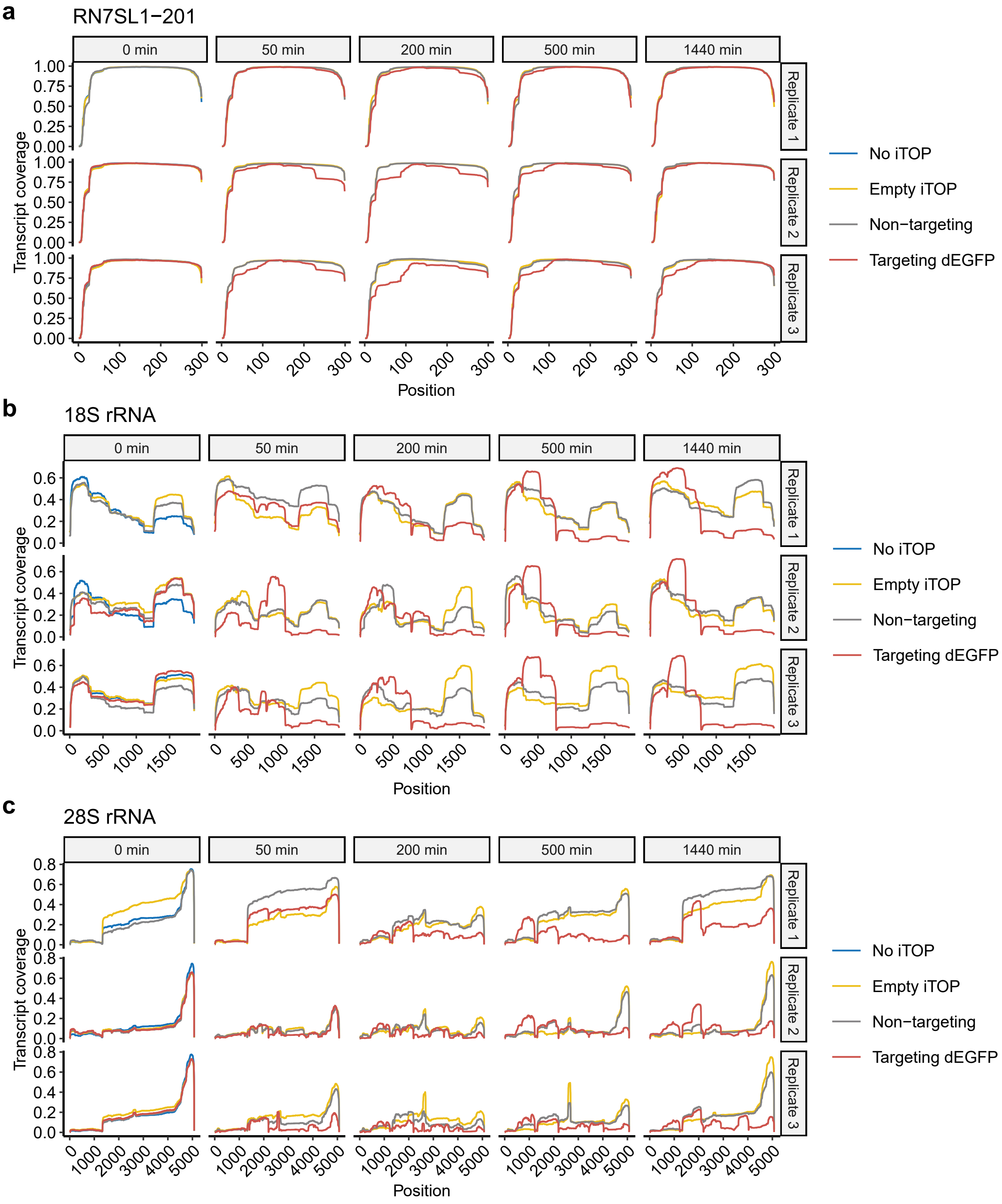

J. F. Bot et al. Supplementary Figure 14

Supplementary Figure 14. Relative transcript coverage of several cytoplasmic ncRNA. **a** Nanopore relative transcript coverage of *RN7SL1-201* (ENST00000618786.1), **b** *18S rRNA* (NR_003286.4) and **c** *28S rRNA* (NR_003287.4). Columns show minutes after transfection. Relative transcript coverage values range from 0 to 1. A value of 0 means none of the reads mapped to this transcript cover that specific nucleotide position. A value of 1 means all reads mapped to this transcript cover that specific nucleotide. Thus, the more reads covering only part of the transcript, the lower the overall relative coverage along the entire transcript will be. We suspect dips in transcript coverage are LbuCas13a cleavage sites. n=3 except for 0 minutes after iTOP Targeting *dEGFP* where n=2.

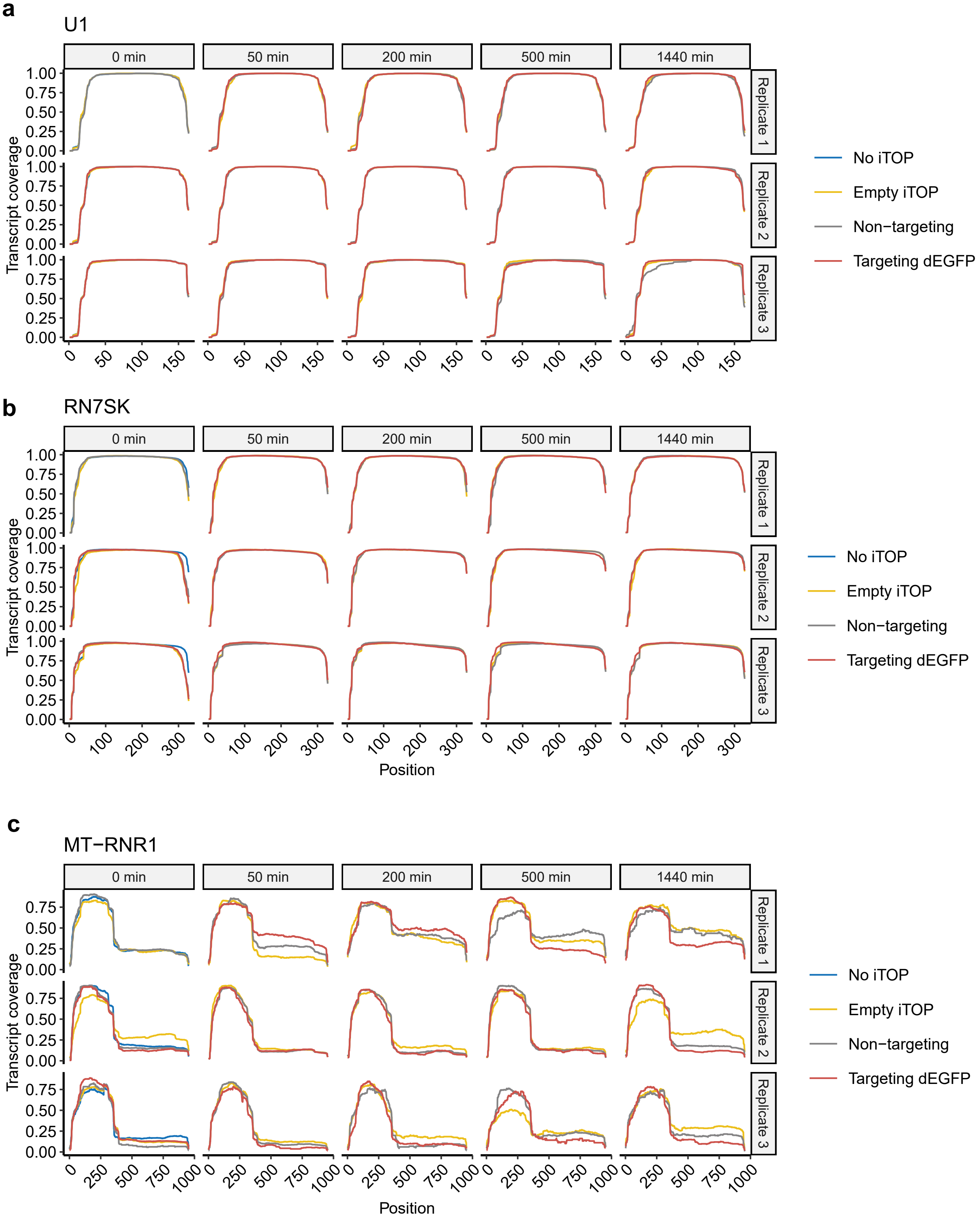

J. F. Bot et al. Supplementary Figure 15

Supplementary Figure 15. Relative transcript coverage of several nuclear and a mitochondrial RNA. **a** Relative transcript coverage of *U1* (ENST00000619109.1), **b** *RN7SK* (ENST00000636484.1) and **c** *MT-RNR1* (ENST00000389680.2). Columns show minutes after transfection. Relative transcript coverage values range from 0 to 1. A value of 0 means none of the reads mapped to this transcript cover that specific nucleotide position. A value of 1 means all reads mapped to this transcript cover that specific nucleotide. Thus, the more reads covering only part of the transcript, the lower the overall relative coverage along the entire transcript will be. The transcript coverage of these nuclear and mitochondrial RNAs does not seem to be affected by *dEGFP* targeting with LbuCas13a. n=3 except for 0 minutes after iTOP Targeting *dEGFP* where n=2.

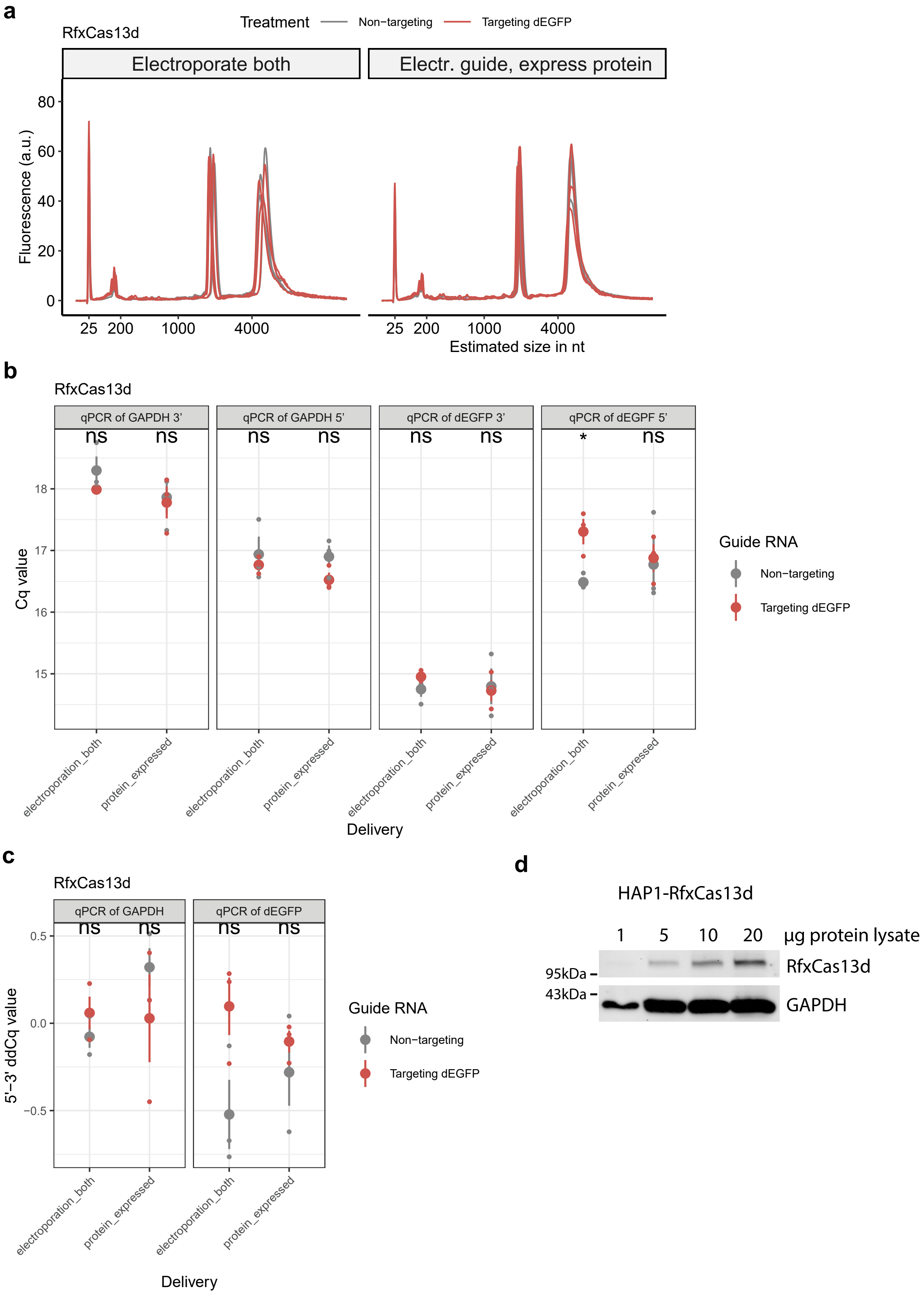

J. F. Bot et al. Supplementary Figure 16

Supplementary Figure 16. No collateral RNA cleavage was detected in RfxCas13d expressing HAP1-dEGFP cells. a Total RNA profiles 100 minutes after electroporation, as determined by bioanalyzer (n=3). Fluorescence was adjusted to the total area under the curve, to correct for differences in loaded amount of RNA. In the left facet 1 µM of RfxCas13d protein and guide RNA were electroporated into HAP1-dEGFP. In the right facet an RfxCas13d expressing HAP1-dEGFP cell line generated by lentiviral infection was electroporated with 1 µM of a non-targeting or *dEGFP* targeting gRNA. **b** Cq values from qPCR on samples from **a** using both a 5' and a 3' primer pair on GAPDH and dEGFP (n=3). All targeting treatments were compared with the non-targeting control using an unpaired two-sided Welch's t-test (ns = not significant, * = p-value < 0.05). **c** 5'-3' ddCq values (n=3) from the qPCR on the samples from **a**. All targeting treatments were compared with the non-targeting control using an unpaired two-sided Welch's t-test (ns = not significant). **d** Western blot after stable lentiviral integration of an RfxCas13d expression plasmids. An HA-tag antibody was used to detect RfxCas13d.

a

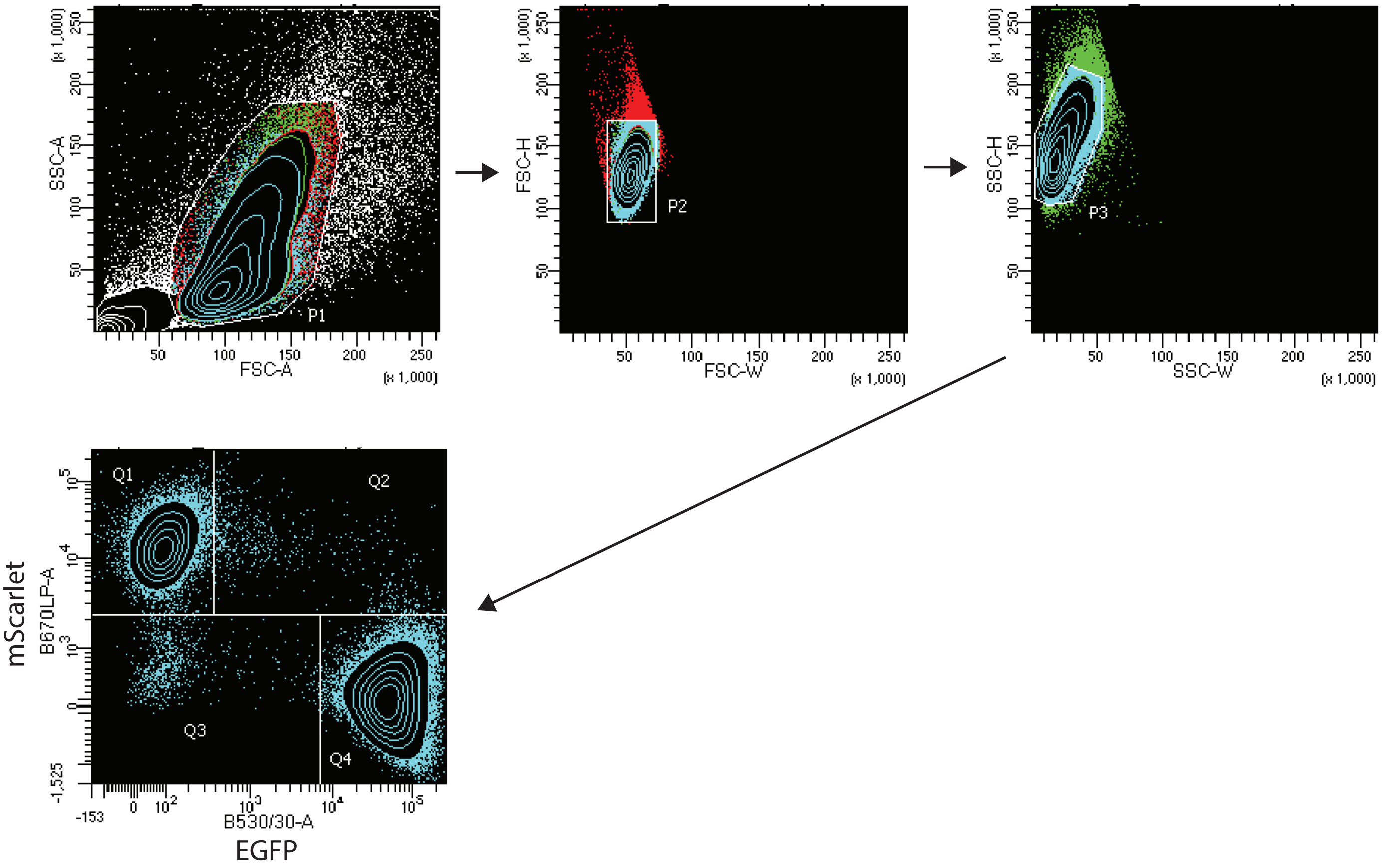

SSC-A
FSC-A
P1
FSC-H
FSC-W
P2
SSC-H
SSC-W
P3
(x 1,000)
Q1
Q2
Q3
Q4
mScarlet
B670LP-A
B530/30-A
EGFP

Supplementary Figure 17. Example gating strategy for the cell selection experiments.

a Here we show a typical gating strategy for assessing the percentage of fluorophore positive cells in the cell selection experiments. This example is from the experiment where we mixed RD-EGFP with RH30-mScarlet (Figure 4f). First cells are gated on FSC-A versus SSC-A (P1). Then, P2 and P3 are used to exclude doublets by gating on FSC width versus height and SSC width versus height respectively. Finally, the percentage of EGFP (Q4) and mScarlet (Q1) positive cells was assessed on the P3 population using a quadrant gate. To correct for spectral overlap between EGFP and mScarlet, we performed compensation, setting the overlap of EGFP on mScarlet (Y-axis in plot) to 12.8% and mScarlet on EGFP (X-axis in plot) to 1.4% in the compensation window of BD FACSDiva. In experiments where only one fluorophore positive population was present, the fluorophore of interest was plotted against an unrelated fluorophore and gates were set on this plot using a fully negative population. Plots were generated by the BD FACSDiva software.

a

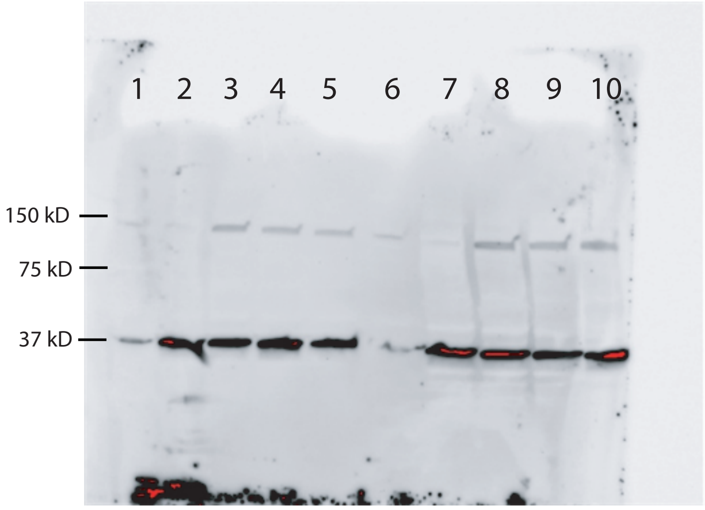

b

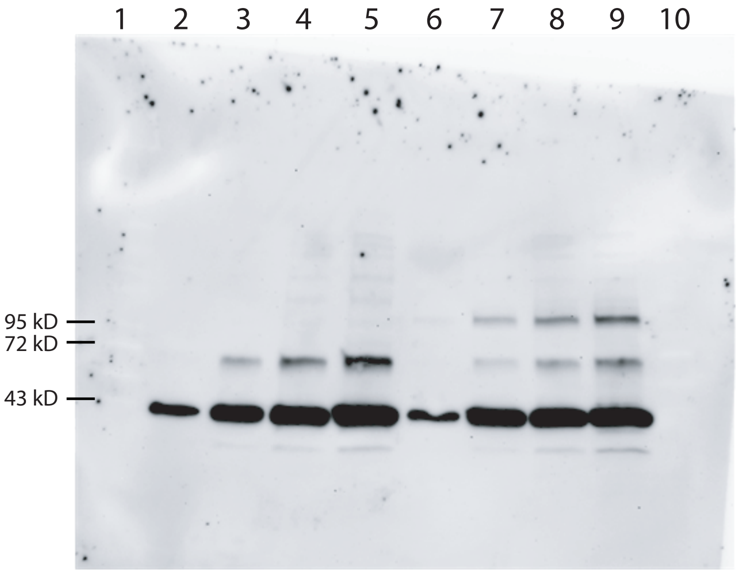

Supplementary Figure 18. Western blot source data. a The uncropped and unedited chemiluminescence image from the western blot shown in Supplementary Figure 5b. The Precision Plus Protein Dual Color Standards were loaded in lanes 1 and 6. Lysate from untreated HAP1-dEGFP control cells was loaded in lane 2. Lanes 3 to 5 were loaded with three independent replicates of transient LbuCas13a expression plasmid transfections of HAP1-dEGFP. Lanes 2 to 5 were loaded with 20 µg protein lysate. Lanes 7-10 were loaded with the same samples as lanes 2-5, however 50 µg protein lysate was used. Lanes 7-10 are not shown in Supplementary Figure 5b. **b** The uncropped and unedited chemiluminescence image from the western blot shown in Supplementary Figure 16d. The Color-coded Prestained Protein Marker, High Range (43-315 kDa), was loaded in lanes 1 and 10. Lanes 2 to 5 are not shown in Supplementary Figure 16d. These lanes were loaded with 1, 5, 10 and 20 µg lysate respectively of HAP1-dEGFP cells transfected with lentivirus containing an LbuCas13a expression plasmid. Lanes 6 to 9 were loaded with 1, 5, 10 and 20 µg lysate respectively of HAP1-dEGFP cells transfected with lentivirus containing an RfxCas13d expression plasmid. Cells had been selected for stable integration of the lentiviral transfer plasmid as per the methods.

Supplementary Note 1

We initially compared the differed Cas13 orthologs in cells at 50 minutes after transfection, while targeting dEGFP (Supplementary Figure 4b). However, from subsequent experiments with LbuCas13a we concluded that collateral RNA cleavage peaks at around 100 minutes after transfection (Figure 1), and that targeting the 18S rRNA resulted in the highest collateral cleavage activity in all tested cell types (Figure 2). We wondered whether the other Cas13 orthologs were able to show detectable levels of collateral cleavage under these conditions. Therefore, we re-tested LwaCas13a, BzCas13b and RspCas13d, this time isolating total RNA after 100 minutes and targeting either GAPDH, dEGFP or 18S rRNA in HAP1 cells. Targeting GAPDH or dEGFP with LwaCas13a did not change the total RNA profile, however 18S rRNA targeting resulted in two additional peaks smaller in size than the 18S rRNA (Supplementary Figure 2a). As 18S rRNA was the target RNA, this probably represents target RNA cleavage. Using BzCas13b resulted in a small extra peak between the 18S and 28S rRNA peak when targeting dEGFP or 18S rRNA (Supplementary Figure 2b). Furthermore, RNAs smaller than 5S RNA and tRNA appeared to increase in abundance, which possibly represents the emergence of cleavage products. None of the samples using RspCas13d showed any alteration to the total RNA profile, suggesting that RspCas13d does not work well in human cells (Supplementary Figure 2c). Thus, none of the other tested Cas13 orthologs were able to generate the dramatic RNA degradation pattern found when using LbuCas13a, although some collateral cleavage activity by LwaCas13a and BzCas13b cannot be completely ruled out.

Recently, it was reported that RfxCas13d exhibits collateral cleavage in eukaryotic cells[6,7]. Therefore, we aimed to explore how the *in vitro* and intracellular activity of RfxCas13d compared to the other orthologs tested in this study. First, we produced recombinant RfxCas13d protein (Supplementary Figure 1a) and assessed the collateral RNA cleavage activity of RfxCas13d *in vitro* with the RNaseAlert assay. RfxCas13d was not active in this assay for all three guide RNAs tested (Supplementary Figure 1c). It is possible that RfxCas13d, under these assay conditions, was not able to cleave the substrate provided by RNaseAlert. However, RfxCas13d has a similar cleavage preference as LbuCas13a, which seemingly has no issue cleaving the RNaseAlert reporter, both preferring a poly-U substrate[8–10]. Alternatively, the RfxCas13d recombinant protein production might have had a very low active fraction. In any case, we cannot draw any conclusions about the *in vitro* activity of RfxCas13d. Because our recombinant RfxCas13d protein seemed inactive, we made a HAP1-dEGFP derived RfxCas13d expressing cell line. We did not find any evidence of collateral activity in this RfxCas13d protein expressing line (Supplementary Figure 16). In

conclusion, we could not find any evidence of collateral RNA cleavage activity by RfxCas13d. Other recent publications also report not finding indications of collateral RNA cleavage by RfxCas13d in cells[11,12]. It is interesting to note that the recent reports of collateral cleavage by RfxCas13d in eukaryotic cell lines all use transient plasmid transfection, while above mentioned recent publications finding no collateral cleavage use lentiviral integration. This suggests that the intracellular (Rfx)Cas13 concentration is an important factor in whether collateral RNA cleavage occurs.

Altogether, these results highlight how situational the collateral RNA cleavage activity of Cas13 orthologs seems to be, depending on many factors such as delivery method, guide RNA, target RNA and cell line. This might explain the conflicting reports of collateral cleavage for orthologs such as LwaCas13a and RfxCas13d. Our work suggests that LbuCas13a may be the most reliable ortholog for the induction of collateral RNA cleavage in human cells, as we were able to find collateral cleavage in all tested cell lines, using different RNP delivery methods, and targeting different highly expressed transcripts.

Supplementary Table 1

Name	Ortholog	Sequence	Figure
**IVT_fw_T7_EGFP_target_RNA**	NA	ggcactctaatacgactcactataggatggtgagcaagggcg	SF1bc
**IVT_rev_EGFP_target_RNA**	NA	acttgtacagctcgtccatgcc	SF1bc
**IVT_T7_fwd_Lbu**	LbuCas13a	ggcactcTAATACGACTCACTATAGGACCAC	1abd, 2abc, 3ab, 4bce, 5abc, 6abcd, 7abc, SF1b, SF3ab, SF4b, SF5ac, SF6c, SF7d, SF9abcd, SF10abcdefgh, SF11abc, SF12abc, SF13abc, SF14abc, SF15abc
**IVT_temp_EGFP_SP1_Lbu**	LbuCas13a	TAATACGACTCACTATAGgaccaccccaaaaatgaaggggactaaaacactcgaccaggatgggcaccaccccggtg	3b
**IVT_rev_EGFP_SP1_Lbu**	LbuCas13a	caccggggtggtgcc	3b
**IVT_temp_EGFP_SP2_Lbu**	LbuCas13a	TAATACGACTCACTATAGgaccaccccaaaaatgaaggggactaaaactctgctggtagtggtcggcgagctgcac	1abd, 3ab, 4bce, 5abc, 6abcd, 7abc, SF1b, SF3ab, SF4b, SF5c, SF9abcd, SF10abcdefgh, SF11abc, SF12abc, SF13abc, SF14abc, SF15abc
**IVT_rev_EGFP_SP2_Lbu**	LbuCas13a	gtgcagctcgccgacc	1abd, 3ab, 4bce, 5abc, 6abcd, 7abc, SF1b, SF3ab, SF4b, SF5c, SF9abcd, SF10abcdefgh, SF11abc, SF12abc, SF13abc, SF14abc, SF15abc
**IVT_temp_EGFP_SP3_Lbu**	LbuCas13a	TAATACGACTCACTATAGgaccaccccaaaaatgaaggggactaaaacagtcacgaactccagcaggaccatgtgat	3b
**IVT_rev_EGFP_SP3_Lbu**	LbuCas13a	atcacatggtcctgctgg	3b
**IVT_temp_18S_Lbu**	LbuCas13a	TAATACGACTCACTATAGgaccaccccaaaaatgaaggggactaaaacgcgcccgtcggcatgtattagctctaga	2abc, SF5a, SF6c
**IVT_rev_18S_Lbu**	LbuCas13a	tctagagctaatacatgccgac	2abc, SF5a, SF6c
**IVT_temp_RPS19_Lbu**	LbuCas13a	TAATACGACTCACTATAGgaccaccccaaaaatgaaggggactaaaacCTGGTTCACGTCTTTTACAGTAACTCCA	2abc, SF5a, SF6c
**IVT_rev_RPS19_Lbu**	LbuCas13a	TGGAGTTACTGTAAAAGACGTGAAC	2abc, SF5a, SF6c

**IVT_temp_GAPDH_Lbu**	LbuCas13a	TAATACGACTCACTATAGgaccaccccaaaaatgaaggggactaaaacccagagttaaaagcagccctggtgacca	2abc, SF5a, SF6c
**IVT_rev_GAPDH_Lbu**	LbuCas13a	tggtcaccagggctgc	2abc, SF5a, SF6c
**IVT_temp_NT_Lbu**	LbuCas13a	TAATACGACTCACTATAGgaccaccccaaaaatgaaggggactaaaacatggatacttggtagaacagcaatcta	1d, 2abc, 3ab, 4bce, 5abc, 6abcd, 7abc, SF3ab, SF4b, SF5ac, SF6c, SF7d, SF9abcd, SF10abcdefgh, SF11abc, SF12abc, SF13abc, SF14abc, SF15abc
**IVT_rev_NT_lbu**	LbuCas13a	tagattgctgttctaccaagtaatcc	1d, 2abc, 3ab, 4bce, 5abc, 6abcd, 7abc, SF3ab, SF4b, SF5ac, SF6c, SF7d, SF9abcd, SF10abcdefgh, SF11abc, SF12abc, SF13abc, SF14abc, SF15abc
**IVT_T7_fwd_Lwa**	LwaCas13a	GGCACTCTAATACGACTCACTATAGgactaccc	SF1b, SF2a, SF4b
**IVT_temp_NT_Lwa**	LwaCas13a	TAATACGACTCACTATAGgactaccccaaaaacgaaggggactaaaacatggatacttggtagaacagcaatcta	SF2a, SF4b
**IVT_rev_NT_Lwa**	LwaCas13a	tagattgctgttctaccaagtaatcc	SF2a, SF4b
**IVT_temp_GAPDH_Lwa**	LwaCas13a	TAATACGACTCACTATAGgactaccccaaaaacgaaggggactaaaacccagagttaaaagcagccctggtgacca	SF2a
**IVT_rev_GAPDH_Lwa**	LwaCas13a	tggtcaccagggctgc	SF2a
**IVT_temp_EGFP_sp2_Lwa**	LwaCas13a	TAATACGACTCACTATAGgactaccccaaaaacgaaggggactaaaactctgctggtagtggtcggcgagctgcac	SF1b, SF2a, SF4b
**IVT_rev_EGFP_SP2_Lwa**	LwaCas13a	gtgcagctcgccgacc	SF1b, SF2a, SF4b
**IVT_temp_18S_Lwa**	LwaCas13a	TAATACGACTCACTATAGgactaccccaaaaacgaaggggactaaaacgcgcccgtcggcatgtattagctctaga	SF2a
**IVT_rev_18S_Lwa**	LwaCas13a	tctagagctaatacatgccgac	SF2a
**IVT_rev_Bz**	BzCas13b	GTTGTGATTACCCTCCAAAATGAG	SF1b, SF2b, SF4b
**IVT_temp_NT_Bz**	BzCas13b	TAATACGACTCACTATAGGatggattacttggtagaacagcaatctaTAGTTGGAACTGCTCTCATTTTGGAGGGTAATCACAAC	SF2b, SF4b
**IVT_fw_NT_Bz**	BzCas13b	ggcactcTAATACGACTCACTATAGGatggattac	SF2b, SF4b
**IVT_temp_GAPDH_Bz**	BzCas13b	TAATACGACTCACTATAGGccagagttaaaagcagccctggtgaccaggGTTGGAACTGCTCTCATTTTGGAGGGTAATCACAAC	SF2b

**IVT_fw_GAPDH_Bz**	BzCas13b	GGCACTCTAATACGACTCACTATAGGCCA	SF2b
**IVT_temp_EGFP_SP2_Bz**	BzCas13b	TAATACGACTCACTATAGGtgctggtagtggtcggcgagctgcacgctgGTTGGAACTGCTCTCATTTTGGAGGGTAATCACAAC	SF1b, SF2b, SF4b
**IVT_fw_EGFP_sp2_Bz**	BzCas13b	ggcactcTAATACGACTCACTATAGGtgctgg	SF1b, SF2b, SF4b
**IVT_temp_18S_Bz**	BzCas13b	TAATACGACTCACTATAGGagcgcccgtcggcatgtattagctctagaaGTTGGAACTGCTCTCATTTTGGAGGGTAATCACAAC	SF2b
**IVT_fw_18S_Bz**	BzCas13b	ggcactcTAATACGACTCACTATAGGagc	SF2b
**IVT_temp_EGFP_SP2_Psp**	PspCas13b	TAATACGACTCACTATAGGtgctggtagtggtcggcgagctgcacgctgGTtgtggaaggtccagttttgggggctattacaaca	SF1b, SF4b
**IVT_T7_fwd_EGPF_SP2_Psp**	PspCas13b	ggcactcTAATACGACTCACTATAGGtgctgg	SF1b, SF4b
**IVT_temp_NT_Psp**	PspCas13b	TAATACGACTCACTATAGGatggattacttggtagaacagcaatctaTAGTtgtggaaggtccagttttgggggctattacaaca	SF4b
**IVT_T7_fwd_NT_Psp**	PspCas13b	ggcactcTAATACGACTCACTATAGGatggattac	SF4b
**IVT_rev_Psp**	PspCas13b	tgttgtaatagcccccaaaactg	SF1b, SF4b
**IVT_T7_fwd_Rsp**	RspCas13d	GGCACTCTAATACGACTCACTATAGGCACTGG	SF1b, SF2c, SF4b
**IVT_temp_NT_Rsp**	RspCas13d	TAATACGACTCACTATAGGCACTGGTGCAAATTTGCACTAGTCTAAAACatggattacttggtagaacagcaatctatag	SF2c, SF4b
**IVT_rev_NT_Rsp**	RspCas13d	ctatagattgctgttctaccaag	SF2c, SF4b
**IVT_temp_GAPDH_Rsp**	RspCas13d	TAATACGACTCACTATAGGCACTGGTGCAAATTTGCACTAGTCTAAAACccagagttaaaagcagccctggtgaccaggc	SF2c
**IVT_rev_GAPDH_Rsp**	RspCas13d	gcctggtcaccaggg	SF2c
**IVT_temp_EGFP_SP2_Rsp**	RspCas13d	TAATACGACTCACTATAGGCACTGGTGCAAATTTGCACTAGTCTAAAACtctgctggtagtggtcggcgagctgcacgct	SF1b, SF2c, SF4b
**IVT_rev_EGFP_SP2_Rsp**	RspCas13d	agcgtgcagctcgcc	SF1b, SF2c, SF4b
**IVT_temp_18S_Rsp**	RspCas13d	TAATACGACTCACTATAGGCACTGGTGCAAATTTGCACTAGTCTAAAACgcgcccgtcggcatgtattagctctagaatt	SF2c
**IVT_rev_18S_Rsp**	RspCas13d	aattctagagctaatacatgccg	SF2c
**IVT_temp_targeting EGFP△fluor**	LbuCas13a	TAATACGACTCACTATAGGGACCACCCCAAAAATGAAGGGGACTAAAACcactgcaccagggtggtcacgagggtgg	SF7d
**IVT_rev_targeting EGFP△fluor**	LbuCas13a	ccaccctcgtgaccacc	SF7d

**IVT_T7_fwd_Rfx**	RfxCas13d	ggcactcTAATACGACTCACTATAGGaacc	SF1b, SF16abc
**IVT_temp_NT_Rfx**	RfxCas13d	TAATACGACTCACTATAGGaacccctaccaactggtcggggtttgaaacatggattacttggtagaacagcaatcta	SF1b, SF16abc
**IVT_rev_NT_Rfx**	RfxCas13d	tagattgctgttctaccaagtaatcc	SF1b, SF16abc
**IVT_temp_EGFP_SP1_Rfx**	RfxCas13d	TAATACGACTCACTATAGGaacccctaccaactggtcggggtttgaaacgtccagctcgaccaggatgggcaccacc	SF1b
**IVT_rev_EGFP_SP1_Rfx**	RfxCas13d	ggtggtgcccatcctg	SF1b
**IVT_temp_EGFP_SP2_Rfx**	RfxCas13d	TAATACGACTCACTATAGGaacccctaccaactggtcggggtttgaaactctgctggtagtggtcggcgagctgcac	SF1b, SF16abc
**IVT_rev_EGFP_SP2_Rfx**	RfxCas13d	gtgcagctcgccgacc	SF1b, SF16abc
**IVT_temp_EGFP_SP3_Rfx**	RfxCas13d	TAATACGACTCACTATAGGaacccctaccaactggtcggggtttgaaacgtcacgaactccagcaggaccatgtgat	SF1b
**IVT_rev_EGFP_SP3_Rfx**	RfxCas13d	atcacatggtcctgctgg	SF1b
**IDT_NT_Lbu**	LbuCas13a	rGrArCrCrArCrCrCrCrArArArArArUrGrArArGrGrGrGrArCrUrArArArArCrArUrGrGrArUrUrArCrUrUrGrGrUrArGrArArCrArGrCrArArUrCrUrA	4f, SF8bcdef
**IDT_EGFP_SP2**	LbuCas13a	rGrArCrCrArCrCrCrCrArArArArArUrGrArArGrGrGrGrArCrUrArArArArCrUrCrUrGrCrUrGrGrUrArGrUrGrGrUrCrGrGrCrGrArGrCrUrGrCrArC	4f
**IDT_CDK4_SP1**	LbuCas13a	rGrArCrCrArCrCrCrCrArArArArArUrGrArArGrGrGrGrArCrUrArArArArCrGrArUrCrUrCrGrGrUrGrArArCrGrArUrGrCrArArUrUrGrGrCrArUrG	4f, SF8bcdef
**IDT_CDK4_SP2**	LbuCas13a	rGrArCrCrArCrCrCrCrArArArArArUrGrArArGrGrGrGrArCrUrArArArArCrGrUrUrCrCrUrArCrGrGrCrCrCrCrArUrArCrArCrCrCrGrArGrCrUrC	SF8bc
**IDT_CDK4_SP3**	LbuCas13a	rGrArCrCrArCrCrCrCrArArArArArUrGrArArGrGrGrGrArCrUrArArArArCrUrArUrCrGrArGrArGrGrUrArGrCrCrArUrUrCrUrCrArGrArUrCrArA	SF8bc
**IDT_CDK4_SP4**	LbuCas13a	rGrArCrCrArCrCrCrCrArArArArArUrGrArArGrGrGrGrArCrUrArArArArCrCrArUrGrCrUrCrArArArCrArCrCrArGrGrGrUrUrArCrCrUrUrGrArU	SF8bc
**IDT_CDK4_SP5**	LbuCas13a	rGrArCrCrArCrCrCrCrArArArArArUrGrArArGrGrGrGrArCrUrArArArArCrUrCrArArGrGrArUrCrUr	SF8bc

		GrArUrGrCrGrCrCrArGrUrUrUrCrUrArArG	

Supplementary Table 2

Name	Sequence	Source	Figure
**233_Anchored oligo dT**	TTTTTTTTTTTTTTTTTTTTTVN	this paper	1d, 2c, SF4ab, SF16bc, SF17bc, and RT primer for nanopore library prep
**240_qPCR GAPDH 3' fw**	agtccctgccacactcag	https://www.sigmaaldrich.com/technical-documents/protocols/biology/3-5-assay-for-analysis-of-rna-integrity.html	1d, 2c, SF4ab, SF8a, SF16bc, SF17bc
**241_qPCR GAPDH 3' rv**	tactttattgatggtacatgacaagg	https://www.sigmaaldrich.com/technical-documents/protocols/biology/3-5-assay-for-analysis-of-rna-integrity.html	1d, 2c, SF4ab, SF8a, SF16bc, SF17bc
**242_qPCR GAPDH 5' fw**	gtgaaccatgagaagtatgacaac	https://www.sigmaaldrich.com/technical-documents/protocols/biology/3-5-assay-for-analysis-of-rna-integrity.html	1d, 2c, SF4ab, SF16bc, SF17bc
**243_qPCR GAPDH 5' rv**	catgagtccttccacgatacc	https://www.sigmaaldrich.com/technical-documents/protocols/biology/3-5-assay-for-analysis-of-rna-integrity.html	1d, 2c, SF4ab, SF16bc, SF17bc
**244_qPCR eGFP_IRIS_PuroR_WPRE 3' fw**	tgtcggggaaatcatcgtcc	this paper	1d, SF4ab, SF16bc, SF17bc
**245_qPCR eGFP_IRIS_PuroR_WPRE 3' rv**	aaggaaggtccgctggattg	this paper	1d, SF4ab, SF16bc, SF17bc
**246_qPCR eGFP_IRIS_PuroR_WPRE 5' fw**	CTACCCCGACCACATGAAGC	this paper	1d, SF4ab, SF16bc, SF17bc

**247_qPCR eGFP_IRIS_PuroR_WPRE 5' rv**	AAGAAGATGGTGCGCTCCTG	this paper	1d, SF4ab, SF16bc, SF17bc
**qPCR_eGFP_fw**	gaccctgaagttcatctgc	this paper	4e, SF7b
**qPCR_eGFP_rv**	gtgctgcttcatgtggtc	this paper	4e, SF7b
**qPCR_GAPDH_fw**	GTCTCCTCTGACTTCAACAGCG	this paper	4e, SF7b
**qPCR_GAPDH_rv**	ACCACCCTGTTGCTGTAGCCAA	this paper	4e, SF7b
**574_qpcr cdk4 fw**	ctgtgccacatcccgaactg	this paper	SF8a
**575_qpcr cdk4 rev**	gcctcttagaaactggcgca	this paper	SF8a

Supplementary Table 3

Name	Sequence	Source	Figure
**crRNA for AsCas12a**	UAAUUUCUACUCUUGUAGAUAGCCGCUACCCCGACCACAUGAA	Zhao et al. 2022	SF7cd
**Repair insert (sense strand)**	GCCACCTACGGCAAGCTGACCCTGAAATTCATCTGCACCACCGGCAAGCTGCCCGTGCCCTGGCCCACCCTCGTGACCACCCTGACCTACGGCGTGCAGTGTTTCAGCCGCTACCCCGACCAC	Zhao et al. 2022	SF7cd
**Repair insert (antisense strand)**	TCATGTGGTCGGGGTAGCGGCTGAAACACTGCACCAGGGTGGTCACGAGGGTGGGCCAGGGCACGGGCAGCTTGCCGGTGGTGCAGATGAATTTCAGGGTCAGCTTGCCGTAGG	Zhao et al. 2022	SF7cd

Supplementary Table 4

**Name**: pEF-1a_dEGFP-Y66S-IRES-PuroR-WPRE (Addgene ID = 251210)
**Map**:

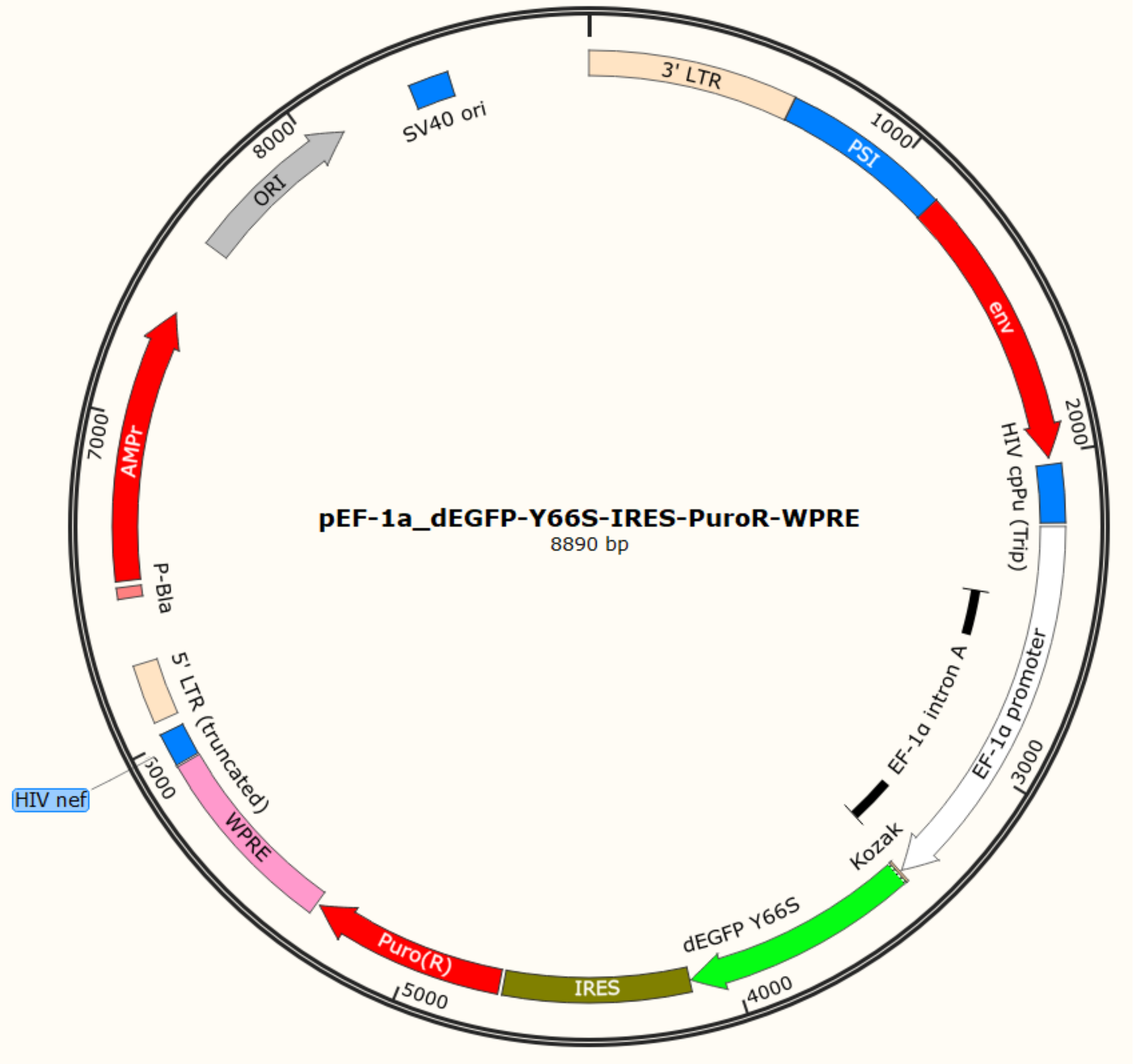

Sequence:

tggaagggctaattcactcccaaagaagacaagatatccttgatctgtggatctaccacacacaaggctacttccctgattagcagaactacacaccagggccaggggtcag
atatccactgacctttggatggtgctacaagctagtaccagttgagccagataaggtagaagaggccaataaaggagagaacaccagcttgttacaccctgtgagcctgcatg
ggatggatgacccggagagagaagtgttagagtggaggtttgacagccgcctagcatttcatcacgtggcccgagagctgcatccggagtacttcaagaactgctgatatcga
gcttgctacaagggactttccgctggggactttccagggaggcgtggcctgggcgggactggggagtggcgagccctcagatcctgcatataagcagctgctttttgcctgtact
gggtctctctggttagaccagatctgagcctgggagctctctggctaactagggaacccactgcttaagcctcaataaagcttgccttgagtgcttcaagtagtgtgtgcccgtctgt
tgtgtgactctggtaactagagatccctcagacccttttagtcagtgtggaaaatctctagcagtggcgcccgaacagggacttgaaagcgaaagggaaaccagaggagctct
ctcgacgcaggactcggcttgctgaagcgcgcacggcaagaggcgaggggcggcgactggtgagtacgccaaaaattttgactagcggaggctagaaggagagagatg
ggtgcgagagcgtcagtattaagcgggggagaattagatcgcgatgggaaaaaattcggttaaggccagggggaaagaaaaaatataaattaaaacatatagtatgggca
agcagggagctagaacgattcgcagttaatcctggcctgttagaaacatcagaaggctgtagacaaatactgggacagctacaaccatcccttcagacaggatcagaagaa
cttagatcattatataatacagtagcaaccctctattgtgtgcatcaaaggatagagataaaagacaccaaggaagctttagacaagatagaggaagagcaaaacaaaagta
agaccaccgcacagcaagcggccggccgctgatcttcagacctggaggaggagatatgagggacaattggagaagtgaattatataaatataaagtagtaaaaattgaac
cattaggagtagcacccaccaaggcaaagagaagagtggtgcagagagaaaaaagagcagtgggaataggagctttgttccttgggttcttgggagcagcaggaagcact
atgggcgcagcgtcaatgacgctgacggtacaggccagacaattattgtctggtatagtgcagcagcagaacaatttgctgagggctattgaggcgcaacagcatctgttgca
actcacagtctggggcatcaagcagctccaggcaagaatcctggctgtggaaagatacctaaaggatcaacagctcctggggatttggggttgctctggaaaactcatttgcac
cactgctgtgccttggaatgctagttggagtaataaatctctggaacagatttggaatcacacgacctggatggagtgggacagagaaattaacaattacacaagcttaatacac
tccttaattgaagaatcgcaaaaccagcaagaaaagaatgaacaagaattattggaattagataaatgggcaagtttgtggaattggtttaacataacaaattggctgtggtatat
aaaattattcataatgatagtaggaggcttggtaggtttaagaatagtttttgctgtactttctatagtgaatagagttaggcagggatattcaccattatcgtttcagacccacctccca
accccgaggggacccgacaggcccgaaggaatagaagaagaaggtggagagagagacagagacagatccattcgattagtgaacggatctcgacggtatcgccgaatt
cacaaatggcagtattcatccacaattttaaaagaaaaggggggattggggggtacagtgcaggggaaagaatagtagacataatagcaacagacatacaaactaaaga

attacaaaaacaaattacaaaaattcaaaattttcgggtttattacagggacagcagagatccagtttggactagtcgtgaggctccggtgcccgtcagtgggcagagcgcaca
tcgcccacagtccccgagaagttggggggaggggtcggcaattgaaccggtgcctagagaaggtggcgcggggtaaactgggaaagtgatgtcgtgtactggctccgccttt
ttcccgagggtgggggagaaccgtatataagtgcagtagtcgccgtgaacgttctttttcgcaacgggtttgccgccagaacacaggtaagtgccgtgtgtggttcccgcgggcc
tggcctctttacgggttatggcccttgcgtgccttgaattacttccacctggctgcagtacgtgattcttgatcccgagcttcgggttggaagtgggtgggagagttcgaggccttgcg
cttaaggagccccttcgcctcgtgcttgagttgaggcctggcctgggcgctggggccgccgcgtgcgaatctggtggcaccttcgcgcctgtctcgctgctttcgataagtctctag
ccatttaaaatttttgatgacctgctgcgacgctttttttctggcaagatagtcttgtaaatgcgggccaagatctgcacactggtatttcggtttttggggccgcgggcggcgacggg
gcccgtgcgtcccagcgcacatgttcggcgaggcggggcctgcgagcgcggccaccgagaatcggacgggggtagtctcaagctggccggcctgctctggtgcctggcctc
gcgccgccgtgtatcgccccgccctgggcggcaaggctggcccggtcggcaccagttgcgtgagcggaaagatggccgcttcccggccctgctgcagggagctcaaaatg
gaggacgcggcgctcgggagagcgggcgggtgagtcacccacacaaaggaaaagggcctttccgtcctcagccgtcgcttcatgtgactccacggagtaccgggcgccgt
ccaggcacctcgattagttctcgagcttttggagtacgtcgtctttaggttggggggaggggttttatgcgatggagtttccccacactgagtgggtggagactgaagttaggccag
cttggcacttgatgtaattctccttggaatttgcccttttttgagtttggatcttggttcattctcaagcctcagacagtggttcaaagtttttttcttccatttcaggtgtcgtgaagcggccgcc
accatggtgagcaagggcgaggagctgttcaccggggtggtgcccatcctggtcgagctggacggcgacgtaaacggccacaagttcagcgtgtccggcgagggcgagg
gcgatgccacctacggcaagctgacCCtgaagttcatctgcaccaccggcaagctgcccgtgccctggcccacTTtGgtgaccaccctgacctCcggcgtgcagtgcttc
agccgctaccccgaccacatgaagcagcacgacttcttcaagtccgccatgcccgaaggctacgtccaggagcgcaccatcttcttcaaggacgacggcaactacaagacc
cgcgccgaggtgaagttcgagggcgacaccctggtgaaccgcatcgagctgaagggcatcgacttcaaggaggacggcaacatcctggggcacaagctggagtacaact
acaacagccacaacgtctatatcatggccgacaagcagaagaacggcatcaaggtgaacttcaagatccgccacaacatcgaggacggcagcgtgcagctcgccgacc
actaccagcagaacacccccatcggcgacggccccgtgctgctgcccgacaaccactacctgagcacccagtccgccctgagcaaagaccccaacgagaagcgcgatc
acatggtcctgctggagttcgtgaccgccgccgggatcactctcggcatggacgagctgtacaagtaaggatccctcccccccccctaacgttactggccgaagccgcttgga
ataaggccggtgtgcgtttgtctatatgttattttccaccatattgccgtcttttggcaatgtgagggcccggaaacctggccctgtcttcttgacgagcattcctaggggtctttcccctct
cgccaaaggaatgcaaggtctgttgaatgtcgtgaaggaagcagttcctctggaagcttcttgaagacaaacaacgtctgtagcgaccctttgcaggcagcggaaccccccca
cctggcgacaggtgcctctgcggccaaaagccacgtgtataagatacacctgcaaaggcggcacaaccccagtgccacgttgtgagttggatagttgtggaaagagtcaaat
ggctctcctcaagcgtattcaacaaggggctgaaggatgcccagaaggtaccccattgtatgggatctgatctggggcctcggtgcacatgctttacatgtgtttagtcgaggttaa
aaaaacgtctaggccccccgaaccacggggacgtggttttcctttgaaaaacacgatgataatatggccacacatatgaccgagtacaagcccacggtgcgcctcgccaccc
gcgacgacgtccccagggccgtacgcaccctcgccgccgcgttcgccgactaccccgccacgcgccacaccgtcgatccggaccgccacatcgagcgggtcaccgagct
gcaagaactcttcctcacgcgcgtcgggctcgacatcggcaaggtgtgggtcgcggacgacggcgccgcggtggcggtctggaccacgccggagagcgtcgaagcgggg
gcggtgttcgccgagatcggcccgcgcatggccgagttgagcggttcccggctggccgcgcagcaacagatggaaggcctcctggcgccgcaccggcccaaggagcccg
cgtggttcctggccaccgtcggcgtctcgcccgaccaccagggcaagggtctgggcagcgccgtcgtgctccccggagtggaggcggccgagcgcgccggggtgcccgcc
ttcctggagacctccgcgccccgcaacctccccttctacgagcggctcggcttcaccgtcaccgccgacgtcgaggtgcccgaaggaccgcgcacctggtgcatgacccgca
agcccggtgcctgaatcgatagatcctaatcaacctctggattacaaaatttgtgaaagattgactggtattcttaactatgttgctccttttacgctatgtggatacgctgctttaatgcc
tttgtatcatgctattgcttcccgtatggctttcattttctcctccttgtataaatcctggttgctgtctctttatgaggagttgtggcccgttgtcaggcaacgtggcgtggtgtgcactgtgttt
gctgacgcaacccccactggttggggcattgccaccacctgtcagctcctttccgggactttcgctttccccctccctattgccacggcggaactcatcgccgcctgccttgcccgc
tgctggacaggggctcggctgttgggcactgacaattccgtggtgttgtcggggaaatcatcgtcctttccttggctgctcgcctgtgttgccacctggattctgcgcgggacgtcctt
ctgctacgtcccttcggccctcaatccagcggaccttccttcccgcggcctgctgccggctctgcggcctcttccgcgtcttcgccttcgccctcagacgagtcggatctccctttggg
ccgcctccccgcctggtacctttaagaccaatgacttacaaggcagctgtagatcttagccactttttaaaagaaaaggggggactggaagggctaattcactcccaacgaag
acaagatcacctgcaggacaggcgcgccctgctttttgcttgtactgggtctctctggttagaccagatctgagcctgggagctctctggctaactagggaacccactgcttaagc
ctcaataaagcttgccttgagtgcttcaagtagtgtgtgcccgtctgttgtgtgactctggtaactagagatccctcagacccttttagtcagtgtggaaaatctctagcacccgggcg
attaaggaaagggctagatcattcttgaagacgaaagggcctcgtgatacgcctatttttataggttaatgtcatgataataatggtttcttagacgtcaggtggcacttttcggggaa
atgtgcgcggaacccctatttgtttatttttctaaatacattcaaatatgtatccgctcatgagacaataaccctgataaatgcttcaataatattgaaaaaggaagagtatgagtattc
aacatttccgtgtcgcccttattcccttttttgcggcattttgccttcctgtttttgctcacccagaaacgctggtgaaagtaaaagatgctgaagatcagttgggtgcacgagtgggtta
catcgaactggatctcaacagcggtaagatccttgagagttttcgccccgaagaacgttttccaatgatgagcacttttaaagttctgctatgtggcgcggtattatcccgtgttgacg
ccgggcaagagcaactcggtcgccgcatacactattctcagaatgacttggttgagtactcaccagtcacagaaaagcatcttacggatggcatgacagtaagagaattatgc
agtgctgccataaccatgagtgataacactgcggccaacttacttctgacaacgatcggaggaccgaaggagctaaccgcttttttgcacaacatgggggatcatgtaactcgc
cttgatcgttgggaaccggagctgaatgaagccataccaaacgacgagcgtgacaccacgatgcctgtagcaatggcaacaacgttgcgcaaactattaactggcgaacta
cttactctagcttcccggcaacaattaatagactggatggaggcggataaagttgcaggaccacttctgcgctcggcccttccggctggctggtttattgctgataaatctggagcc
ggtgagcgtgggtctcgcggtatcattgcagcactggggccagatggtaagccctcccgtatcgtagttatctacacgacggggagtcaggcaactatggatgaacgaaatag
acagatcgctgagataggtgcctcactgattaagcattggtaactgtcagaccaagtttactcatatatactttagattgatttaaaacttcatttttaatttaaaaggatctaggtgaag
atcctttttgataatctcatgaccaaaatcccttaacgtgagttttcgttccactgagcgtcagaccccgtagaaaagatcaaaggatcttcttgagatcctttttttctgcgcgtaatctg
ctgcttgcaaacaaaaaaaccaccgctaccagcggtggtttgtttgccggatcaagagctaccaactctttttccgaaggtaactggcttcagcagagcgcagataccaaatact
gttcttctagtgtagccgtagttaggccaccacttcaagaactctgtagcaccgcctacatacctcgctctgctaatcctgttaccagtggctgctgccagtggcgataagtcgtgtct
taccgggttggactcaagacgatagttaccggataaggcgcagcggtcgggctgaacggggggttcgtgcacacagcccagcttggagcgaacgacctacaccgaactga

gatacctacagcgtgagctatgagaaagcgccacgcttcccgaagggagaaaggcggacaggtatccggtaagcggcagggtcggaacaggagagcgcacgaggga
gcttccagggggaaacgcctggtatctttatagtcctgtcgggtttcgccacctctgacttgagcgtcgatttttgtgatgctcgtcaggggggcggagcctatggaaaaacgccag
caacgcggcctttttacggttcctggccttttgctggccttttgctcacatgttctttcctgcgttatcccctgattctgtggataaccgtattaccgcctttgagtgagctgataccgctcgc
cgcagccgaacgaccgagcgcagcgagtcagtgagcgaggaagcggaagagcgcccaatacgcaaaccgcctctccccgcgcgttggccgattcattaatgcagcaag
ctcatggctgactaattttttttatttatgcagaggccgaggccgcctcggcctctgagctattccagaagtagtgaggaggcttttttggaggcctaggcttttgcaaaaagctcccc
gtggcacgacaggtttcccgactggaaagcgggcagtgagcgcaacgcaattaatgtgagttagctcactcattaggcaccccaggctttacactttatgcttccggctcgtatgt
tgtgtggaattgtgagcggataacaatttcacacaggaaacagctatgacatgattacgaatttcacaaataaagcatttttttcactgcattctagttgtggtttgtccaaactcatca
atgtatcttatcatgtctggatcaactggataactcaagctaaccaaaatcatcccaaacttcccaccccataccctattaccactgccaattacctgtggtttcatttactctaaacct
gtgattcctctgaattattttcattttaaagaaattgtatttgttaaatatgtactacaaacttagtagt

Name: pEF-1a_EGFP-IRES-PuroR-WPRE (Addgene ID = 251211)

Map:

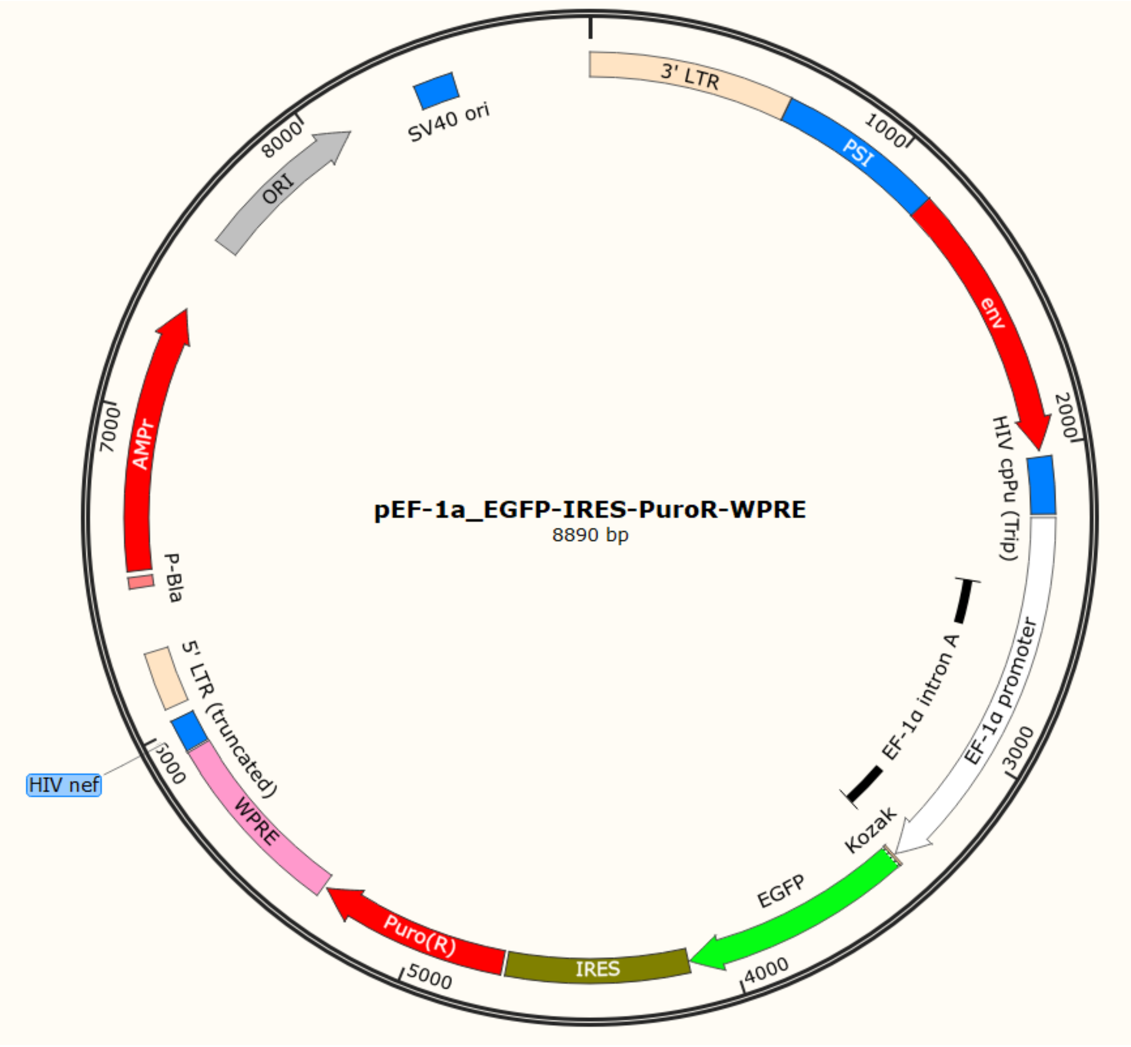

Sequence:

tggaagggctaattcactcccaaagaagacaagatatccttgatctgtggatctaccacacacaaggctacttccctgattagcagaactacacaccagggccaggggtcag
atatccactgacctttggatggtgctacaagctagtaccagttgagccagataaggtagaagaggccaataaaggagagaacaccagcttgttacaccctgtgagcctgcatg
ggatggatgacccggagagagaagtgttagagtggaggtttgacagccgcctagcatttcatcacgtggcccgagagctgcatccggagtacttcaagaactgctgatatcga
gcttgctacaagggactttccgctggggactttccagggaggcgtggcctgggcgggactggggagtggcgagccctcagatcctgcatataagcagctgctttttgcctgtact
gggtctctctggttagaccagatctgagcctgggagctctctggctaactagggaacccactgcttaagcctcaataaagcttgccttgagtgcttcaagtagtgtgtgcccgtctgt
tgtgtgactctggtaactagagatccctcagacccttttagtcagtgtggaaaatctctagcagtggcgcccgaacagggacttgaaagcgaaagggaaaccagaggagctct

ctcgacgcaggactcggcttgctgaagcgcgcacggcaagaggcgaggggcggcgactggtgagtacgccaaaaattttgactagcggaggctagaaggagagagatg
ggtgcgagagcgtcagtattaagcgggggagaattagatcgcgatgggaaaaaattcggttaaggccagggggaaagaaaaaatataaattaaaacatatagtatgggca
agcagggagctagaacgattcgcagttaatcctggcctgttagaaacatcagaaggctgtagacaaatactgggacagctacaaccatcccttcagacaggatcagaagaa
cttagatcattatataatacagtagcaaccctctattgtgtgcatcaaaggatagagataaaagacaccaaggaagctttagacaagatagaggaagagcaaaacaaaagta
agaccaccgcacagcaagcggccggccgctgatcttcagacctggaggaggagatatgagggacaattggagaagtgaattatataaatataaagtagtaaaaattgaac
cattaggagtagcacccaccaaggcaaagagaagagtggtgcagagagaaaaaagagcagtgggaataggagctttgttccttgggttcttgggagcagcaggaagcact
atgggcgcagcgtcaatgacgctgacggtacaggccagacaattattgtctggtatagtgcagcagcagaacaatttgctgagggctattgaggcgcaacagcatctgttgca
actcacagtctggggcatcaagcagctccaggcaagaatcctggctgtggaaagatacctaaaggatcaacagctcctggggatttggggttgctctggaaaactcatttgcac
cactgctgtgccttggaatgctagttggagtaataaatctctggaacagatttggaatcacacgacctggatggagtgggacagagaaattaacaattacacaagcttaatacac
tccttaattgaagaatcgcaaaaccagcaagaaaagaatgaacaagaattattggaattagataaatgggcaagtttgtggaattggtttaacataacaaattggctgtggtatat
aaaattattcataatgatagtaggaggcttggtaggtttaagaatagtttttgctgtactttctatagtgaatagagttaggcagggatattcaccattatcgtttcagacccacctccca
accccgaggggacccgacaggcccgaaggaatagaagaagaaggtggagagagagacagagacagatccattcgattagtgaacggatctcgacggtatcgccgaatt
cacaaatggcagtattcatccacaattttaaaagaaaaggggggattggggggtacagtgcaggggaaagaatagtagacataatagcaacagacatacaaactaaaga
attacaaaaacaaattacaaaaattcaaaattttcgggtttattacagggacagcagagatccagtttggactagtcgtgaggctccggtgcccgtcagtgggcagagcgcaca
tcgcccacagtccccgagaagttggggggaggggtcggcaattgaaccggtgcctagagaaggtggcgcggggtaaactgggaaagtgatgtcgtgtactggctccgccttt
ttcccgagggtgggggagaaccgtatataagtgcagtagtcgccgtgaacgttctttttcgcaacgggtttgccgccagaacacaggtaagtgccgtgtgtggttcccgcgggcc
tggcctctttacgggttatggcccttgcgtgccttgaattacttccacctggctgcagtacgtgattcttgatcccgagcttcgggttggaagtgggtgggagagttcgaggccttgcg
cttaaggagccccttcgcctcgtgcttgagttgaggcctggcctgggcgctggggccgccgcgtgcgaatctggtggcaccttcgcgcctgtctcgctgctttcgataagtctctag
ccatttaaaatttttgatgacctgctgcgacgctttttttctggcaagatagtcttgtaaatgcgggccaagatctgcacactggtatttcggtttttggggccgcgggcggcgacggg
gcccgtgcgtcccagcgcacatgttcggcgaggcggggcctgcgagcgcggccaccgagaatcggacgggggtagtctcaagctggccggcctgctctggtgcctggcctc
gcgccgccgtgtatcgccccgccctgggcggcaaggctggcccggtcggcaccagttgcgtgagcggaaagatggccgcttcccggccctgctgcagggagctcaaaatg
gaggacgcggcgctcgggagagcgggcgggtgagtcacccacacaaaggaaaagggcctttccgtcctcagccgtcgcttcatgtgactccacggagtaccgggcgccgt
ccaggcacctcgattagttctcgagcttttggagtacgtcgtctttaggttggggggaggggttttatgcgatggagtttccccacactgagtgggtggagactgaagttaggccag
cttggcacttgatgtaattctccttggaatttgccctttttgagtttggatcttggttcattctcaagcctcagacagtggttcaaagtttttttcttccatttcaggtgtcgtgaagcggccgcc
accatggtgagcaagggcgaggagctgttcaccggggtggtgcccatcctggtcgagctggacggcgacgtaaacggccacaagttcagcgtgtccggcgagggcgagg
gcgatgccacctacggcaagctgacCCtgaagttcatctgcaccaccggcaagctgcccgtgccctggcccacTTtGgtgaccaccctgacctAcggcgtgcagtgcttca
gccgctaccccgaccacatgaagcagcacgacttcttcaagtccgccatgcccgaaggctacgtccaggagcgcaccatcttcttcaaggacgacggcaactacaagaccc
gcgccgaggtgaagttcgagggcgacaccctggtgaaccgcatcgagctgaagggcatcgacttcaaggaggacggcaacatcctggggcacaagctggagtacaacta
caacagccacaacgtctatatcatggccgacaagcagaagaacggcatcaaggtgaacttcaagatccgccacaacatcgaggacggcagcgtgcagctcgccgacca
ctaccagcagaacacccccatcggcgacggccccgtgctgctgcccgacaaccactacctgagcacccagtccgccctgagcaaagaccccaacgagaagcgcgatca
catggtcctgctggagttcgtgaccgccgccgggatcactctcggcatggacgagctgtacaagtaaggatccctcccccccccctaacgttactggccgaagccgcttggaat
aaggccggtgtgcgtttgtctatatgttattttccaccatattgccgtcttttggcaatgtgagggcccggaaacctggccctgtcttcttgacgagcattcctaggggtctttcccctctc
gccaaaggaatgcaaggtctgttgaatgtcgtgaaggaagcagttcctctggaagcttcttgaagacaaacaacgtctgtagcgaccctttgcaggcagcggaaccccccac
ctggcgacaggtgcctctgcggccaaaagccacgtgtataagatacacctgcaaaggcggcacaaccccagtgccacgttgtgagttggatagttgtggaaagagtcaaatg
gctctcctcaagcgtattcaacaaggggctgaaggatgcccagaaggtaccccattgtatgggatctgatctggggcctcggtgcacatgctttacatgtgtttagtcgaggttaaa
aaaacgtctaggccccccgaaccacggggacgtggttttcctttgaaaaacacgatgataatatggccacacatatgaccgagtacaagcccacggtgcgcctcgccacccg
cgacgacgtccccagggccgtacgcaccctcgccgccgcgttcgccgactaccccgccacgcgccacaccgtcgatccggaccgccacatcgagcgggtcaccgagctg
caagaactcttcctcacgcgcgtcgggctcgacatcggcaaggtgtgggtcgcggacgacggcgccgcggtggcggtctggaccacgccggagagcgtcgaagcggggg
cggtgttcgccgagatcggcccgcgcatggccgagttgagcggttcccggctggccgcgcagcaacagatggaaggcctcctggcgccgcaccggcccaaggagcccgc
gtggttcctggccaccgtcggcgtctcgcccgaccaccagggcaagggtctgggcagcgccgtcgtgctccccggagtggaggcggccgagcgcgccggggtgcccgcctt
cctggagacctccgcgccccgcaacctccccttctacgagcggctcggcttcaccgtcaccgccgacgtcgaggtgcccgaaggaccgcgcacctggtgcatgacccgcaa
gcccggtgcctgaatcgatagatcctaatcaacctctggattacaaaatttgtgaaagattgactggtattcttaactatgttgctccttttacgctatgtggatacgctgctttaatgcctt
tgtatcatgctattgcttcccgtatggctttcattttctcctccttgtataaatcctggttgctgtctctttatgaggagttgtggcccgttgtcaggcaacgtggcgtggtgtgcactgtgtttg
ctgacgcaacccccactggttggggcattgccaccacctgtcagctcctttccgggactttcgctttccccctccctattgccacggcggaactcatcgccgcctgccttgcccgct
gctggacaggggctcggctgttgggcactgacaattccgtggtgttgtcggggaaatcatcgtcctttccttggctgctcgcctgtgttgccacctggattctgcgcgggacgtccttc
tgctacgtcccttcggccctcaatccagcggaccttccttcccgcggcctgctgccggctctgcggcctcttccgcgtcttcgccttcgccctcagacgagtcggatctccctttgggc
cgcctccccgcctggtacctttaagaccaatgacttacaaggcagctgtagatcttagccactttttaaaagaaaaggggggactggaagggctaattcactcccaacgaaga
caagatcacctgcaggacaggcgcgccctgctttttgcttgtactgggtctctctggttagaccagatctgagcctgggagctctctggctaactagggaacccactgcttaagcct
caataaagcttgccttgagtgcttcaagtagtgtgtgcccgtctgttgtgtgactctggtaactagagatccctcagacccttttagtcagtgtggaaaatctctagcacccgggcgat
taaggaaagggctagatcattcttgaagacgaaagggcctcgtgatacgcctatttttataggttaatgtcatgataataatggtttcttagacgtcaggtggcacttttcggggaaat

gtgcgcggaacccctatttgtttatttttctaaatacattcaaatatgtatccgctcatgagacaataaccctgataaatgcttcaataatattgaaaaaggaagagtatgagtattca
acatttccgtgtcgcccttattcccttttttgcggcattttgccttcctgtttttgctcacccagaaacgctggtgaaagtaaaagatgctgaagatcagttgggtgcacgagtgggttac
atcgaactggatctcaacagcggtaagatccttgagagttttcgccccgaagaacgttttccaatgatgagcacttttaaagttctgctatgtggcgcggtattatcccgtgttgacgc
cgggcaagagcaactcggtcgccgcatacactattctcagaatgacttggttgagtactcaccagtcacagaaaagcatcttacggatggcatgacagtaagagaattatgca
gtgctgccataaccatgagtgataacactgcggccaacttacttctgacaacgatcggaggaccgaaggagctaaccgcttttttgcacaacatgggggatcatgtaactcgcc
ttgatcgttgggaaccggagctgaatgaagccataccaaacgacgagcgtgacaccacgatgcctgtagcaatggcaacaacgttgcgcaaactattaactggcgaactact
tactctagcttcccggcaacaattaatagactggatggaggcggataaagttgcaggaccacttctgcgctcggcccttccggctggctggtttattgctgataaatctggagccg
gtgagcgtgggtctcgcggtatcattgcagcactggggccagatggtaagccctcccgtatcgtagttatctacacgacggggagtcaggcaactatggatgaacgaaataga
cagatcgctgagataggtgcctcactgattaagcattggtaactgtcagaccaagtttactcatatatactttagattgatttaaaacttcatttttaatttaaaaggatctaggtgaaga
tcctttttgataatctcatgaccaaaatcccttaacgtgagttttcgttccactgagcgtcagaccccgtagaaaagatcaaaggatcttcttgagatcctttttttctgcgcgtaatctgc
tgcttgcaaacaaaaaaaccaccgctaccagcggtggtttgtttgccggatcaagagctaccaactctttttccgaaggtaactggcttcagcagagcgcagataccaaatact
gttcttctagtgtagccgtagttaggccaccacttcaagaactctgtagcaccgcctacatacctcgctctgctaatcctgttaccagtggctgctgccagtggcgataagtcgtgtct
taccgggttggactcaagacgatagttaccggataaggcgcagcggtcgggctgaacggggggttcgtgcacacagcccagcttggagcgaacgacctacaccgaactga
gatacctacagcgtgagctatgagaaagcgccacgcttcccgaagggagaaaggcggacaggtatccggtaagcggcagggtcggaacaggagagcgcacgaggga
gcttccagggggaaacgcctggtatctttatagtcctgtcgggtttcgccacctctgacttgagcgtcgatttttgtgatgctcgtcaggggggcggagcctatggaaaaacgccag
caacgcggcctttttacggttcctggccttttgctggccttttgctcacatgttctttcctgcgttatcccctgattctgtggataaccgtattaccgcctttgagtgagctgataccgctcgc
cgcagccgaacgaccgagcgcagcgagtcagtgagcgaggaagcggaagagcgcccaatacgcaaaccgcctctccccgcgcgttggccgattcattaatgcagcaag
ctcatggctgactaattttttttatttatgcagaggccgaggccgcctcggcctctgagctattccagaagtagtgaggaggcttttttggaggcctaggcttttgcaaaaagctcccc
gtggcacgacaggtttcccgactggaaagcgggcagtgagcgcaacgcaattaatgtgagttagctcactcattaggcaccccaggctttacactttatgcttccggctcgtatgt
tgtgtggaattgtgagcggataacaatttcacacaggaaacagctatgacatgattacgaatttcacaaataaagcatttttttcactgcattctagttgtggtttgtccaaactcatca
atgtatcttatcatgtctggatcaactggataactcaagctaaccaaaatcatcccaaacttcccaccccataccctattaccactgccaattacctgtggtttcatttactctaaacct
gtgattcctctgaattattttcattttaaagaaattgtatttgttaaatatgtactacaaacttagtagt

Name: pEF-1a_dEGFP-deltaFluor-IRES-PuroR-WPRE (Addgene ID = 251212)

Map:

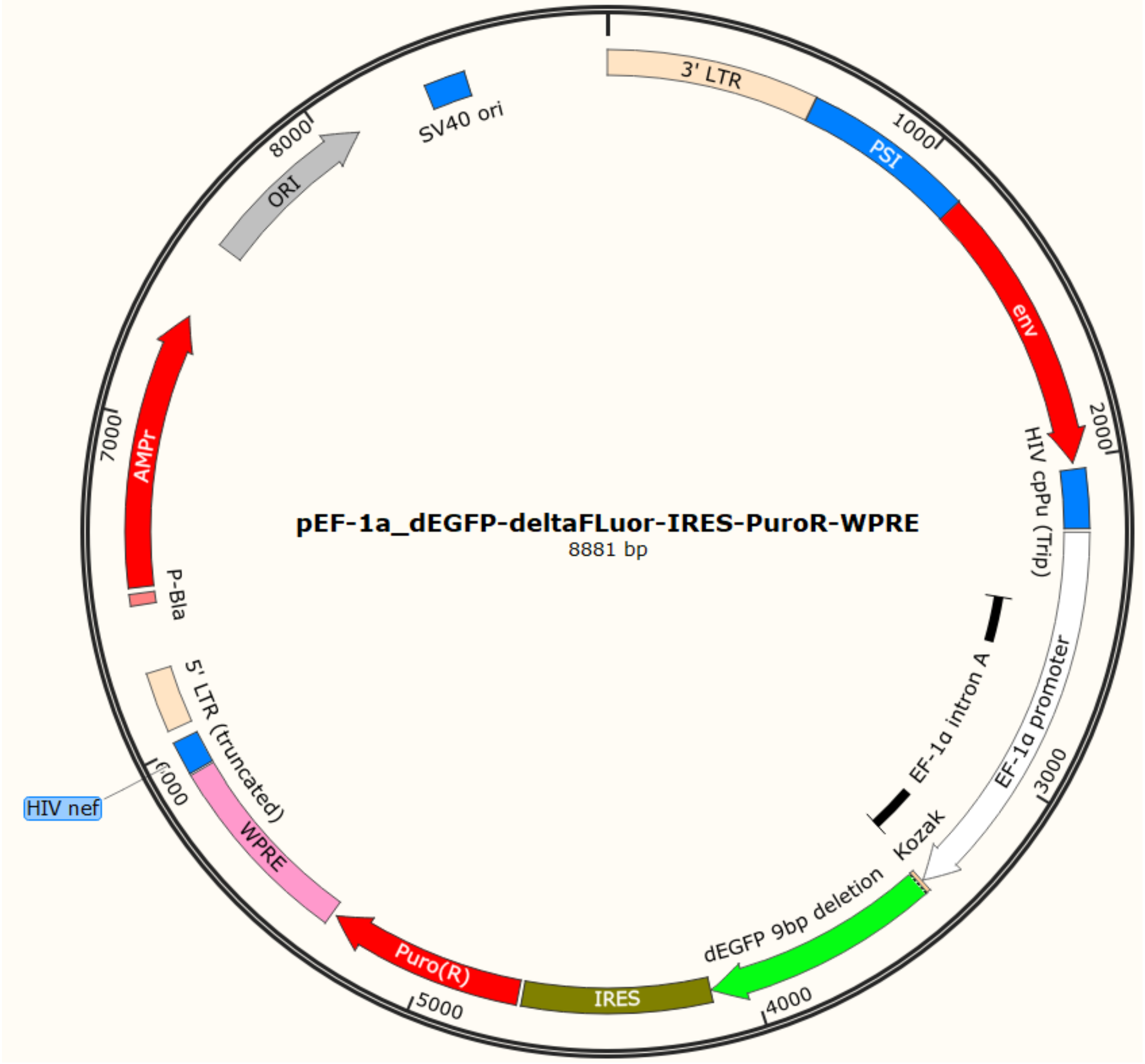

Sequence:

tggaagggctaattcactcccaaagaagacaagatatccttgatctgtggatctaccacacacaaggctacttccctgattagcagaactacacaccagggccaggggtcag
atatccactgacctttggatggtgctacaagctagtaccagttgagccagataaggtagaagaggccaataaaggagagaacaccagcttgttacaccctgtgagcctgcatg
ggatggatgacccggagagagaagtgttagagtggaggtttgacagccgcctagcatttcatcacgtggcccgagagctgcatccggagtacttcaagaactgctgatatcga
gcttgctacaagggactttccgctggggactttccagggaggcgtggcctgggcgggactggggagtggcgagccctcagatcctgcatataagcagctgctttttgcctgtact
gggtctctctggttagaccagatctgagcctgggagctctctggctaactagggaacccactgcttaagcctcaataaagcttgccttgagtgcttcaagtagtgtgtgcccgtctgt
tgtgtgactctggtaactagagatccctcagacccttttagtcagtgtggaaaatctctagcagtggcgcccgaacagggacttgaaagcgaaagggaaaccagaggagctct
ctcgacgcaggactcggcttgctgaagcgcgcacggcaagaggcgaggggcggcgactggtgagtacgccaaaaattttgactagcggaggctagaaggagagagatg
ggtgcgagagcgtcagtattaagcgggggagaattagatcgcgatgggaaaaaattcggttaaggccagggggaaagaaaaaatataaattaaaacatatagtatgggca
agcagggagctagaacgattcgcagttaatcctggcctgttagaaacatcagaaggctgtagacaaatactgggacagctacaaccatcccttcagacaggatcagaagaa
cttagatcattatataatacagtagcaaccctctattgtgtgcatcaaaggatagagataaaagacaccaaggaagctttagacaagatagaggaagagcaaaacaaaagta
agaccaccgcacagcaagcggccggccgctgatcttcagacctggaggaggagatatgagggacaattggagaagtgaattatataaatataaagtagtaaaaattgaac
cattaggagtagcacccaccaaggcaaagagaagagtggtgcagagagaaaaaagagcagtgggaataggagctttgttccttgggttcttgggagcagcaggaagcact
atgggcgcagcgtcaatgacgctgacggtacaggccagacaattattgtctggtatagtgcagcagcagaacaatttgctgagggctattgaggcgcaacagcatctgttgca
actcacagtctggggcatcaagcagctccaggcaagaatcctggctgtggaaagatacctaaaggatcaacagctcctggggatttggggttgctctggaaaactcatttgcac
cactgctgtgccttggaatgctagttggagtaataaatctctggaacagatttggaatcacacgacctggatggagtgggacagagaaattaacaattacacaagcttaatacac
tccttaattgaagaatcgcaaaaccagcaagaaaagaatgaacaagaattattggaattagataaatgggcaagtttgtggaattggtttaacataacaaattggctgtggtatat
aaaattattcataatgatagtaggaggcttggtaggtttaagaatagtttttgctgtactttctatagtgaatagagttaggcagggatattcaccattatcgtttcagacccacctccca
accccgaggggacccgacaggcccgaaggaatagaagaagaaggtggagagagagacagagacagatccattcgattagtgaacggatctcgacggtatcgccgaatt

cacaaatggcagtattcatccacaattttaaaagaaaaggggggattggggggtacagtgcaggggaaagaatagtagacataatagcaacagacatacaaactaaaga
attacaaaaacaaattacaaaaattcaaaattttcgggtttattacagggacagcagagatccagtttggactagtcgtgaggctccggtgcccgtcagtgggcagagcgcaca
tcgcccacagtccccgagaagttggggggaggggtcggcaattgaaccggtgcctagagaaggtggcgcggggtaaactgggaaagtgatgtcgtgtactggctccgccttt
ttcccgagggtgggggagaaccgtatataagtgcagtagtcgccgtgaacgttctttttcgcaacgggtttgccgccagaacacaggtaagtgccgtgtgtggttcccgcgggcc
tggcctctttacgggttatggcccttgcgtgccttgaattacttccacctggctgcagtacgtgattcttgatcccgagcttcgggttggaagtgggtgggagagttcgaggccttgcg
cttaaggagccccttcgcctcgtgcttgagttgaggcctggcctgggcgctggggccgccgcgtgcgaatctggtggcaccttcgcgcctgtctcgctgctttcgataagtctctag
ccatttaaaatttttgatgacctgctgcgacgctttttttctggcaagatagtcttgtaaatgcgggccaagatctgcacactggtatttcggtttttggggccgcgggcggcgacggg
gcccgtgcgtcccagcgcacatgttcggcgaggcggggcctgcgagcgcggccaccgagaatcggacgggggtagtctcaagctggccggcctgctctggtgcctggcctc
gcgccgccgtgtatcgccccgccctgggcggcaaggctggcccggtcggcaccagttgcgtgagcggaaagatggccgcttcccggccctgctgcagggagctcaaaatg
gaggacgcggcgctcgggagagcgggcgggtgagtcacccacacaaaggaaaagggcctttccgtcctcagccgtcgcttcatgtgactccacggagtaccgggcgccgt
ccaggcacctcgattagttctcgagcttttggagtacgtcgtctttaggttggggggaggggttttatgcgatggagtttccccacactgagtgggtggagactgaagttaggccag
cttggcacttgatgtaattctccttggaatttgccctttttgagtttggatcttggttcattctcaagcctcagacagtggttcaaagtttttttcttccatttcaggtgtcgtgaagcggccgcc
accatggtgagcaagggcgaggagctgttcaccggggtggtgcccatcctggtcgagctggacggcgacgtaaacggccacaagttcagcgtgtccggcgagggcgagg
gcgatgccacctacggcaagctgaccctgaaAttcatctgcaccaccggcaagctgcccgtgccctggcccaccctcgtgaccaccctggtgcagtgTttcagccgctaccc
cgaccacatgaagcagcacgacttcttcaagtccgccatgcccgaaggctacgtccaggagcgcaccatcttcttcaaggacgacggcaactacaagacccgcgccgagg
tgaagttcgagggcgacaccctggtgaaccgcatcgagctgaagggcatcgacttcaaggaggacggcaacatcctggggcacaagctggagtacaactacaacagcca
caacgtctatatcatggccgacaagcagaagaacggcatcaaggtgaacttcaagatccgccacaacatcgaggacggcagcgtgcagctcgccgaccactaccagcag
aacacccccatcggcgacggccccgtgctgctgcccgacaaccactacctgagcacccagtccgccctgagcaaagaccccaacgagaagcgcgatcacatggtcctgct
ggagttcgtgaccgccgccgggatcactctcggcatggacgagctgtacaagtaaggatccctcccccccccctaacgttactggccgaagccgcttggaataaggccggtgt
gcgtttgtctatatgttattttccaccatattgccgtcttttggcaatgtgagggcccggaaacctggccctgtcttcttgacgagcattcctaggggtctttcccctctcgccaaaggaat
gcaaggtctgttgaatgtcgtgaaggaagcagttcctctggaagcttcttgaagacaaacaacgtctgtagcgaccctttgcaggcagcggaaccccccacctggcgacaggt
gcctctgcggccaaaagccacgtgtataagatacacctgcaaaggcggcacaaccccagtgccacgttgtgagttggatagttgtggaaagagtcaaatggctctcctcaag
cgtattcaacaaggggctgaaggatgcccagaaggtaccccattgtatgggatctgatctggggcctcggtgcacatgctttacatgtgtttagtcgaggttaaaaaaacgtctag
gccccccgaaccacggggacgtggttttcctttgaaaaacacgatgataatatggccacacatatgaccgagtacaagcccacggtgcgcctcgccacccgcgacgacgtc
cccagggccgtacgcaccctcgccgccgcgttcgccgactaccccgccacgcgccacaccgtcgatccggaccgccacatcgagcgggtcaccgagctgcaagaactctt
cctcacgcgcgtcgggctcgacatcggcaaggtgtgggtcgcggacgacggcgccgcggtggcggtctggaccacgccggagagcgtcgaagcgggggcggtgttcgcc
gagatcggcccgcgcatggccgagttgagcggttcccggctggccgcgcagcaacagatggaaggcctcctggcgccgcaccggcccaaggagcccgcgtggttcctgg
ccaccgtcggcgtctcgcccgaccaccagggcaagggtctgggcagcgccgtcgtgctccccggagtggaggcggccgagcgcgccggggtgcccgccttcctggagac
ctccgcgccccgcaacctccccttctacgagcggctcggcttcaccgtcaccgccgacgtcgaggtgcccgaaggaccgcgcacctggtgcatgacccgcaagcccggtgc
ctgaatcgatagatcctaatcaacctctggattacaaaatttgtgaaagattgactggtattcttaactatgttgctccttttacgctatgtggatacgctgctttaatgcctttgtatcatgc
tattgcttcccgtatggctttcattttctcctccttgtataaatcctggttgctgtctctttatgaggagttgtggcccgttgtcaggcaacgtggcgtggtgtgcactgtgtttgctgacgcaa
cccccactggttggggcattgccaccacctgtcagctcctttccgggactttcgctttccccctccctattgccacggcggaactcatcgccgcctgccttgcccgctgctggacag
gggctcggctgttgggcactgacaattccgtggtgttgtcggggaaatcatcgtcctttccttggctgctcgcctgtgttgccacctggattctgcgcgggacgtccttctgctacgtcc
cttcggccctcaatccagcggaccttccttcccgcggcctgctgccggctctgcggcctcttccgcgtcttcgccttcgccctcagacgagtcggatctccctttgggccgcctcccc
gcctggtacctttaagaccaatgacttacaaggcagctgtagatcttagccactttttaaaagaaaaggggggactggaagggctaattcactcccaacgaagacaagatcac
ctgcaggacaggcgcgccctgctttttgcttgtactgggtctctctggttagaccagatctgagcctgggagctctctggctaactagggaacccactgcttaagcctcaataaagc
ttgccttgagtgcttcaagtagtgtgtgcccgtctgttgtgtgactctggtaactagagatccctcagacccttttagtcagtgtggaaaatctctagcacccgggcgattaaggaaag
ggctagatcattcttgaagacgaaagggcctcgtgatacgcctatttttataggttaatgtcatgataataatggtttcttagacgtcaggtggcacttttcggggaaatgtgcgcgga
acccctatttgtttatttttctaaatacattcaaatatgtatccgctcatgagacaataaccctgataaatgcttcaataatattgaaaaaggaagagtatgagtattcaacatttccgtgt
cgcccttattcccttttttgcggcattttgccttcctgtttttgctcacccagaaacgctggtgaaagtaaaagatgctgaagatcagttgggtgcacgagtgggttacatcgaactgga
tctcaacagcggtaagatccttgagagttttcgccccgaagaacgttttccaatgatgagcacttttaaagttctgctatgtggcgcggtattatcccgtgttgacgccgggcaagag
caactcggtcgccgcatacactattctcagaatgacttggttgagtactcaccagtcacagaaaagcatcttacggatggcatgacagtaagagaattatgcagtgctgccataa
ccatgagtgataacactgcggccaacttacttctgacaacgatcggaggaccgaaggagctaaccgcttttttgcacaacatgggggatcatgtaactcgccttgatcgttggga
accggagctgaatgaagccataccaaacgacgagcgtgacaccacgatgcctgtagcaatggcaacaacgttgcgcaaactattaactggcgaactacttactctagcttcc
cggcaacaattaatagactggatggaggcggataaagttgcaggaccacttctgcgctcggcccttccggctggctggtttattgctgataaatctggagccggtgagcgtgggt
ctcgcggtatcattgcagcactggggccagatggtaagccctcccgtatcgtagttatctacacgacggggagtcaggcaactatggatgaacgaaatagacagatcgctgag
ataggtgcctcactgattaagcattggtaactgtcagaccaagtttactcatatatactttagattgatttaaaacttcatttttaatttaaaaggatctaggtgaagatcctttttgataatc
tcatgaccaaaatcccttaacgtgagttttcgttccactgagcgtcagaccccgtagaaaagatcaaaggatcttcttgagatcctttttttctgcgcgtaatctgctgcttgcaaaca
aaaaaaccaccgctaccagcggtggtttgtttgccggatcaagagctaccaactctttttccgaaggtaactggcttcagcagagcgcagataccaaatactgttcttctagtgta
gccgtagttaggccaccacttcaagaactctgtagcaccgcctacatacctcgctctgctaatcctgttaccagtggctgctgccagtggcgataagtcgtgtcttaccgggttgga

ctcaagacgatagttaccggataaggcgcagcggtcgggctgaacggggggttcgtgcacacagcccagcttggagcgaacgacctacaccgaactgagatacctacagc
gtgagctatgagaaagcgccacgcttcccgaagggagaaaggcggacaggtatccggtaagcggcagggtcggaacaggagagcgcacgagggagcttccagggggg
aaacgcctggtatctttatagtcctgtcgggtttcgccacctctgacttgagcgtcgatttttgtgatgctcgtcaggggggcggagcctatggaaaaacgccagcaacgcggcctt
tttacggttcctggccttttgctggccttttgctcacatgttctttcctgcgttatcccctgattctgtggataaccgtattaccgcctttgagtgagctgataccgctcgccgcagccgaac
gaccgagcgcagcgagtcagtgagcgaggaagcggaagagcgcccaatacgcaaaccgcctctccccgcgcgttggccgattcattaatgcagcaagctcatggctgact
aatttttttatttatgcagaggccgaggccgcctcggcctctgagctattccagaagtagtgaggaggcttttttggaggcctaggcttttgcaaaaagctccccgtggcacgacag
gtttcccgactggaaagcgggcagtgagcgcaacgcaattaatgtgagttagctcactcattaggcaccccaggctttacactttatgcttccggctcgtatgttgtgtggaattgtg
agcggataacaatttcacacaggaaacagctatgacatgattacgaatttcacaaataaagcatttttttcactgcattctagttgtggtttgtccaaactcatcaatgtatcttatcatg
tctggatcaactggataactcaagctaaccaaaatcatcccaaacttcccaccccataccctattaccactgccaattacctgtggtttcatttactctaaacctgtgattcctctgaat
tattttcattttaaagaaattgtatttgttaaatatgtactacaaacttagtagt

Name: pET15b_LbuCas13a-6xHIS (Addgene ID = 251213)

Map:

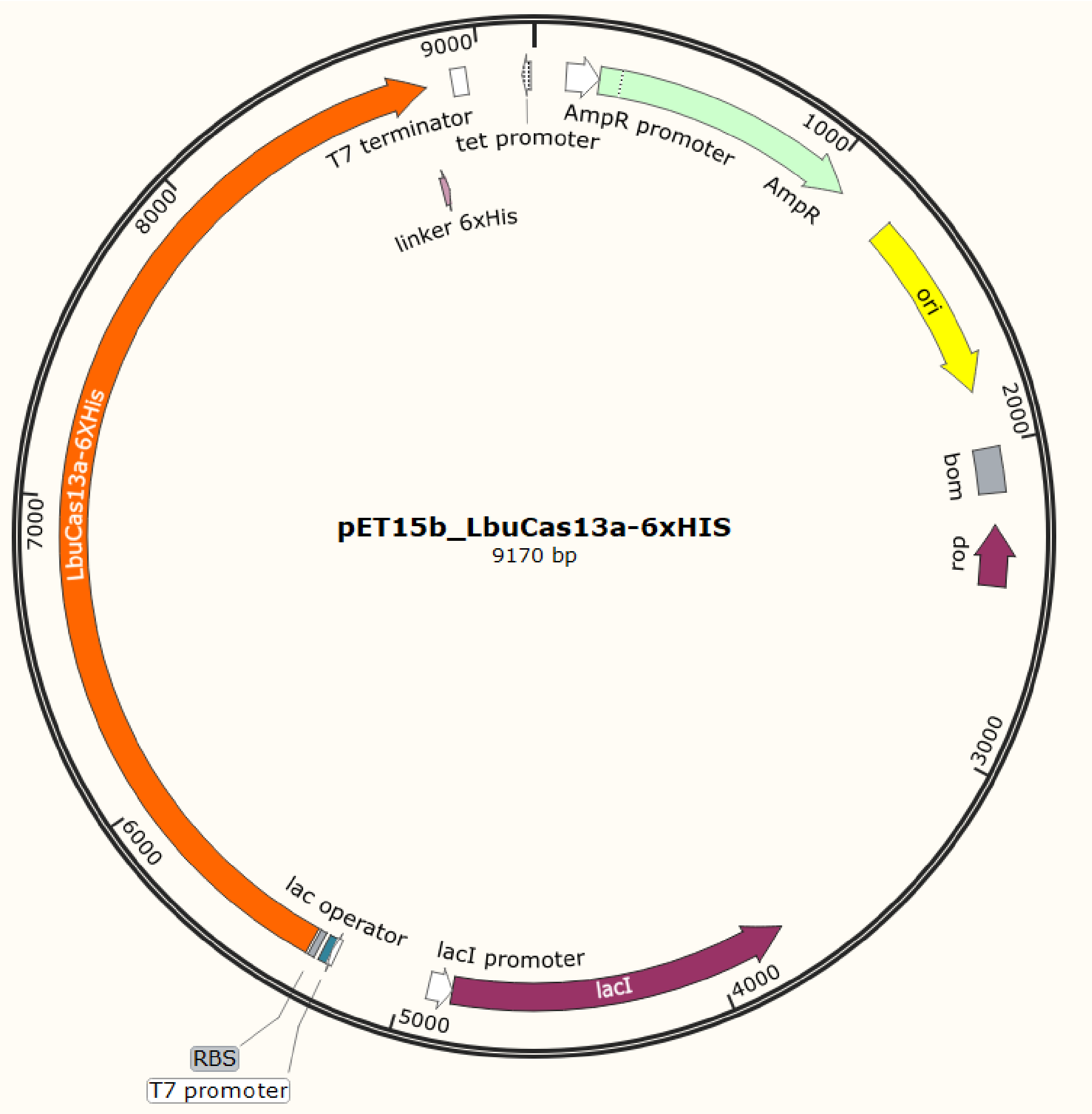

Sequence:
ttcttgaagacgaaagggcctcgtgatacgcctatttttataggttaatgtcatgataataatggtttcttagacgtcaggtggcacttttcggggaaatgtgcgcggaacccctatttgt
ttatttttctaaatacattcaaatatgtatccgctcatgagacaataaccctgataaatgcttcaataatattgaaaaaggaagagtatgagtattcaacatttccgtgtcgcccttattc
ccttttttgcggcattttgccttcctgtttttgctcacccagaaacgctggtgaaagtaaaagatgctgaagatcagttgggtgcacgagtgggttacatcgaactggatctcaacagc

ggtaagatccttgagagttttcgccccgaagaacgttttccaatgatgagcacttttaaagttctgctatgtggcgcggtattatcccgtgttgacgccgggcaagagcaactcggtc
gccgcatacactattctcagaatgacttggttgagtactcaccagtcacagaaaagcatcttacggatggcatgacagtaagagaattatgcagtgctgccataaccatgagtg
ataacactgcggccaacttacttctgacaacgatcggaggaccgaaggagctaaccgcttttttgcacaacatgggggatcatgtaactcgccttgatcgttgggaaccggagc
tgaatgaagccataccaaacgacgagcgtgacaccacgatgcctgcagcaatggcaacaacgttgcgcaaactattaactggcgaactacttactctagcttcccggcaaca
attaatagactggatggaggcggataaagttgcaggaccacttctgcgctcggcccttccggctggctggtttattgctgataaatctggagccggtgagcgtgggtctcgcggta
tcattgcagcactggggccagatggtaagccctcccgtatcgtagttatctacacgacggggagtcaggcaactatggatgaacgaaatagacagatcgctgagataggtgcc
tcactgattaagcattggtaactgtcagaccaagtttactcatatatactttagattgatttaaaacttcatttttaatttaaaaggatctaggtgaagatcctttttgataatctcatgacca
aaatcccttaacgtgagttttcgttccactgagcgtcagaccccgtagaaaagatcaaaggatcttcttgagatcctttttttctgcgcgtaatctgctgcttgcaaacaaaaaaacc
accgctaccagcggtggtttgtttgccggatcaagagctaccaactctttttccgaaggtaactggcttcagcagagcgcagataccaaatactgtccttctagtgtagccgtagtt
aggccaccacttcaagaactctgtagcaccgcctacatacctcgctctgctaatcctgttaccagtggctgctgccagtggcgataagtcgtgtcttaccgggttggactcaagac
gatagttaccggataaggcgcagcggtcgggctgaacggggggttcgtgcacacagcccagcttggagcgaacgacctacaccgaactgagatacctacagcgtgagcta
tgagaaagcgccacgcttcccgaagggagaaaggcggacaggtatccggtaagcggcagggtcggaacaggagagcgcacgagggagcttccagggggaaacgcct
ggtatctttatagtcctgtcgggtttcgccacctctgacttgagcgtcgatttttgtgatgctcgtcaggggggcggagcctatggaaaaacgccagcaacgcggcctttttacggttc
ctggccttttgctggccttttgctcacatgttctttcctgcgttatcccctgattctgtggataaccgtattaccgcctttgagtgagctgataccgctcgccgcagccgaacgaccgagc
gcagcgagtcagtgagcgaggaagcggaagagcgcctgatgcggtattttctccttacgcatctgtgcggtatttcacaccgcatatatggtgcactctcagtacaatctgctctg
atgccgcatagttaagccagtatacactccgctatcgctacgtgactgggtcatggctgcgccccgacacccgccaacacccgctgacgcgccctgacgggcttgtctgctccc
ggcatccgcttacagacaagctgtgaccgtctccgggagctgcatgtgtcagaggttttcaccgtcatcaccgaaacgcgcgaggcagctgcggtaaagctcatcagcgtggt
cgtgaagcgattcacagatgtctgcctgttcatccgcgtccagctcgttgagtttctccagaagcgttaatgtctggcttctgataaagcgggccatgttaagggcggttttttcctgttt
ggtcactgatgcctccgtgtaagggggatttctgttcatgggggtaatgataccgatgaaacgagagaggatgctcacgatacgggttactgatgatgaacatgcccggttactg
gaacgttgtgagggtaaacaactggcggtatggatgcggcgggaccagagaaaaatcactcagggtcaatgccagcgcttcgttaatacagatgtaggtgttccacagggta
gccagcagcatcctgcgatgcagatccggaacataatggtgcagggcgctgacttccgcgtttccagactttacgaaacacggaaaccgaagaccattcatgttgttgctcagg
tcgcagacgttttgcagcagcagtcgcttcacgttcgctcgcgtatcggtgattcattctgctaaccagtaaggcaaccccgccagcctagccgggtcctcaacgacaggagca
cgatcatgcgcacccgtggccaggacccaacgctgcccgagatgcgccgcgtgcggctgctggagatggcggacgcgatggatatgttctgccaagggttggtttgcgcattc
acagttctccgcaagaattgattggctccaattcttggagtggtgaatccgttagcgaggtgccgccggcttccattcaggtcgaggtggcccggctccatgcaccgcgacgcaa
cgcggggaggcagacaaggtatagggcggcgcctacaatccatgccaacccgttccatgtgctcgccgaggcggcataaatcgccgtgacgatcagcggtccagtgatcg
aagttaggctggtaagagccgcgagcgatccttgaagctgtccctgatggtcgtcatctacctgcctggacagcatggcctgcaacgcgggcatcccgatgccgccggaagc
gagaagaatcataatggggaaggccatccagcctcgcgtcgcgaacgccagcaagacgtagcccagcgcgtcggccgccatgccggcgataatggcctgcttctcgccga
aacgtttggtggcgggaccagtgacgaaggcttgagcgagggcgtgcaagattccgaataccgcaagcgacaggccgatcatcgtcgcgctccagcgaaagcggtcctcg
ccgaaaatgacccagagcgctgccggcacctgtcctacgagttgcatgataaagaagacagtcataagtgcggcgacgatagtcatgccccgcgcccaccggaaggagct
gactgggttgaaggctctcaagggcatcggtcgagatcccggtgcctaatgagtgagctaacttacattaattgcgttgcgctcactgcccgctttccagtcgggaaacctgtcgt
gccagctgcattaatgaatcggccaacgcgcggggagaggcggtttgcgtattgggcgccagggtggtttttcttttcaccagtgagacgggcaacagctgattgcccttcaccg
cctggccctgagagagttgcagcaagcggtccacgctggtttgccccagcaggcgaaaatcctgtttgatggtggttaacggcgggatataacatgagctgtcttcggtatcgtc
gtatcccactaccgagatatccgcaccaacgcgcagcccggactcggtaatggcgcgcattgcgcccagcgccatctgatcgttggcaaccagcatcgcagtgggaacgat
gccctcattcagcatttgcatggtttgttgaaaaccggacatggcactccagtcgccttcccgttccgctatcggctgaatttgattgcgagtgagatatttatgccagccagccaga
cgcagacgcgccgagacagaacttaatgggcccgctaacagcgcgatttgctggtgacccaatgcgaccagatgctccacgcccagtcgcgtaccgtcttcatgggagaaa
ataatactgttgatgggtgtctggtcagagacatcaagaaataacgccggaacattagtgcaggcagcttccacagcaatggcatcctggtcatccagcggatagttaatgatc
agcccactgacgcgttgcgcgagaagattgtgcaccgccgctttacaggcttcgacgccgcttcgttctaccatcgacaccaccacgctggcacccagttgatcggcgcgaga
tttaatcgccgcgacaatttgcgacggcgcgtgcagggccagactggaggtggcaacgccaatcagcaacgactgtttgcccgccagttgttgtgccacgcggttgggaatgt
aattcagctccgccatcgccgcttccactttttcccgcgttttcgcagaaacgtggctggcctggttcaccacgcgggaaacggtctgataagagacaccggcatactctgcgac
atcgtataacgttactggtttcacattcaccaccctgaattgactctcttccgggcgctatcatgccataccgcgaaaggttttgcgccattcgatggtgtccgggatctcgacgctctc
ccttatgcgactcctgcattaggaagcagcccagtagtaggttgaggccgttgagcaccgccgccgcaaggaatggtgcatgcaaggagatggcgcccaacagtcccccgg
ccacggggcctgccaccatacccacgccgaaacaagcgctcatgagcccgaagtggcgagcccgatcttccccatcggtgatgtcggcgatataggcgccagcaaccgca
cctgtggcgccggtgatgccggccacgatgcgtccggcgtagaggatcgagatctcgatcccgcgaaattaatacgactcactataggggaattgtgagcggataacaattcc
cctctagaaataattttgtttaactttaagaaggagatataccatgaaagtgacgaaggtaggaggcatttcgcataagaagtacacgtccgaaggccgcttagtgaagtcaga
atcggaagaaaatcgcacagacgaacgtctgtcggcgttgcttaatatgcgccttgacatgtatatcaagaatcccagcagcacggaaaccaaggaaaatcaaaaacgcatt
gggaaattaaagaaattcttctcaaacaaaatggtctatcttaaagacaataccttgagtttgaagaatgggaaaaaggagaacattgatcgtgagtattctgagactgacatcct
tgagagcgatgtccgtgacaagaaaaacttcgccgtgttgaaaaagatctatctgaatgaaaacgtgaactcggaggaattggaagtttttcgtaacgacattaagaagaaact
gaacaaaatcaacagcctgaagtactcatttgaaaagaataaggcgaattatcaaaagattaatgagaataacatcgagaaggttgaaggtaagtcaaagcgtaacattattt
acgattattatcgtgagtcagcgaaacgtgacgcttatgtaagcaatgtgaaagaagcctttgataagctttacaaggaagaggacattgcaaaacttgttcttgaaattgagaac
cttacgaagttagagaaatacaagattcgcgagttctaccacgaaattattggacgtaagaatgacaaggaaaactttgcaaaaatcatctacgaagaaatccagaatgttaat

aacatgaaagagttgatcgagaaggtaccggacatgagtgagttgaaaaagagccaagtattttacaagtattacttagacaaagaagagttgaacgacaagaacatcaaa
tacgcgttttgtcatttcgtggaaatcgaaatgagtcagttgctgaagaactacgtatataagcgcttaagtaatatctcgaatgacaaaattaagcgtatctttgaataccagaactt
gaaaaaattgatcgaaaataagctgttaaacaaacttgacacgtacgtccgtaattgtggaaagtataattattatttgcaagacggcgaaattgccacttcagatttcatcgcccg
caaccgtcagaatgaagcgtttcttcgcaacatcattggggtgtcatctgtggcctacttttctcttcgcaacattcttgaaacggagaacgagaatgatattactgggcgtatgcgc
ggcaaaacagttaagaacaataaaggtgaagagaagtacgtgtccggagaagttgataagatctataatgaaaataagaagaacgaggttaaggagaacttaaaaatgtt
ctattcgtacgatttcaatatggacaacaagaatgaaatcgaagatttcttcgccaacatcgacgaggcgatttcttccatccgtcacggtattgtccacttcaacttggaattagaa
ggtaaggatatctttgcgttcaagaacattgcgccatccgaaatctcaaagaagatgtttcagaatgagattaacgagaaaaaactgaaattgaagatctttcgtcaactgaact
ctgccaacgtgttccgctatctcgaaaagtataaaattctgaattaccttaaacgtacacgcttcgagtttgtcaataaaaatatcccattcgtcccgtctttcaccaaattatattcgcg
cattgatgacctgaagaatagtcttgggatttactggaaaactccgaaaacaaacgacgacaataagactaaggagattattgatgcccaaatctatttgcttaaaaacatctatt
acggggagttcctgaattatttcatgtcgaacaatggtaatttctttgagatttctaaagaaatcatcgaattgaacaagaacgataaacgcaacttaaagactgggttttacaagct
gcaaaagtttgaagacatccaggagaagattccaaaggaatacttggcgaatatccagtccctgtacatgattaatgccggtaatcaggacgaagaagaaaaggacacttat
attgatttcattcaaaagatcttcttaaagggatttatgacgtatcttgctaataacggtcgtttaagtctgatttacatcggctcggatgaagaaacaaatacgtcattagcagaaaa
gaagcaagagtttgacaagttcttgaagaagtacgagcagaacaataatatcaagatcccctatgagatcaatgaattcctgcgtgagatcaaactgggaaacatcctgaagt
atactgagcgtttaaacatgttctaccttatcttaaagcttttgaatcacaaggagctgacaaatctgaagggtagtcttgaaaaatatcagtctgccaataaggaagaagcgttctc
tgaccaattggagttaattaacctgcttaaccttgacaacaaccgcgtgacggaagacttcgaattagaggccgacgagattggaaaatttcttgatttcaatggcaacaaagtta
aggataacaaggaactgaaaaagttcgatacaaacaagatctactttgacggcgagaacattatcaaacaccgtgccttctacaatattaagaaatatggcatgttaaacttact
ggagaaaattgccgacaaggctggatacaagatctcgatcgaagagctgaagaaatactccaataaaaagaatgagatcgagaagaaccataagatgcaggaaaatctg
caccgcaaatacgctcgtccccgtaaagacgagaagtttacagatgaggactatgaaagttacaagcaagctattgagaatattgaggagtacacccaccttaagaacaag
gtagaattcaatgagctgaatttactgcagggcctgttgctgcgcattttacatcgtttagtcggatatacctcaatttgggaacgcgatctgcgcttccgccttaaaggtgagttccca
gaaaaccaatacatcgaagagatcttcaactttgaaaataagaagaacgtgaagtacaaaggggggtcagattgtagagaaatacattaaattctacaaggaattacatcaaa
atgatgaagttaagatcaacaagtacagttccgcgaatatcaaggtgttgaagcaagaaaagaaggacctttatattcgaaattacatcgcccacttcaattatattcctcacgcc
gagatctcactgctggaagtccttgaaaatttgcgtaaattgctgtcctacgatcgcaaactgaaaaatgccgtaatgaaatcagtagttgatatccttaaggagtatggttttgtag
ccacattcaaaatcggggcggacaagaagatcggtattcagacactggagagcgaaaaaatcgtgcatcttaagaatcttaagaagaagaagttaatgactgaccgcaattc
cgaggaactttgcaaattggtgaagattatgtttgaatacaaaatggaagagaaaaagtctgaaaacggcggtggctcgcatcatcatcatcatcactaataacattggaagtg
gataacggatccggctgctaacaaagcccgaaaggaagctgagttggctgctgccaccgctgagcaataactagcataaccccttggggcctctaaacgggtcttgaggggt
ttttgctgaaaggaggaactatatccggatatcccgcaagaggcccggcagtaccggcataaccaagcctatgcctacagcatccagggtgacggtgccgaggatgacgat
gagcgcattgttagatttcatacacggtgcctgactgcgttagcaatttaactgtgataaactaccgcattaaagcttatcgatgataagctgtcaaacatgagaa

Name: pET15b_LwaCas13a-6xHIS (Addgene ID = 251214)

Map:

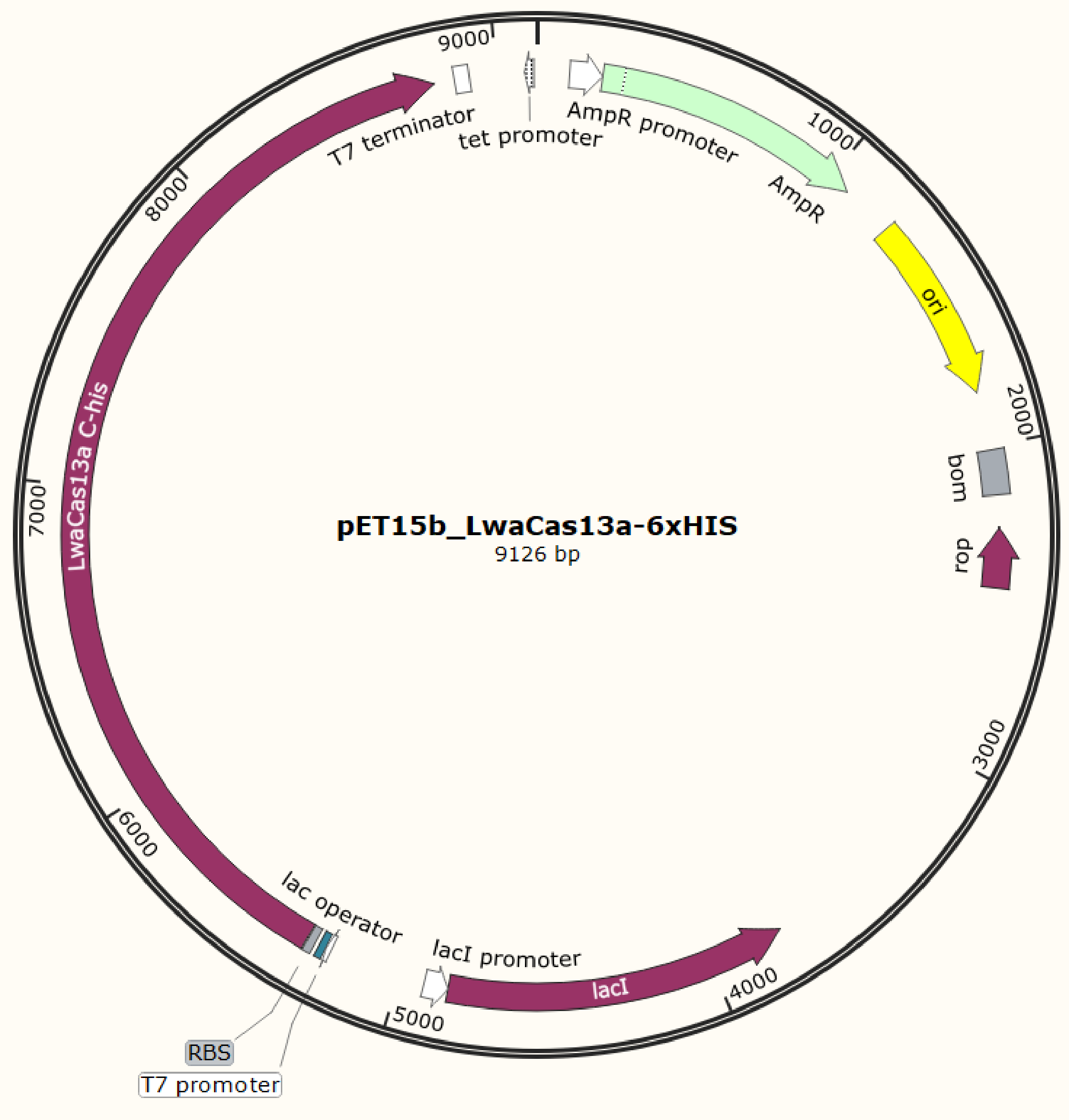

Sequence:

ttcttgaagacgaaagggcctcgtgatacgcctatttttataggttaatgtcatgataataatggtttcttagacgtcaggtggcacttttcggggaaatgtgcgcggaacccctatttgt
ttatttttctaaatacattcaaatatgtatccgctcatgagacaataaccctgataaatgcttcaataatattgaaaaaggaagagtatgagtattcaacatttccgtgtcgcccttattc
ccttttttgcggcattttgccttcctgtttttgctcacccagaaacgctggtgaaagtaaaagatgctgaagatcagttgggtgcacgagtgggttacatcgaactggatctcaacagc
ggtaagatccttgagagttttcgccccgaagaacgttttccaatgatgagcacttttaaagttctgctatgtggcgcggtattatcccgtgttgacgccgggcaagagcaactcggtc
gccgcatacactattctcagaatgacttggttgagtactcaccagtcacagaaaagcatcttacggatggcatgacagtaagagaattatgcagtgctgccataaccatgagtg
ataacactgcggccaacttacttctgacaacgatcggaggaccgaaggagctaaccgcttttttgcacaacatgggggatcatgtaactcgccttgatcgttgggaaccggagc
tgaatgaagccataccaaacgacgagcgtgacaccacgatgcctgcagcaatggcaacaacgttgcgcaaactattaactggcgaactacttactctagcttcccggcaaca
attaatagactggatggaggcggataaagttgcaggaccacttctgcgctcggcccttccggctggctggtttattgctgataaatctggagccggtgagcgtgggtctcgcggta
tcattgcagcactggggccagatggtaagccctcccgtatcgtagttatctacacgacggggagtcaggcaactatggatgaacgaaatagacagatcgctgagataggtgcc
tcactgattaagcattggtaactgtcagaccaagtttactcatatatactttagattgatttaaaacttcatttttaatttaaaaggatctaggtgaagatcctttttgataatctcatgacca
aaatcccttaacgtgagttttcgttccactgagcgtcagaccccgtagaaaagatcaaaggatcttcttgagatcctttttttctgcgcgtaatctgctgcttgcaaacaaaaaaacc
accgctaccagcggtggtttgtttgccggatcaagagctaccaactctttttccgaaggtaactggcttcagcagagcgcagataccaaatactgtccttctagtgtagccgtagtt
aggccaccacttcaagaactctgtagcaccgcctacatacctcgctctgctaatcctgttaccagtggctgctgccagtggcgataagtcgtgtcttaccgggttggactcaagac
gatagttaccggataaggcgcagcggtcgggctgaacggggggttcgtgcacacagcccagcttggagcgaacgacctacaccgaactgagatacctacagcgtgagcta
tgagaaagcgccacgcttcccgaagggagaaaggcggacaggtatccggtaagcggcagggtcggaacaggagagcgcacgagggagcttccagggggaaacgcct

ggtatctttatagtcctgtcgggtttcgccacctctgacttgagcgtcgatttttgtgatgctcgtcaggggggcggagcctatggaaaaacgccagcaacgcggcctttttacggttc
ctggccttttgctggccttttgctcacatgttctttcctgcgttatcccctgattctgtggataaccgtattaccgcctttgagtgagctgataccgctcgccgcagccgaacgaccgagc
gcagcgagtcagtgagcgaggaagcggaagagcgcctgatgcggtattttctccttacgcatctgtgcggtatttcacaccgcatatatggtgcactctcagtacaatctgctctg
atgccgcatagttaagccagtatacactccgctatcgctacgtgactgggtcatggctgcgccccgacacccgccaacacccgctgacgcgccctgacgggcttgtctgctccc
ggcatccgcttacagacaagctgtgaccgtctccgggagctgcatgtgtcagaggttttcaccgtcatcaccgaaacgcgcgaggcagctgcggtaaagctcatcagcgtggt
cgtgaagcgattcacagatgtctgcctgttcatccgcgtccagctcgttgagtttctccagaagcgttaatgtctggcttctgataaagcgggccatgttaagggcggtttttcctgttt
ggtcactgatgcctccgtgtaagggggatttctgttcatgggggtaatgataccgatgaaacgagagaggatgctcacgatacgggttactgatgatgaacatgcccggttactg
gaacgttgtgagggtaaacaactggcggtatggatgcggcgggaccagagaaaaatcactcagggtcaatgccagcgcttcgttaatacagatgtaggtgttccacagggta
gccagcagcatcctgcgatgcagatccggaacataatggtgcagggcgctgacttccgcgtttccagactttacgaaacacggaaaccgaagaccattcatgttgttgctcagg
tcgcagacgttttgcagcagcagtcgcttcacgttcgctcgcgtatcggtgattcattctgctaaccagtaaggcaaccccgccagcctagccgggtcctcaacgacaggagca
cgatcatgcgcacccgtggccaggacccaacgctgcccgagatgcgccgcgtgcggctgctggagatggcggacgcgatggatatgttctgccaagggttggtttgcgcattc
acagttctccgcaagaattgattggctccaattcttggagtggtgaatccgttagcgaggtgccgccggcttccattcaggtcgaggtggcccggctccatgcaccgcgacgcaa
cgcggggaggcagacaaggtatagggcggcgcctacaatccatgccaacccgttccatgtgctcgccgaggcggcataaatcgccgtgacgatcagcggtccagtgatcg
aagttaggctggtaagagccgcgagcgatccttgaagctgtccctgatggtcgtcatctacctgcctggacagcatggcctgcaacgcgggcatcccgatgccgccggaagc
gagaagaatcataatggggaaggccatccagcctcgcgtcgcgaacgccagcaagacgtagcccagcgcgtcggccgccatgccggcgataatggcctgcttctcgccga
aacgtttggtggcgggaccagtgacgaaggcttgagcgagggcgtgcaagattccgaataccgcaagcgacaggccgatcatcgtcgcgctccagcgaaagcggtcctcg
ccgaaaatgacccagagcgctgccggcacctgtcctacgagttgcatgataaagaagacagtcataagtgcggcgacgatagtcatgccccgcgcccaccggaaggagct
gactgggttgaaggctctcaagggcatcggtcgagatcccggtgcctaatgagtgagctaacttacattaattgcgttgcgctcactgcccgctttccagtcgggaaacctgtcgt
gccagctgcattaatgaatcggccaacgcgcggggagaggcggtttgcgtattgggcgccagggtggtttttcttttcaccagtgagacgggcaacagctgattgcccttcaccg
cctggccctgagagagttgcagcaagcggtccacgctggtttgccccagcaggcgaaaatcctgtttgatggtggttaacggcgggatataacatgagctgtcttcggtatcgtc
gtatcccactaccgagatatccgcaccaacgcgcagcccggactcggtaatggcgcgcattgcgcccagcgccatctgatcgttggcaaccagcatcgcagtgggaacgat
gccctcattcagcatttgcatggtttgttgaaaaccggacatggcactccagtcgccttcccgttccgctatcggctgaatttgattgcgagtgagatatttatgccagccagccaga
cgcagacgcgccgagacagaacttaatgggcccgctaacagcgcgatttgctggtgacccaatgcgaccagatgctccacgcccagtcgcgtaccgtcttcatgggagaaa
ataatactgttgatgggtgtctggtcagagacatcaagaaataacgccggaacattagtgcaggcagcttccacagcaatggcatcctggtcatccagcggatagttaatgatc
agcccactgacgcgttgcgcgagaagattgtgcaccgccgctttacaggcttcgacgccgcttcgttctaccatcgacaccaccacgctggcacccagttgatcggcgcgaga
tttaatcgccgcgacaatttgcgacggcgcgtgcagggccagactggaggtggcaacgccaatcagcaacgactgtttgcccgccagttgttgtgccacgcggttgggaatgt
aattcagctccgccatcgccgcttccactttttcccgcgttttcgcagaaacgtggctggcctggttcaccacgcgggaaacggtctgataagagacaccggcatactctgcgac
atcgtataacgttactggtttcacattcaccaccctgaattgactctcttccgggcgctatcatgccataccgcgaaaggttttgcgccattcgatggtgtccgggatctcgacgctctc
ccttatgcgactcctgcattaggaagcagcccagtagtaggttgaggccgttgagcaccgccgccgcaaggaatggtgcatgcaaggagatggcgcccaacagtcccccgg
ccacggggcctgccaccatacccacgccgaaacaagcgctcatgagcccgaagtggcgagcccgatcttccccatcggtgatgtcggcgatataggcgccagcaaccgca
cctgtggcgccggtgatgccggccacgatgcgtccggcgtagaggatcgagatctcgatcccgcgaaattaatacgactcactataggggaattgtgagcggataacaattcc
cctctagaaataattttgtttaactttaagaaggagatataccatgaaagtgaccaaggtcgacggcatcagccacaagaagtacatcgaagagggcaagctcgtgaagtcca
ccagcgaggaaaaccggaccagcgagagactgagcgagctgctgagcatccggctggacatctacatcaagaaccccgacaacgcctccgaggaagagaaccggatc
agaagagagaacctgaagaagttctttagcaacaaggtgctgcacctgaaggacagcgtgctgtatctgaagaaccggaaagaaaagaacgccgtgcaggacaagaact
atagcgaagaggacatcagcgagtacgacctgaaaaacaagaacagcttctccgtgctgaagaagatcctgctgaacgaggacgtgaactctgaggaactggaaatctttc
ggaaggacgtggaagccaagctgaacaagatcaacagcctgaagtacagcttcgaagagaacaaggccaactaccagaagatcaacgagaacaacgtggaaaaagt
gggcggcaagagcaagcggaacatcatctacgactactacagagagagcgccaagcgcaacgactacatcaacaacgtgcaggaagccttcgacaagctgtataagaa
agaggatatcgagaaactgtttttcctgatcgagaacagcaagaagcacgagaagtacaagatccgcgagtactatcacaagatcatcggccggaagaacgacaaagag
aacttcgccaagattatctacgaagagatccagaacgtgaacaacatcaaagagctgattgagaagatccccgacatgtctgagctgaagaaaagccaggtgttctacaagt
actacctggacaaagaggaactgaacgacaagaatattaagtacgccttctgccacttcgtggaaatcgagatgtcccagctgctgaaaaactacgtgtacaagcggctgag
caacatcagcaacgataagatcaagcggatcttcgagtaccagaatctgaaaaagctgatcgaaaacaaactgctgaacaagctggacacctacgtgcggaactgcggca
agtacaactactatctgcaagtgggcgagatcgccacctccgactttatcgcccggaaccggcagaacgaggccttcctgagaaacatcatcggcgtgtccagcgtggcctac
ttcagcctgaggaacatcctggaaaccgagaacgagaacggtatcaccggccggatgcggggcaagaccgtgaagaacaacaagggcgaagagaaatacgtgtccgg
cgaggtggacaagatctacaatgagaacaagcagaacgaagtgaaagaaaatctgaagatgttctacagctacgacttcaacatggacaacaagaacgagatcgagga
cttcttcgccaacatcgacgaggccatcagcagcatcagacacggcatcgtgcacttcaacctggaactggaaggcaaggacatcttcgccttcaagaatatcgcccccagc
gagatctccaagaagatgtttcagaacgaaatcaacgaaaagaagctgaagctgaaaatcttcaagcagctgaacagcgccaacgtgttcaactactacgagaaggatgtg
atcatcaagtacctgaagaataccaagttcaacttcgtgaacaaaaacatccccttcgtgcccagcttcaccaagctgtacaacaagattgaggacctgcggaataccctgaa
gtttttttggagcgtgcccaaggacaaagaagagaaggacgcccagatctacctgctgaagaatatctactacggcgagttcctgaacaagttcgtgaaaaactccaaggtgtt
ctttaagatcaccaatgaagtgatcaagattaacaagcagcggaaccagaaaaccggccactacaagtatcagaagttcgagaacatcgagaaaaccgtgcccgtggaat
acctggccatcatccagagcagagagatgatcaacaaccaggacaaagaggaaaagaatacctacatcgactttattcagcagattttcctgaagggcttcatcgactacctg

aacaagaacaatctgaagtatatcgagagcaacaacaacaatgacaacaacgacatcttctccaagatcaagatcaaaaaggataacaaagagaagtacgacaagatc<br>ctgaagaactatgagaagcacaatcggaacaaagaaatccctcacgagatcaatgagttcgtgcgcgagatcaagctggggaagattctgaagtacaccgagaatctgaa<br>catgttttacctgatcctgaagctgctgaaccacaaagagctgaccaacctgaagggcagcctggaaaagtaccagtccgccaacaaagaagaaaccttcagcgacgagtt<br>ggaactgatcaacctgctgaacctggacaacaacagagtgaccgaggacttcgagctggaagccaacgagatcggcaagttcctggacttcaacgaaaacaaaatcaag<br>gaccggaaagagctgaaaaagttcgacaccaacaagatctatttcgacggcgagaacatcatcaagcaccgggccttctacaatatcaagaaatacggcatgctgaatctg<br>ctggaaaagatcgccgataaggccaagtataagatcagcctgaaagaactgaaagagtacagcaacaagaagaatgagattgaaaagaactacaccatgcagcagaa<br>cctgcaccggaagtacgccagacccaagaaggacgaaaagttcaacgacgaggactacaaagagtatgagaaggccatcggcaacatccagaagtacacccacctga<br>agaacaaggtggaattcaatgagctgaacctgctgcagggcctgctgctgaagatcctgcaccggctcgtgggctacaccagcatctgggagcgggacctgagattccggct<br>gaagggcgagtttcccgagaaccactacatcgaggaaattttcaatttcgacaactccaagaatgtgaagtacaaaagcggccagatcgtggaaaagtatatcaacttctaca<br>aagaactgtacaaggacaatgtggaaaagcggagcatctactccgacaagaaagtgaagaaactgaagcaggaaaaaaaggacctgtacatccggaactacattgccc<br>acttcaactacatcccccacgccgagattagcctgctggaagtgctggaaaacctgcggaagctgctgtcctacgaccggaagctgaagaacgccatcatgaagtccatcgt<br>ggacattctgaaagaatacggcttcgtggccaccttcaagatcggcgctgacaagaagatcgaaatccagaccctggaatcagagaagatcgtgcacctgaagaatctgaa<br>gaaaaagaaactgatgaccgaccggaacagcgaggaactgtgcgaactcgtgaaagtcatgttcgagtacaaggccctggaaggcggtggctcgcatcatcatcatcatca<br>ctaaggatccggctgctaacaaagcccgaaaggaagctgagttggctgctgccaccgctgagcaataactagcataaccccttggggcctctaaacgggtcttgaggggtttt<br>tgctgaaaggaggaactatatccggatatcccgcaagaggcccggcagtaccggcataaccaagcctatgcctacagcatccagggtgacggtgccgaggatgacgatga<br>gcgcattgttagatttcatacacggtgcctgactgcgttagcaatttaactgtgataaactaccgcattaaagcttatcgatgataagctgtcaaacatgagaa
**Name**: pET15b_LshCas13a-6xHIS (Addgene ID = 251215)
**Map**:

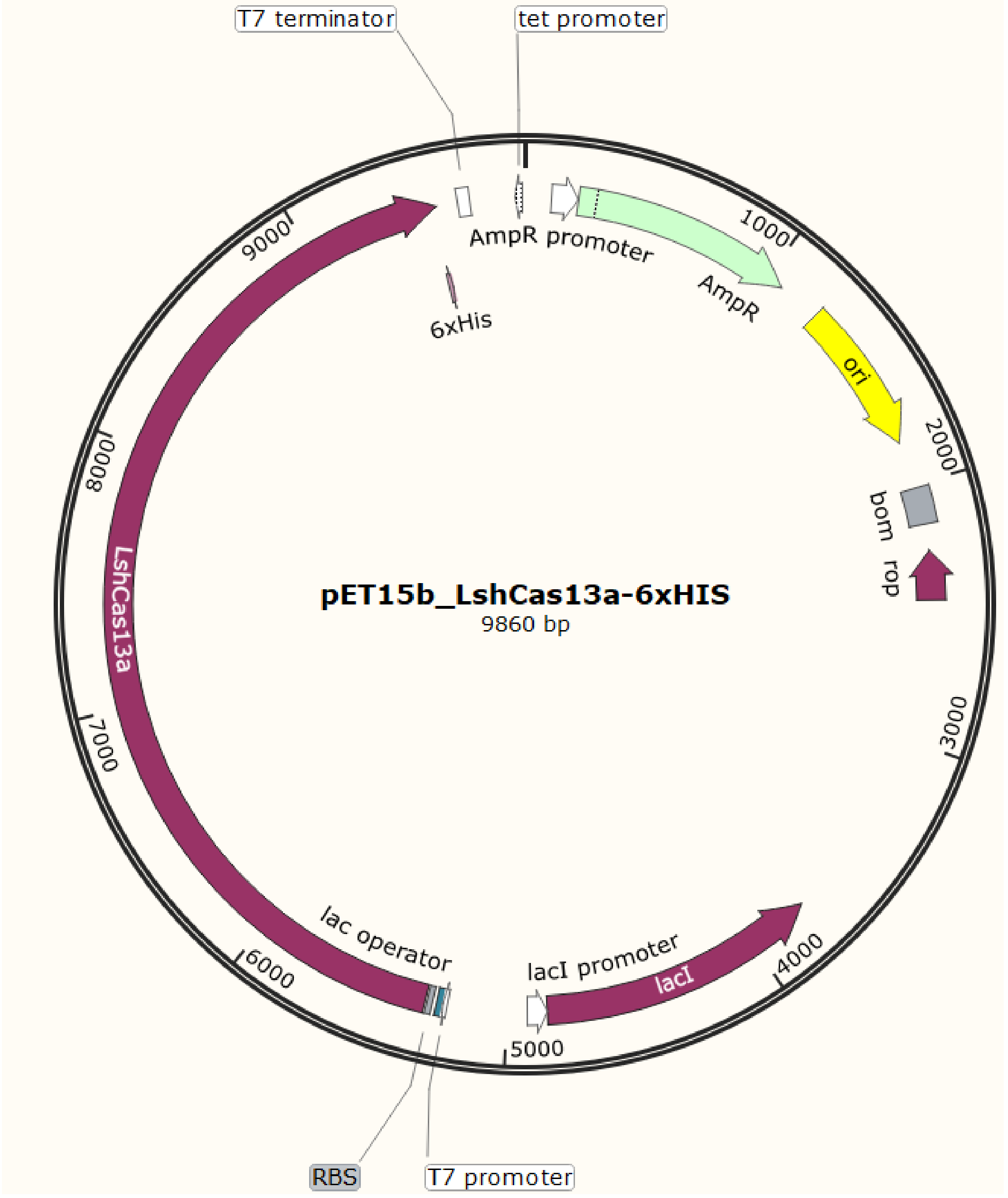

Sequence:

ttcttgaagacgaaagggcctcgtgatacgcctatttttataggttaatgtcatgataataatggtttcttagacgtcaggtggcacttttcggggaaatgtgcgcggaacccctatttgt
ttatttttctaaatacattcaaatatgtatccgctcatgagacaataaccctgataaatgcttcaataatattgaaaaaggaagagtatgagtattcaacatttccgtgtcgcccttattc
ccttttttgcggcattttgccttcctgtttttgctcacccagaaacgctggtgaaagtaaaagatgctgaagatcagttgggtgcacgagtgggttacatcgaactggatctcaacagc
ggtaagatccttgagagttttcgccccgaagaacgttttccaatgatgagcacttttaaagttctgctatgtggcgcggtattatcccgtgttgacgccgggcaagagcaactcggtc
gccgcatacactattctcagaatgacttggttgagtactcaccagtcacagaaaagcatcttacggatggcatgacagtaagagaattatgcagtgctgccataaccatgagtg
ataacactgcggccaacttacttctgacaacgatcggaggaccgaaggagctaaccgcttttttgcacaacatgggggatcatgtaactcgccttgatcgttgggaaccggagc
tgaatgaagccataccaaacgacgagcgtgacaccacgatgcctgcagcaatggcaacaacgttgcgcaaactattaactggcgaactacttactctagcttcccggcaaca
attaatagactggatggaggcggataaagttgcaggaccacttctgcgctcggcccttccggctggctggtttattgctgataaatctggagccggtgagcgtgggtctcgcggta
tcattgcagcactggggccagatggtaagccctcccgtatcgtagttatctacacgacggggagtcaggcaactatggatgaacgaaatagacagatcgctgagataggtgcc
tcactgattaagcattggtaactgtcagaccaagtttactcatatatactttagattgatttaaaacttcatttttaatttaaaaggatctaggtgaagatcctttttgataatctcatgacca
aaatcccttaacgtgagttttcgttccactgagcgtcagaccccgtagaaaagatcaaaggatcttcttgagatcctttttttctgcgcgtaatctgctgcttgcaaacaaaaaaacc
accgctaccagcggtggtttgtttgccggatcaagagctaccaactctttttccgaaggtaactggcttcagcagagcgcagataccaaatactgtccttctagtgtagccgtagtt
aggccaccacttcaagaactctgtagcaccgcctacatacctcgctctgctaatcctgttaccagtggctgctgccagtggcgataagtcgtgtcttaccgggttggactcaagac
gatagttaccggataaggcgcagcggtcgggctgaacggggggttcgtgcacacagcccagcttggagcgaacgacctacaccgaactgagatacctacagcgtgagcta

tgagaaagcgccacgcttcccgaagggagaaaggcggacaggtatccggtaagcggcagggtcggaacaggagagcgcacgagggagcttccaggggggaaacgcct
ggtatctttatagtcctgtcgggtttcgccacctctgacttgagcgtcgatttttgtgatgctcgtcaggggggcggagcctatggaaaaacgccagcaacgcggcctttttacggttc
ctggccttttgctggccttttgctcacatgttctttcctgcgttatcccctgattctgtggataaccgtattaccgcctttgagtgagctgataccgctcgccgcagccgaacgaccgagc
gcagcgagtcagtgagcgaggaagcggaagagcgcctgatgcggtattttctccttacgcatctgtgcggtatttcacaccgcatatatggtgcactctcagtacaatctgctctg
atgccgcatagttaagccagtatacactccgctatcgctacgtgactgggtcatggctgcgccccgacacccgccaacacccgctgacgcgccctgacgggcttgtctgctccc
ggcatccgcttacagacaagctgtgaccgtctccgggagctgcatgtgtcagaggttttcaccgtcatcaccgaaacgcgcgaggcagctgcggtaaagctcatcagcgtggt
cgtgaagcgattcacagatgtctgcctgttcatccgcgtccagctcgttgagtttctccagaagcgttaatgtctggcttctgataaagcgggccatgttaagggcggtttttttcctgttt
ggtcactgatgcctccgtgtaagggggatttctgttcatgggggtaatgataccgatgaaacgagagaggatgctcacgatacgggttactgatgatgaacatgcccggttactg
gaacgttgtgagggtaaacaactggcggtatggatgcggcgggaccagagaaaaatcactcagggtcaatgccagcgcttcgttaatacagatgtaggtgttccacagggta
gccagcagcatcctgcgatgcagatccggaacataatggtgcagggcgctgacttccgcgtttccagactttacgaaacacggaaaccgaagaccattcatgttgttgctcagg
tcgcagacgttttgcagcagcagtcgcttcacgttcgctcgcgtatcggtgattcattctgctaaccagtaaggcaaccccgccagcctagccgggtcctcaacgacaggagca
cgatcatgcgcacccgtggccaggacccaacgctgcccgagatgcgccgcgtgcggctgctggagatggcggacgcgatggatatgttctgccaagggttggtttgcgcattc
acagttctccgcaagaattgattggctccaattcttggagtggtgaatccgttagcgaggtgccgccggcttccattcaggtcgaggtggcccggctccatgcaccgcgacgcaa
cgcggggaggcagacaaggtatagggcggcgcctacaatccatgccaacccgttccatgtgctcgccgaggcggcataaatcgccgtgacgatcagcggtccagtgatcg
aagttaggctggtaagagccgcgagcgatccttgaagctgtccctgatggtcgtcatctacctgcctggacagcatggcctgcaacgcgggcatcccgatgccgccggaagc
gagaagaatcataatggggaaggccatccagcctcgcgtcgcgaacgccagcaagacgtagcccagcgcgtcggccgccatgccggcgataatggcctgcttctcgccga
aacgtttggtggcgggaccagtgacgaaggcttgagcgagggcgtgcaagattccgaataccgcaagcgacaggccgatcatcgtcgcgctccagcgaaagcggtcctcg
ccgaaaatgacccagagcgctgccggcacctgtcctacgagttgcatgataaagaagacagtcataagtgcggcgacgatagtcatgccccgcgcccaccggaaggagct
gactgggttgaaggctctcaagggcatcggtcgagatcccggtgcctaatgagtgagctaacttacattaattgcgttgcgctcactgcccgctttccagtcgggaaacctgtcgt
gccagctgcattaatgaatcggccaacgcgcggggagaggcggtttgcgtattgggcgccagggtggtttttcttttcaccagtgagacgggcaacagctgattgcccttcaccg
cctggccctgagagagttgcagcaagcggtccacgctggtttgccccagcaggcgaaaatcctgtttgatggtggttaacggcgggatataacatgagctgtcttcggtatcgtc
gtatcccactaccgagatatccgcaccaacgcgcagcccggactcggtaatggcgcgcattgcgcccagcgccatctgatcgttggcaaccagcatcgcagtgggaacgat
gccctcattcagcatttgcatggtttgttgaaaaccggacatggcactccagtcgccttcccgttccgctatcggctgaatttgattgcgagtgagatatttatgccagccagccaga
cgcagacgcgccgagacagaacttaatgggcccgctaacagcgcgatttgctggtgacccaatgcgaccagatgctccacgcccagtcgcgtaccgtcttcatgggagaaa
ataatactgttgatgggtgtctggtcagagacatcaagaaataacgccggaacattagtgcaggcagcttccacagcaatggcatcctggtcatccagcggatagttaatgatc
agcccactgacgcgttgcgcgagaagattgtgcaccgccgctttacaggcttcgacgccgcttcgttctaccatcgacaccaccacgctggcacccagttgatcggcgcgaga
tttaatcgccgcgacaatttgcgacggcgcgtgcagggccagactggaggtggcaacgccaatcagcaacgactgtttgcccgccagttgttgtgccacgcggttgggaatgt
aattcagctccgccatcgccgcttccactttttcccgcgttttcgcagaaacgtggctggcctggttcaccacgcgggaaacggtctgataagagacaccggcatactctgcgac
atcgtataacgttactggtttcacattcaccaccctgaattgactctcttccgggcgctatcatgccataccgcgaaaggttttgcgccattcgatggtgtccgggatctcgacgctctc
ccttatgcgactcctgcattaggaagcagcccagtagtaggttgaggccgttgagcaccgccgccgcaaggaatggtgcatgcaaggagatggcgcccaacagtcccccgg
ccacggggcctgccaccatacccacgccgaaacaagcgctcatgagcccgaagtggcgagcccgatcttccccatcggtgatgtcggcgatataggcgccagcaaccgca
cctgtggcgccggtgatgccggccacgatgcgtccggcgtagaggatcgagatctcgatcccgcgaaattaatacgactcactataggggaattgtgagcggataacaattcc
cctctagaaataattttgtttaactttaagaaggagatataccatgaaagtgacgaaggtaggaggcatttcgcataagaagtacacgtccgaaggccgcttagtgaagtcaga
atcggaagaaaatcgcacagacgaacgtctgtcggcgttgcttaatatgcgccttgacatgtatatcaagaatcccagcagcacggaaaccaaggaaaatcaaaaacgcatt
gggaaattaaagaaattcttctcaaacaaaatggtctatcttaaagacaataccttgagtttgaagaatgggaaaaaggagaacattgatcgtgagtattctgagactgacatcct
tgagagcgatgtccgtgacaagaaaaacttcgccgtgttgaaaaagatctatctgaatgaaaacgtgaactcggaggaattggaagtttttcgtaacgacattaagaagaaact
gaacaaaatcaacagcctgaagtactcatttgaaaagaataaggcgaattatcaaaagattaatgagaataacatcgagaaggttgaaggtaagtcaaagcgtaacattattt
acgattattatcgtgagtcagcgaaacgtgacgcttatgtaagcaatgtgaaagaagcctttgataagctttacaaggaagaggacattgcaaaacttgttcttgaaattgagaac
cttacgaagttagagaaatacaagattcgcgagttctaccacgaaattattggacgtaagaatgacaaggaaaactttgcaaaaatcatctacgaagaaatccagaatgttaat
aacatgaaagagttgatcgagaaggtaccggacatgagtgagttgaaaaagagccaagtattttacaagtattacttagacaaagaagagttgaacgacaagaacatcaaa
tacgcgttttgtcatttcgtggaaatcgaaatgagtcagttgctgaagaactacgtatataagcgcttaagtaatatctcgaatgacaaaattaagcgtatctttgaataccagaactt
gaaaaaattgatcgaaaataagctgttaaacaaacttgacacgtacgtccgtaattgtggaaagtataattattatttgcaagacggcgaaattgccacttcagatttcatcgcccg
caaccgtcagaatgaagcgtttcttcgcaacatcattggggtgtcatctgtggcctacttttctcttcgcaacattcttgaaacggagaacgagaatgatattactgggcgtatgcgc
ggcaaaacagttaagaacaataaaggtgaagagaagtacgtgtccggagaagttgataagatctataatgaaaataagaagaacgaggttaaggagaacttaaaaatgtt
ctattcgtacgatttcaatatggacaacaagaatgaaatcgaagatttcttcgccaacatcgacgaggcgatttcttccatccgtcacggtattgtccacttcaacttggaattagaa
ggtaaggatatctttgcgttcaagaacattgcgccatccgaaatctcaaagaagatgtttcagaatgagattaacgagaaaaaactgaaattgaagatctttcgtcaactgaact
ctgccaacgtgttccgctatctcgaaaagtataaaattctgaattaccttaaacgtacacgcttcgagtttgtcaataaaaatatcccattcgtcccgtctttcaccaaattatattcgcg
cattgatgacctgaagaatagtcttgggatttactggaaaactccgaaaacaaacgacgacaataagactaaggagattattgatgcccaaatctatttgcttaaaaacatctatt
acggggagttcctgaattatttcatgtcgaacaatggtaatttctttgagatttctaaagaaatcatcgaattgaacaagaacgataaacgcaacttaaagactgggttttacaagct
gcaaaagtttgaagacatccaggagaagattccaaaggaatacttggcgaatatccagtccctgtacatgattaatgccggtaatcaggacgaagaagaaaaggacacttat

attgatttcattcaaaagatcttcttaaagggatttatgacgtatcttgctaataacggtcgtttaagtctgatttacatcggctcggatgaagaaacaaatacgtcattagcagaaaa<br>gaagcaagagtttgacaagttcttgaagaagtacgagcagaacaataatatcaagatcccctatgagatcaatgaattcctgcgtgagatcaaactgggaaacatcctgaagt<br>atactgagcgtttaaacatgttctaccttatcttaaagcttttgaatcacaaggagctgacaaatctgaagggtagtcttgaaaaatatcagtctgccaataaggaagaagcgttctc<br>tgaccaattggagttaattaacctgcttaaccttgacaacaaccgcgtgacggaagacttcgaattagaggccgacgagattggaaaatttcttgatttcaatggcaacaaagtta<br>aggataacaaggaactgaaaaagttcgatacaaacaagatctactttgacggcgagaacattatcaaacaccgtgccttctacaatattaagaaatatggcatgttaaacttact<br>ggagaaaattgccgacaaggctggatacaagatctcgatcgaagagctgaagaaatactccaataaaaagaatgagatcgagaagaaccataagatgcaggaaaatctg<br>caccgcaaatacgctcgtccccgtaaagacgagaagtttacagatgaggactatgaaagttacaagcaagctattgagaatattgaggagtacacccaccttaagaacaag<br>gtagaattcaatgagctgaatttactgcagggcctgttgctgcgcattttacatcgtttagtcggatatacctcaatttgggaacgcgatctgcgcttccgccttaaaggtgagttccca<br>gaaaaccaatacatcgaagagatcttcaactttgaaaataagaagaacgtgaagtacaaagggggtcagattgtagagaaatacattaaattctacaaggaattacatcaaa<br>atgatgaagttaagatcaacaagtacagttccgcgaatatcaaggtgttgaagcaagaaaagaaggacctttatattcgaaattacatcgcccacttcaattatattcctcacgcc<br>gagatctcactgctggaagtccttgaaaatttgcgtaaattgctgtcctacgatcgcaaactgaaaaatgccgtaatgaaatcagtagttgatatccttaaggagtatggttttgtag<br>ccacattcaaaatcggggcggacaagaagatcggtattcagacactggagagcgaaaaaatcgtgcatcttaagaatcttaagaagaagaagttaatgactgaccgcaattc<br>cgaggaactttgcaaattggtgaagattatgtttgaatacaaaatggaagagaaaaagtctgaaaacggcggtggctcgcatcatcatcatcatcactaataacattggaagtg<br>gataacggatccggctgctaacaaagcccgaaaggaagctgagttggctgctgccaccgctgagcaataactagcataaccccttggggcctctaaacgggtcttgaggggt<br>ttttgctgaaaggaggaactatatccggatatcccgcaagaggcccggcagtaccggcataaccaagcctatgcctacagcatccagggtgacggtgccgaggatgacgat<br>gagcgcattgttagatttcatacacggtgcctgactgcgttagcaatttaactgtgataaactaccgcattaaagcttatcgatgataagctgtcaaacatgagaa
**Name**: pET15b_PspCas13b-6xHIS (Addgene ID = 251216)
**Map**:

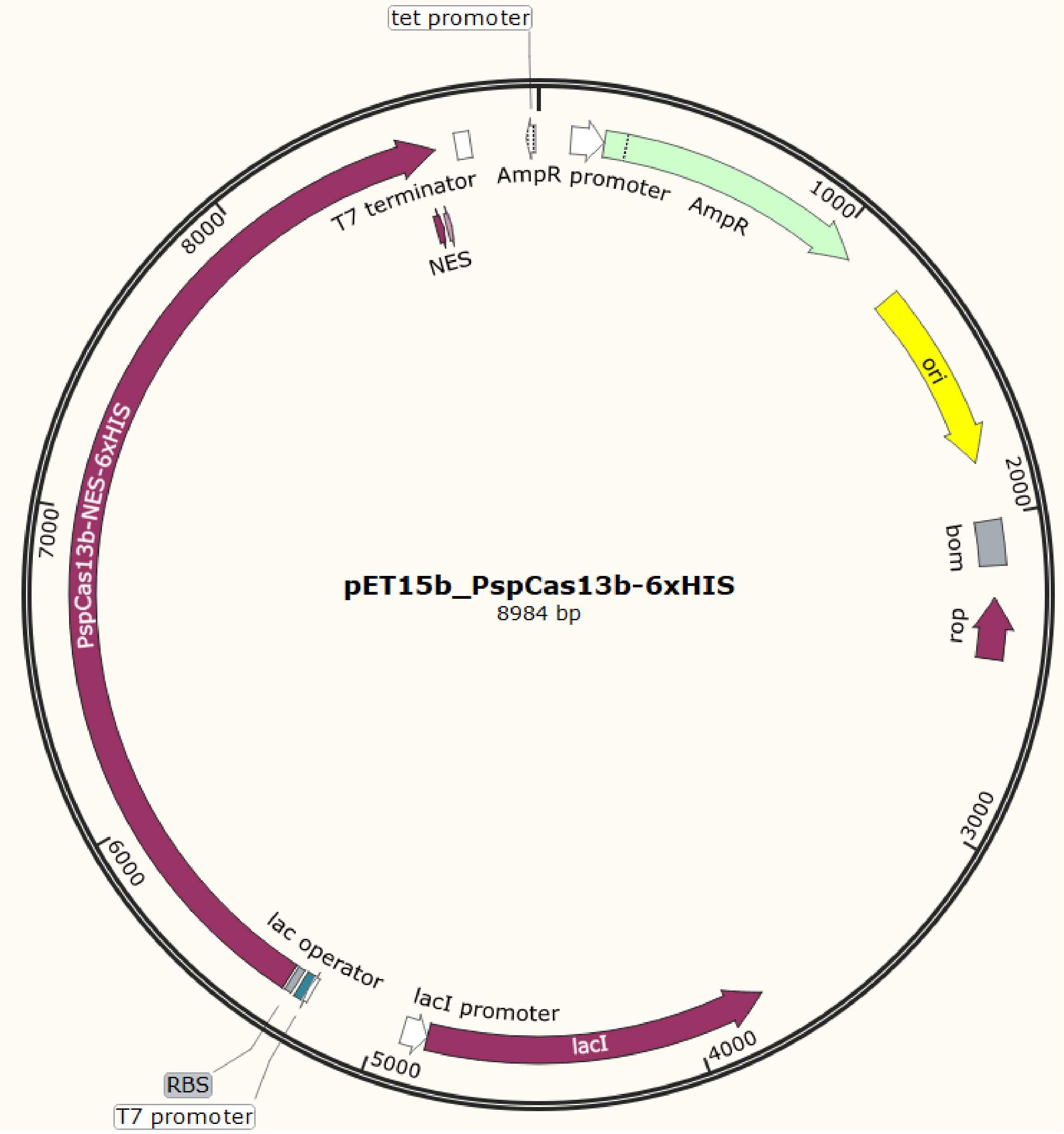

Sequence:

ttcttgaagacgaaagggcctcgtgatacgcctatttttataggttaatgtcatgataataatggtttcttagacgtcaggtggcacttttcggggaaatgtgcgcggaacccctatttgt
ttatttttctaaatacattcaaatatgtatccgctcatgagacaataaccctgataaatgcttcaataatattgaaaaaggaagagtatgagtattcaacatttccgtgtcgcccttattc
ccttttttgcggcattttgccttcctgtttttgctcacccagaaacgctggtgaaagtaaaagatgctgaagatcagttgggtgcacgagtgggttacatcgaactggatctcaacagc
ggtaagatccttgagagttttcgccccgaagaacgttttccaatgatgagcacttttaaagttctgctatgtggcgcggtattatcccgtgttgacgccgggcaagagcaactcggtc
gccgcatacactattctcagaatgacttggttgagtactcaccagtcacagaaaagcatcttacggatggcatgacagtaagagaattatgcagtgctgccataaccatgagtg
ataacactgcggccaacttacttctgacaacgatcggaggaccgaaggagctaaccgcttttttgcacaacatgggggatcatgtaactcgccttgatcgttgggaaccggagc
tgaatgaagccataccaaacgacgagcgtgacaccacgatgcctgcagcaatggcaacaacgttgcgcaaactattaactggcgaactacttactctagcttcccggcaaca
attaatagactggatggaggcggataaagttgcaggaccacttctgcgctcggcccttccggctggctggtttattgctgataaatctggagccggtgagcgtgggtctcgcggta
tcattgcagcactggggccagatggtaagccctcccgtatcgtagttatctacacgacggggagtcaggcaactatggatgaacgaaatagacagatcgctgagataggtgcc
tcactgattaagcattggtaactgtcagaccaagtttactcatatatactttagattgatttaaaacttcatttttaatttaaaaggatctaggtgaagatcctttttgataatctcatgacca
aaatcccttaacgtgagttttcgttccactgagcgtcagaccccgtagaaaagatcaaaggatcttcttgagatcctttttttctgcgcgtaatctgctgcttgcaaacaaaaaaacc
accgctaccagcggtggtttgtttgccggatcaagagctaccaactctttttccgaaggtaactggcttcagcagagcgcagataccaaatactgtccttctagtgtagccgtagtt
aggccaccacttcaagaactctgtagcaccgcctacatacctcgctctgctaatcctgttaccagtggctgctgccagtggcgataagtcgtgtcttaccgggttggactcaagac
gatagttaccggataaggcgcagcggtcgggctgaacggggggttcgtgcacacagcccagcttggagcgaacgacctacaccgaactgagatacctacagcgtgagcta

tgagaaagcgccacgcttcccgaagggagaaaggcggacaggtatccggtaagcggcagggtcggaacaggagagcgcacgagggagcttccagggggaaacgcct
ggtatctttatagtcctgtcgggtttcgccacctctgacttgagcgtcgatttttgtgatgctcgtcaggggggcggagcctatggaaaaacgccagcaacgcggcctttttacggttc
ctggccttttgctggccttttgctcacatgttctttcctgcgttatcccctgattctgtggataaccgtattaccgcctttgagtgagctgataccgctcgccgcagccgaacgaccgagc
gcagcgagtcagtgagcgaggaagcggaagagcgcctgatgcggtattttctccttacgcatctgtgcggtatttcacaccgcatatatggtgcactctcagtacaatctgctctg
atgccgcatagttaagccagtatacactccgctatcgctacgtgactgggtcatggctgcgccccgacacccgccaacacccgctgacgcgccctgacgggcttgtctgctccc
ggcatccgcttacagacaagctgtgaccgtctccgggagctgcatgtgtcagaggttttcaccgtcatcaccgaaacgcgcgaggcagctgcggtaaagctcatcagcgtggt
cgtgaagcgattcacagatgtctgcctgttcatccgcgtccagctcgttgagtttctccagaagcgttaatgtctggcttctgataaagcgggccatgttaagggcggtttttcctgttt
ggtcactgatgcctccgtgtaagggggatttctgttcatgggggtaatgataccgatgaaacgagagaggatgctcacgatacgggttactgatgatgaacatgcccggttactg
gaacgttgtgagggtaaacaactggcggtatggatgcggcgggaccagagaaaaatcactcagggtcaatgccagcgcttcgttaatacagatgtaggtgttccacagggta
gccagcagcatcctgcgatgcagatccggaacataatggtgcagggcgctgacttccgcgtttccagactttacgaaacacggaaaccgaagaccattcatgttgttgctcagg
tcgcagacgttttgcagcagcagtcgcttcacgttcgctcgcgtatcggtgattcattctgctaaccagtaaggcaaccccgccagcctagccgggtcctcaacgacaggagca
cgatcatgcgcacccgtggccaggacccaacgctgcccgagatgcgccgcgtgcggctgctggagatggcggacgcgatggatatgttctgccaagggttggtttgcgcattc
acagttctccgcaagaattgattggctccaattcttggagtggtgaatccgttagcgaggtgccgccggcttccattcaggtcgaggtggcccggctccatgcaccgcgacgcaa
cgcggggaggcagacaaggtatagggcggcgcctacaatccatgccaacccgttccatgtgctcgccgaggcggcataaatcgccgtgacgatcagcggtccagtgatcg
aagttaggctggtaagagccgcgagcgatccttgaagctgtccctgatggtcgtcatctacctgcctggacagcatggcctgcaacgcgggcatcccgatgccgccggaagc
gagaagaatcataatggggaaggccatccagcctcgcgtcgcgaacgccagcaagacgtagcccagcgcgtcggccgccatgccggcgataatggcctgcttctcgccga
aacgtttggtggcgggaccagtgacgaaggcttgagcgagggcgtgcaagattccgaataccgcaagcgacaggccgatcatcgtcgcgctccagcgaaagcggtcctcg
ccgaaaatgacccagagcgctgccggcacctgtcctacgagttgcatgataaagaagacagtcataagtgcggcgacgatagtcatgccccgcgcccaccggaaggagct
gactgggttgaaggctctcaagggcatcggtcgagatcccggtgcctaatgagtgagctaacttacattaattgcgttgcgctcactgcccgctttccagtcgggaaacctgtcgt
gccagctgcattaatgaatcggccaacgcgcggggagaggcggtttgcgtattgggcgccagggtggtttttcttttcaccagtgagacgggcaacagctgattgcccttcaccg
cctggccctgagagagttgcagcaagcggtccacgctggtttgccccagcaggcgaaaatcctgtttgatggtggttaacggcgggatataacatgagctgtcttcggtatcgtc
gtatcccactaccgagatatccgcaccaacgcgcagcccggactcggtaatggcgcgcattgcgcccagcgccatctgatcgttggcaaccagcatcgcagtgggaacgat
gccctcattcagcatttgcatggtttgttgaaaaccggacatggcactccagtcgccttcccgttccgctatcggctgaatttgattgcgagtgagatatttatgccagccagccaga
cgcagacgcgccgagacagaacttaatgggcccgctaacagcgcgatttgctggtgacccaatgcgaccagatgctccacgcccagtcgcgtaccgtcttcatgggagaaa
ataatactgttgatgggtgtctggtcagagacatcaagaaataacgccggaacattagtgcaggcagcttccacagcaatggcatcctggtcatccagcggatagttaatgatc
agcccactgacgcgttgcgcgagaagattgtgcaccgccgctttacaggcttcgacgccgcttcgttctaccatcgacaccaccacgctggcacccagttgatcggcgcgaga
tttaatcgccgcgacaatttgcgacggcgcgtgcagggccagactggaggtggcaacgccaatcagcaacgactgtttgcccgccagttgttgtgccacgcggttgggaatgt
aattcagctccgccatcgccgcttccactttttcccgcgttttcgcagaaacgtggctggcctggttcaccacgcgggaaacggtctgataagagacaccggcatactctgcgac
atcgtataacgttactggtttcacattcaccaccctgaattgactctcttccgggcgctatcatgccataccgcgaaaggttttgcgccattcgatggtgtccgggatctcgacgctctc
ccttatgcgactcctgcattaggaagcagcccagtagtaggttgaggccgttgagcaccgccgccgcaaggaatggtgcatgcaaggagatggcgcccaacagtcccccgg
ccacggggcctgccaccatacccacgccgaaacaagcgctcatgagcccgaagtggcgagcccgatcttccccatcggtgatgtcggcgatataggcgccagcaaccgca
cctgtggcgccggtgatgccggccacgatgcgtccggcgtagaggatcgagatctcgatcccgcgaaattaatacgactcactataggggaattgtgagcggataacaattcc
cctctagaaataattttgtttaactttaagaaggagatataccatgaacatccccgctctggtggaaaaccagaagaagtactttggcacctacagcgtgatggccatgctgaac
gctcagaccgtgctggaccacatccagaaggtggccgatattgagggcgagcagaacgagaacaacgagaatctgtggtttcaccccgtgatgagccacctgtacaacgcc
aagaacggctacgacaagcagcccgagaaaaccatgttcatcatcgagcggctgcagagctacttcccattcctgaagatcatggccgagaaccagagagagtacagca
acggcaagtacaagcagaaccgcgtggaagtgaacagcaacgacatcttcgaggtgctgaagcgcgccttcggcgtgctgaagatgtacagggacctgaccaaccacta
caagacctacgaggaaaagctgaacgacggctgcgagttcctgaccagcacagagcaacctctgagcggcatgatcaacaactactacacagtggccctgcggaacatg
aacgagagatacggctacaagacagaggacctggccttcatccaggacaagcggttcaagttcgtgaaggacgcctacggcaagaaaaaagtcccaagtgaataccggatt
cttcctgagcctgcaggactacaacggcgacacacagaagaagctgcacctgagcggagtgggaatcgccctgctgatctgcctgttcctggacaagcagtacatcaacatc
tttctgagcaggctgcccatcttctccagctacaatgcccagagcgaggaacggcggatcatcatcagatccttcggcatcaacagcatcaagctgcccaaggaccggatcca
cagcgagaagtccaacaagagcgtggccatggatatgctcaacgaagtgaagcggtgccccgacgagctgttcacaacactgtctgccgagaagcagtcccggttcagaa
tcatcagcgacgaccacaatgaagtgctgatgaagcggagcagcgacagattcgtgcctctgctgctgcagtatatcgattacggcaagctgttcgaccacatcaggttccacg
tgaacatgggcaagctgagatacctgctgaaggccgacaagacctgcatcgacggccagaccagagtcagagtgatcgagcagcccctgaacggcttcggcagactgga
agaggccgagacaatgcggaagcaagagaacggcaccttcggcaacagcggcatccggatcagagacttcgagaacatgaagcgggacgacgccaatcctgccaact
atccctacatcgtggacacctacacacactacatcctggaaaacaacaaggtcgagatgtttatcaacgacaaagaggacagcgccccactgctgcccgtgatcgaggatg
atagatacgtggtcaagacaatccccagctgccggatgagcaccctggaaattccagccatggccttccacatgtttctgttcggcagcaagaaaaccgagaagctgatcgtg
gacgtgcacaaccggtacaagagactgttccaggccatgcagaaagaagaagtgaccgccgagaatatcgccagcttcggaatcgccgagagcgacctgcctcagaag
atcctggatctgatcagcggcaatgcccacggcaaggatgtggacgccttcatcagactgaccgtggacgacatgctgaccgacaccgagcggagaatcaagagattcaa
ggacgaccggaagtccattcggagcgccgacaacaagatgggaaagagaggcttcaagcagatctccacaggcaagctggccgacttcctggccaaggacatcgtgctg
tttcagcccagcgtgaacgatggcgagaacaagatcaccggcctgaactaccggatcatgcagagcgccattgccgtgtacgatagcggcgacgattacgaggccaagca

gcagttcaagctgatgttcgagaaggcccggctgatcggcaagggcacaacagagcctcatccatttctgtacaaggtgttcgcccgcagcatccccgccaatgccgtcgagt<br>tctacgagcgctacctgatcgagcggaagttctacctgaccggcctgtccaacgagatcaagaaaggcaacagagtggatgtgcccttcatccggcgggaccagaacaagt<br>ggaaaacacccgccatgaagaccctgggcagaatctacagcgaggatctgcccgtggaactgcccagacagatgttcgacaatgagatcaagtcccacctgaagtccctg<br>ccacagatggaaggcatcgacttcaacaatgccaacgtgacctatctgatcgccgagtacatgaagagagtgctggacgacgacttccagaccttctaccagtggaaccgca<br>actaccggtacatggacatgcttaagggcgagtacgacagaaagggctccctgcagcactgcttcaccagcgtggaagagagagaaggcctctggaaagagcgggcctc<br>cagaacagagcggtacagaaagcaggccagcaacaagatccgcagcaaccggcagatgagaaacgccagcagcgaagagatcgagacaatcctggataagcggct<br>gagcaacagccggaacgagtaccagaaaagcgagaaagtgatccggcgctacagagtgcaggatgccctgctgtttctgctggccaaaaagaccctgaccgaactggcc<br>gatttcgacggcgagaggttcaaactgaaagaaatcatgcccgacgccgagaagggaatcctgagcgagatcatgcccatgagcttcaccttcgagaaaggcggcaagaa<br>gtacaccatcaccagcgagggcatgaagctgaagaactacggcgacttctttgtgctggctagcgacaagaggatcggcaacctgctggaactcgtgggcagcgacatcgt<br>gtccaaagaggatatcatggaagagttcaacaaatacgaccagtgcaggcccgagatcagctccatcgtgttcaacctggaaaagtgggccttcgacacataccccgagct<br>gtctgccagagtggaccgggaagagaaggtggacttcaagagcatcctgaaaatcctgctgaacaacaagaacatcaacaaagagcagagcgacatcctgcggaagat<br>ccggaacgccttcgatcacaacaattaccccgacaaaggcgtggtggaaatcaaggccctgcctgagatcgccatgagcatcaagaaggcctttggggagtacgccatcat<br>gaagggatcccttcaactgcctccacttgaaagactgacactgggcggtggctcgcatcatcatcatcatcactaataagatccggctgctaacaaagcccgaaaggaagctg<br>agttggctgctgccaccgctgagcaataactagcataaccccttggggcctctaaacgggtcttgaggggttttttgctgaaaggaggaactatatccggatatcccgcaagagg<br>cccggcagtaccggcataaccaagcctatgcctacagcatccagggtgacggtgccgaggatgacgatgagcgcattgttagatttcatacacggtgcctgactgcgttagca<br>atttaactgtgataaactaccgcattaaagcttatcgatgataagctgtcaaacatgagaa
**Name**: pET15b_BzCas13b-6xHIS (Addgene ID = 251217)
**Map**:

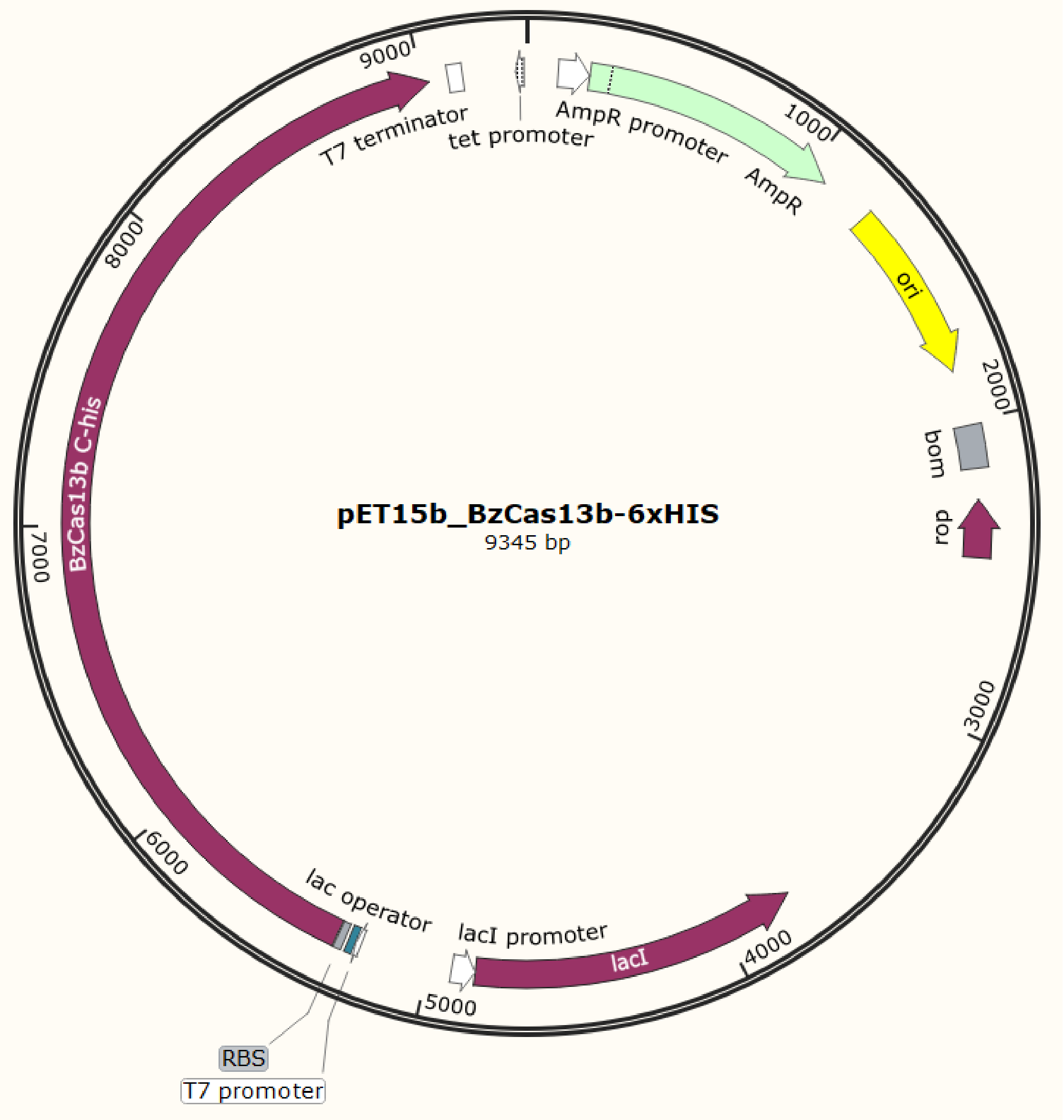

Sequence:

ttcttgaagacgaaagggcctcgtgatacgcctatttttataggttaatgtcatgataataatggtttcttagacgtcaggtggcacttttcggggaaatgtgcgcggaacccctatttgt
ttatttttctaaatacattcaaatatgtatccgctcatgagacaataaccctgataaatgcttcaataatattgaaaaaggaagagtatgagtattcaacatttccgtgtcgcccttattc
ccttttttgcggcattttgccttcctgtttttgctcacccagaaacgctggtgaaagtaaaagatgctgaagatcagttgggtgcacgagtgggttacatcgaactggatctcaacagc
ggtaagatccttgagagttttcgccccgaagaacgttttccaatgatgagcacttttaaagttctgctatgtggcgcggtattatcccgtgttgacgccgggcaagagcaactcggtc
gccgcatacactattctcagaatgacttggttgagtactcaccagtcacagaaaagcatcttacggatggcatgacagtaagagaattatgcagtgctgccataaccatgagtg
ataacactgcggccaacttacttctgacaacgatcggaggaccgaaggagctaaccgcttttttgcacaacatgggggatcatgtaactcgccttgatcgttgggaaccggagc
tgaatgaagccataccaaacgacgagcgtgacaccacgatgcctgcagcaatggcaacaacgttgcgcaaactattaactggcgaactacttactctagcttcccggcaaca
attaatagactggatggaggcggataaagttgcaggaccacttctgcgctcggcccttccggctggctggtttattgctgataaatctggagccggtgagcgtgggtctcgcggta
tcattgcagcactggggccagatggtaagccctcccgtatcgtagttatctacacgacggggagtcaggcaactatggatgaacgaaatagacagatcgctgagataggtgcc
tcactgattaagcattggtaactgtcagaccaagtttactcatatatactttagattgatttaaaacttcatttttaatttaaaaggatctaggtgaagatcctttttgataatctcatgacca
aaatcccttaacgtgagttttcgttccactgagcgtcagaccccgtagaaaagatcaaaggatcttcttgagatcctttttttctgcgcgtaatctgctgcttgcaaacaaaaaaacc
accgctaccagcggtggtttgtttgccggatcaagagctaccaactctttttccgaaggtaactggcttcagcagagcgcagataccaaatactgtccttctagtgtagccgtagtt
aggccaccacttcaagaactctgtagcaccgcctacatacctcgctctgctaatcctgttaccagtggctgctgccagtggcgataagtcgtgtcttaccgggttggactcaagac
gatagttaccggataaggcgcagcggtcgggctgaacggggggttcgtgcacacagcccagcttggagcgaacgacctacaccgaactgagatacctacagcgtgagcta
tgagaaagcgccacgcttcccgaagggagaaaggcggacaggtatccggtaagcggcagggtcggaacaggagagcgcacgagggagcttccagggggaaacgcct

ggtatctttatagtcctgtcgggtttcgccacctctgacttgagcgtcgatttttgtgatgctcgtcaggggggcggagcctatggaaaaacgccagcaacgcggcctttttacggttc
ctggccttttgctggccttttgctcacatgttctttcctgcgttatcccctgattctgtggataaccgtattaccgcctttgagtgagctgataccgctcgccgcagccgaacgaccgagc
gcagcgagtcagtgagcgaggaagcggaagagcgcctgatgcggtattttctccttacgcatctgtgcggtatttcacaccgcatatatggtgcactctcagtacaatctgctctg
atgccgcatagttaagccagtatacactccgctatcgctacgtgactgggtcatggctgcgccccgacacccgccaacacccgctgacgcgccctgacgggcttgtctgctccc
ggcatccgcttacagacaagctgtgaccgtctccgggagctgcatgtgtcagaggttttcaccgtcatcaccgaaacgcgcgaggcagctgcggtaaagctcatcagcgtggt
cgtgaagcgattcacagatgtctgcctgttcatccgcgtccagctcgttgagtttctccagaagcgttaatgtctggcttctgataaagcgggccatgttaagggcggtttttcctgttt
ggtcactgatgcctccgtgtaagggggatttctgttcatgggggtaatgataccgatgaaacgagagaggatgctcacgatacgggttactgatgatgaacatgcccggttactg
gaacgttgtgagggtaaacaactggcggtatggatgcggcgggaccagagaaaaatcactcagggtcaatgccagcgcttcgttaatacagatgtaggtgttccacagggta
gccagcagcatcctgcgatgcagatccggaacataatggtgcagggcgctgacttccgcgtttccagactttacgaaacacggaaaccgaagaccattcatgttgttgctcagg
tcgcagacgttttgcagcagcagtcgcttcacgttcgctcgcgtatcggtgattcattctgctaaccagtaaggcaaccccgccagcctagccgggtcctcaacgacaggagca
cgatcatgcgcacccgtggccaggacccaacgctgcccgagatgcgccgcgtgcggctgctggagatggcggacgcgatggatatgttctgccaagggttggtttgcgcattc
acagttctccgcaagaattgattggctccaattcttggagtggtgaatccgttagcgaggtgccgccggcttccattcaggtcgaggtggcccggctccatgcaccgcgacgcaa
cgcggggaggcagacaaggtatagggcggcgcctacaatccatgccaacccgttccatgtgctcgccgaggcggcataaatcgccgtgacgatcagcggtccagtgatcg
aagttaggctggtaagagccgcgagcgatccttgaagctgtccctgatggtcgtcatctacctgcctggacagcatggcctgcaacgcgggcatcccgatgccgccggaagc
gagaagaatcataatggggaaggccatccagcctcgcgtcgcgaacgccagcaagacgtagcccagcgcgtcggccgccatgccggcgataatggcctgcttctcgccga
aacgtttggtggcgggaccagtgacgaaggcttgagcgagggcgtgcaagattccgaataccgcaagcgacaggccgatcatcgtcgcgctccagcgaaagcggtcctcg
ccgaaaatgacccagagcgctgccggcacctgtcctacgagttgcatgataaagaagacagtcataagtgcggcgacgatagtcatgccccgcgcccaccggaaggagct
gactgggttgaaggctctcaagggcatcggtcgagatcccggtgcctaatgagtgagctaacttacattaattgcgttgcgctcactgcccgctttccagtcgggaaacctgtcgt
gccagctgcattaatgaatcggccaacgcgcggggagaggcggtttgcgtattgggcgccagggtggtttttcttttcaccagtgagacgggcaacagctgattgcccttcaccg
cctggccctgagagagttgcagcaagcggtccacgctggtttgccccagcaggcgaaaatcctgtttgatggtggttaacggcgggatataacatgagctgtcttcggtatcgtc
gtatcccactaccgagatatccgcaccaacgcgcagcccggactcggtaatggcgcgcattgcgcccagcgccatctgatcgttggcaaccagcatcgcagtgggaacgat
gccctcattcagcatttgcatggtttgttgaaaaccggacatggcactccagtcgccttcccgttccgctatcggctgaatttgattgcgagtgagatatttatgccagccagcc aga
cgcagacgcgccgagacagaacttaatgggcccgctaacagcgcgatttgctggtgacccaatgcgaccagatgctccacgcccagtcgcgtaccgtcttcatgggagaaa
ataatactgttgatgggtgtctggtcagagacatcaagaaataacgccggaacattagtgcaggcagcttccacagcaatggcatcctggtcatccagcggatagttaatgatc
agcccactgacgcgttgcgcgagaagattgtgcaccgccgctttacaggcttcgacgccgcttcgttctaccatcgacaccaccacgctggcacccagttgatcggcgcgaga
tttaatcgccgcgacaatttgcgacggcgcgtgcagggccagactggaggtggcaacgccaatcagcaacgactgtttgcccgccagttgttgtgccacgcggttgggaatgt
aattcagctccgccatcgccgcttccactttttcccgcgttttcgcagaaacgtggctggcctggttcaccacgcgggaaacggtctgataagagacaccggcatactctgcgac
atcgtataacgttactggtttcacattcaccaccctgaattgactctcttccgggcgctatcatgccataccgcgaaaggttttgcgccattcgatggtgtccgggatctcgacgctctc
ccttatgcgactcctgcattaggaagcagcccagtagtaggttgaggccgttgagcaccgccgccgcaaggaatggtgcatgcaaggagatggcgcccaacagtcccccgg
ccacggggcctgccaccatacccacgccgaaacaagcgctcatgagcccgaagtggcgagcccgatcttccccatcggtgatgtcggcgatataggcgccagcaaccgca
cctgtggcgccggtgatgccggccacgatgcgtccggcgtagaggatcgagatctcgatcccgcgaaattaatacgactcactataggggaattgtgagcggataacaattcc
cctctagaaataattttgtttaactttaagaaggagagttaggaggtctttatcatggaaaacaaaacatctttggggaataatatttattacaacccttttaaaccacaagataaatc
gtattttgcgggttatttcaatgcagcaatggaaaatacagatagtgtatttagagaattgggaaaaagattaaaaggtaaagagtacacttcggagaatttttttgatgctatcttta
aggaaaatatttcattagtggaatacgaaaggtatgtgaaattgctttctgattatttccctatggctcgtcttttggataaaaaagaagttcctataaaagaacgaaaagaaaatttt
aaaaagaattttaaaggcattataaaagccgtaagagatttaagaaacttttatacgcataaggaacacggagaggtagaaattacagatgaaatatttggcgtattggatgag
atgctgaaaagcacggttttaacggtaaaaaagaaaaaagtaaaaaccgataaaacaaaagaaatcctgaaaaaaagcatagaaaaacaattggatattttatgccaaaa
gaaattagaatatctaagagacacagcaagaaaaatagaggagaagcgtagaaatcaaagagaaagaggagagaaagaattggtagctccattcaaatacagtgataa
aagggatgatttaatagcagcgatttataatgatgcttttgatgtttatatcgataagaaaaaagatagcctaaaagaatccagcaaagcaaagtacaataccaaaagtgatcct
cagcaagaagaaggcgatttgaaaattccaatttcgaaaaacggagtagtcttcctgctttctttgtttctcaccaaacaagaaatacacgcattcaaatctaaaatagcagggttt
aaggctacggttatcgatgaagcaacggtatctgaagcaacggtgagccatggaaaaaacagcatttgttttatggctacgcacgaaattttctcgcatttggcttacaaaaaatt
gaaacgaaaagtgagaacggctgaaatcaactatggtgaggcagaaaatgctgaacagctttccgtttatgccaaagaaactctaatgatgcagatgcttgatgaattgagca
aagtacccgatgtggtctatcagaatttgagcgaagatgtacaaaaaactttcatcgaagattggaatgaatacctaaaagagaacaatggcgatgtagggacaatggaaga
agagcaggtaattcatccagtgattcgcaagcgttacgaagataaattcaattattttgccattcgatttttagatgagtttgctcagttccctacgcttcgttttcaagtgcatttaggga
attatcttcacgattctcgccctaaagaaaatctgatttctgaccgaagaataaaagaaaaaatcaccgttttcggaaggctttcggaattggaacataaaaaagcattgtttataa
aaaataccgaaaccaatgaagatagagaacactattgggaaatttttcctaaccccaattatgattttcctaaggaaaatatttccgttaatgataaagattttcctatcgcgggtag
tattttggacagagagaaacagcctgtcgcagggaaaattggtataaaagtgaaattgcttaaccaacaatatgtttcagaagtagataaagccgtaaaagcccatcagctca
agcaaagaaaagccagcaagccgtctatccagaacattatagaagaaatagtgccaatcaatgaaagcaatccaaaagaagctattgttttcggtgggcaacccacggctt
atctgagtatgaatgacattcattccattttgtatgagtttttcgataaatgggagaaaaagaaagaaaagttagaaaagaaaggtgaaaaagaattaagaaaggaaattggaa
aagagttagaaaagaaaatcgtaggaaaaattcaagctcaaatccagcaaatcatagataaagataccaatgccaagatattaaaccttatcaagacggaaattctactgc
tattgataaagagaaattgataaaagatttgaagcaagagcaaaacattctacaaaaattaaaagatgaacaaacggttcgtgaaaaagaatataatgattttattgcttatcaa

gataaaaatagggagataaataaagtcagagatagaaatcataaacaatatttaaaagataaccttaaacgaaagtaccctgaagctcctgcacgaaaagaagttctttatta
tcgagaaaaaggaaaagtagcggtttggttggcgaatgacatcaaacgctttatgcctaccgatttcaaaaacgaatggaaaggggagcaacacagcttgttacaaaaatcg
ttggcgtattatgagcaatgtaaagaggagttgaaaaatcttttgcctgaaaaagtatttcagcatttaccttttaagttgggaggatattttcaacaaaaatatttgtaccaattttaca
cttgttatttagataaacggttggaatacatcagtgggttggtgcaacaagccgaaaactttaaatccgaaaataaagtcttcaaaaaggtagaaaatgagtgttttaaatttttgaa
aaaacaaaattatactcacaaagaattagatgctcgggttcagtctatattggggtatcctatctttttagagcggggctttatggatgaaaaacctacgataatcaaaggaaaaa
cttttaaaggaaatgaagctctttttgccgattggtttaggtattacaaggaatatcagaattttcagacattttatgatactgaaaattatccgcttgtagaattggaaaaaaaacaag
cagaccgaaaacgaaaaactaaaatctaccaacaaaagaaaaacgatgtgtttacacttttgatggcaaaacatattttcaaatcggtattcaagcaggatagcatcgatcagt
tttcgttagaagatttgtatcaatctcgggaggaacgcttaggaaatcaggagagagccagacaaacgggcgagcgaaatactaactatatttggaacaaaaccgttgatttaa
agttgtgtgacggaaaaattacggtagaaaatgtaaagctaaaaaatgtaggagatttcataaaatatgaatacgaccaaagggttcaagctttcttaaaatatgaggaaaata
tagaatggcaggcgtttctaatcaaagagagtaaggaagaggaaaattatccgtatgtggtggaaagagaaatagagcaatatgaaaaagtgagaagggaagaactgttg
aaggaagtccaccttatagaggaatatattttggaaaaggtaaaagataaggaaatattaaaaaaaggggataatcaaaatttcaaatactacattctcaacggacttttaaag
caacttaaaaatgaagatgtggagagctataaggtattcaacttaaatactgaacctgaagatgtgaacattaaccaactaaaacaagaagcaaccgatttagaacaaaaag
cttttgttttaacctatattcgtaataaatttgcacacaaccaattacctaaaaaagaattttgggactattgtcaagaaaaatacggcaaaatagaaaaagaaaaaacctatgcc
gagtattttgctgaggtatttaaaaaggaaaaagaagcactgataaagggcggtggctcgcatcatcatcatcatcactaaggatccggctgctaacaaagcccgaaaggaa
gctgagttggctgctgccaccgctgagcaataactagcataaccccttggggcctctaaacgggtcttgaggggttttttgctgaaaggaggaactatatccggatatcccgcaa
gaggcccggcagtaccggcataaccaagcctatgcctacagcatccagggtgacggtgccgaggatgacgatgagcgcattgttagatttcatacacggtgcctgactgcgtt
agcaatttaactgtgataaactaccgcattaaagcttatcgatgataagctgtcaaacatgagaa

Name: pET15b_RspCas13d-6xHIS (Addgene ID = 251218)

Map:

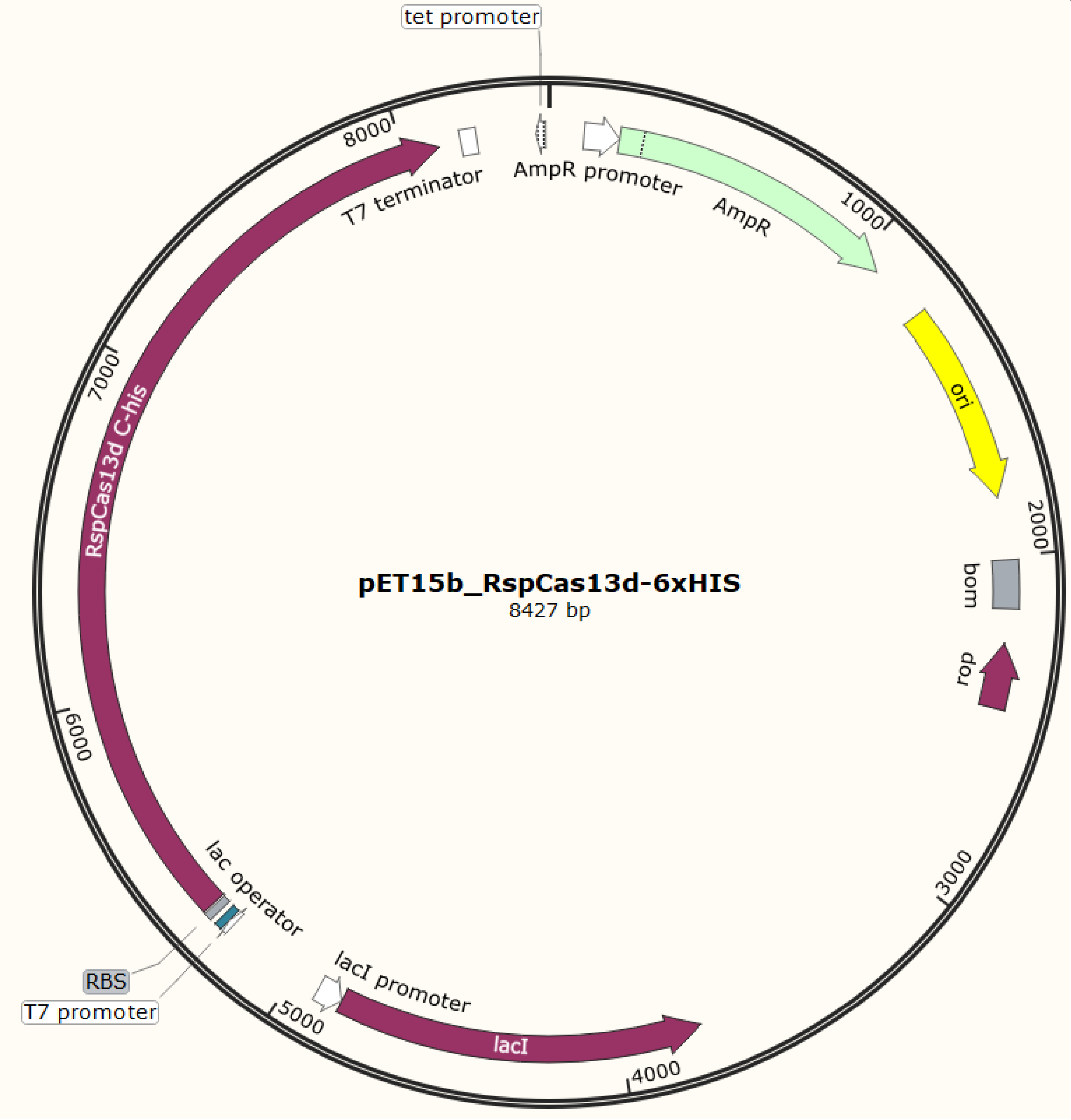

Sequence:

ttcttgaagacgaaagggcctcgtgatacgcctatttttataggttaatgtcatgataataatggtttcttagacgtcaggtggcacttttcggggaaatgtgcgcggaacccctatttgt
ttatttttctaaatacattcaaatatgtatccgctcatgagacaataaccctgataaatgcttcaataatattgaaaaaggaagagtatgagtattcaacatttccgtgtcgcccttattc
ccttttttgcggcattttgccttcctgtttttgctcacccagaaacgctggtgaaagtaaaagatgctgaagatcagttgggtgcacgagtgggttacatcgaactggatctcaacagc
ggtaagatccttgagagttttcgccccgaagaacgttttccaatgatgagcacttttaaagttctgctatgtggcgcggtattatcccgtgttgacgccgggcaagagcaactcggtc
gccgcatacactattctcagaatgacttggttgagtactcaccagtcacagaaaagcatcttacggatggcatgacagtaagagaattatgcagtgctgccataaccatgagtg
ataacactgcggccaacttacttctgacaacgatcggaggaccgaaggagctaaccgcttttttgcacaacatgggggatcatgtaactcgccttgatcgttgggaaccggagc
tgaatgaagccataccaaacgacgagcgtgacaccacgatgcctgcagcaatggcaacaacgttgcgcaaactattaactggcgaactacttactctagcttcccggcaaca
attaatagactggatggaggcggataaagttgcaggaccacttctgcgctcggcccttccggctggctggtttattgctgataaatctggagccggtgagcgtgggtctcgcggta
tcattgcagcactggggccagatggtaagccctcccgtatcgtagttatctacacgacggggagtcaggcaactatggatgaacgaaatagacagatcgctgagataggtgcc
tcactgattaagcattggtaactgtcagaccaagtttactcatatatactttagattgatttaaaacttcatttttaatttaaaaggatctaggtgaagatcctttttgataatctcatgacca
aaatcccttaacgtgagttttcgttccactgagcgtcagaccccgtagaaaagatcaaaggatcttcttgagatcctttttttctgcgcgtaatctgctgcttgcaaacaaaaaaacc
accgctaccagcggtggtttgtttgccggatcaagagctaccaactctttttccgaaggtaactggcttcagcagagcgcagataccaaatactgtccttctagtgtagccgtagtt
aggccaccacttcaagaactctgtagcaccgcctacatacctcgctctgctaatcctgttaccagtggctgctgccagtggcgataagtcgtgtcttaccgggttggactcaagac
gatagttaccggataaggcgcagcggtcgggctgaacggggggttcgtgcacacagcccagcttggagcgaacgacctacaccgaactgagatacctacagcgtgagcta
tgagaaagcgccacgcttcccgaagggagaaaggcggacaggtatccggtaagcggcagggtcggaacaggagagcgcacgagggagcttccagggggaaacgcct

ggtatctttatagtcctgtcgggtttcgccacctctgacttgagcgtcgatttttgtgatgctcgtcaggggggcggagcctatggaaaaacgccagcaacgcggcctttttacggttc
ctggccttttgctggccttttgctcacatgttctttcctgcgttatcccctgattctgtggataaccgtattaccgcctttgagtgagctgataccgctcgccgcagccgaacgaccgagc
gcagcgagtcagtgagcgaggaagcggaagagcgcctgatgcggtattttctccttacgcatctgtgcggtatttcacaccgcatatatggtgcactctcagtacaatctgctctg
atgccgcatagttaagccagtatacactccgctatcgctacgtgactgggtcatggctgcgccccgacacccgccaacacccgctgacgcgccctgacgggcttgtctgctccc
ggcatccgcttacagacaagctgtgaccgtctccgggagctgcatgtgtcagaggttttcaccgtcatcaccgaaacgcgcgaggcagctgcggtaaagctcatcagcgtggt
cgtgaagcgattcacagatgtctgcctgttcatccgcgtccagctcgttgagtttctccagaagcgttaatgtctggcttctgataaagcgggccatgttaagggcggtttttttcctgttt
ggtcactgatgcctccgtgtaagggggatttctgttcatgggggtaatgataccgatgaaacgagagaggatgctcacgatacgggttactgatgatgaacatgcccggttactg
gaacgttgtgagggtaaacaactggcggtatggatgcggcgggaccagagaaaaatcactcagggtcaatgccagcgcttcgttaatacagatgtaggtgttccacagggta
gccagcagcatcctgcgatgcagatccggaacataatggtgcagggcgctgacttccgcgtttccagactttacgaaacacggaaaccgaagaccattcatgttgttgctcagg
tcgcagacgttttgcagcagcagtcgcttcacgttcgctcgcgtatcggtgattcattctgctaaccagtaaggcaaccccgccagcctagccgggtcctcaacgacaggagca
cgatcatgcgcacccgtggccaggacccaacgctgcccgagatgcgccgcgtgcggctgctggagatggcggacgcgatggatatgttctgccaagggttggtttgcgcattc
acagttctccgcaagaattgattggctccaattcttggagtggtgaatccgttagcgaggtgccgccggcttccattcaggtcgaggtggcccggctccatgcaccgcgacgcaa
cgcggggaggcagacaaggtatagggcggcgcctacaatccatgccaacccgttccatgtgctcgccgaggcggcataaatcgccgtgacgatcagcggtccagtgatcg
aagttaggctggtaagagccgcgagcgatccttgaagctgtccctgatggtcgtcatctacctgcctggacagcatggcctgcaacgcgggcatcccgatgccgccggaagc
gagaagaatcataatggggaaggccatccagcctcgcgtcgcgaacgccagcaagacgtagcccagcgcgtcggccgccatgccggcgataatggcctgcttctcgccga
aacgtttggtggcgggaccagtgacgaaggcttgagcgagggcgtgcaagattccgaataccgcaagcgacaggccgatcatcgtcgcgctccagcgaaagcggtcctcg
ccgaaaatgacccagagcgctgccggcacctgtcctacgagttgcatgataaagaagacagtcataagtgcggcgacgatagtcatgccccgcgcccaccggaaggagct
gactgggttgaaggctctcaagggcatcggtcgagatcccggtgcctaatgagtgagctaacttacattaattgcgttgcgctcactgcccgctttccagtcgggaaacctgtcgt
gccagctgcattaatgaatcggccaacgcgcggggagaggcggtttgcgtattgggcgccagggtggtttttcttttcaccagtgagacgggcaacagctgattgcccttcaccg
cctggccctgagagagttgcagcaagcggtccacgctggtttgccccagcaggcgaaaatcctgtttgatggtggttaacggcgggatataacatgagctgtcttcggtatcgtc
gtatcccactaccgagatatccgcaccaacgcgcagcccggactcggtaatggcgcgcattgcgcccagcgccatctgatcgttggcaaccagcatcgcagtgggaacgat
gccctcattcagcatttgcatggtttgttgaaaaccggacatggcactccagtcgccttcccgttccgctatcggctgaatttgattgcgagtgagatatttatgccagccagccaga
cgcagacgcgccgagacagaacttaatgggcccgctaacagcgcgatttgctggtgacccaatgcgaccagatgctccacgcccagtcgcgtaccgtcttcatgggagaaa
ataatactgttgatgggtgtctggtcagagacatcaagaaataacgccggaacattagtgcaggcagcttccacagcaatggcatcctggtcatccagcggatagttaatgatc
agcccactgacgcgttgcgcgagaagattgtgcaccgccgctttacaggcttcgacgccgcttcgttctaccatcgacaccaccacgctggcacccagttgatcggcgcgaga
tttaatcgccgcgacaatttgcgacggcgcgtgcagggccagactggaggtggcaacgccaatcagcaacgactgtttgcccgccagttgttgtgccacgcggttgggaatgt
aattcagctccgccatcgccgcttccactttttcccgcgttttcgcagaaacgtggctggcctggttcaccacgcgggaaacggtctgataagagacaccggcatactctgcgac
atcgtataacgttactggtttcacattcaccaccctgaattgactctcttccgggcgctatcatgccataccgcgaaaggttttgcgccattcgatggtgtccgggatctcgacgctctc
ccttatgcgactcctgcattaggaagcagcccagtagtaggttgaggccgttgagcaccgccgccgcaaggaatggtgcatgcaaggagatggcgcccaacagtcccccgg
ccacggggcctgccaccatacccacgccgaaacaagcgctcatgagcccgaagtggcgagcccgatcttccccatcggtgatgtcggcgatataggcgccagcaaccgca
cctgtggcgccggtgatgccggccacgatgcgtccggcgtagaggatcgagatctcgatcccgcgaaattaatacgactcactataggggaattgtgagcggataacaattcc
cctctagaaataattttgtttaactttaagaaggagatataccatggccaaaaagaacaaaatgaaaccgcgtgaactgcgtgaagcacagaaaaaagcacgtcagctgaa
agcagcagaaattaacaataatgcagcaccggcaattgcagcaatgcctgcagcagaagttattgcaccggttgcagaaaaaaagaaaagcagcgttaaagcagccggt
atgaaaagcattctggtgagcaaaaacaaaatgtatatcaccagctttggcaaaggtaatagcgcagttctggaatatgaagtggataacaacgattacaatcagacccagct
gagcagcaaaggtagcagcaatattgagctgcgtggtgttaatgaagtgaacattacctttagcagcaaacacggttttgaaagcggtgttgaaatcaataccagcaatccgac
acatcgtagcggtgaaagcagtccggttcgtggtgatatgctgggcctgaaaagcgaactggaaaaacgctttttggcaaaaccttcgatgacaacattcatatccagctgatc
tataacatcctggacatcgaaaaaattctggccgtttatgtgaccaacattgtgtatgcactgaataacatgctgagcattaaagatagcgagagctatgatgatttcatgggttatc
tgagcgcacgcaatacctatgaagtttttacccatccggataaaagcaacctgagcgataaagcaaaaggcaacatcaaaaaatcctttagcaccttcaacgacctgctgaa
aaccaaacgtctgggttattttggtctggaagaaccgaaaacaaaagatacccgtgttagccaggcatacaaaaaacgtgtttatcacatgctggcaattgtgggtcagattcgt
cagagcgtgtttcatgataaaagtagcaaactggatgaggacctgtatagctttatcgatatcatcgattccgaatatcgtgaaaccctggattatctggttgatgaacgttttgaca
gcatcaacaaaggttttatccagggcaacaaagtgaatattagcctgctgatcgatatgatgaaaggctatgaagccgatgatattatccgcctgtattatgattttatcgtgctgaa
aagccagaagaatctgggctttagtatcaaaaaactgcgcgagaaaatgctggatgaatatggctttcgcttcaaagataaacagtatgatagcgtgcgtagcaagatgtataa
actgatggattttctgctgttctgcaactattatcgcaatgatgttgttgccggtgaagcactggttcgtaaactgcgttttagcatgaccgatgatgaaaaagaaggtatttacgcag
acgaagcaagcaaactgtggggtaaatttcgtaacgattttgagaacattgccgaccatatgaacggtgatgttattaaagaactgggcaaagccgatatggatttcgatgaaa
aaatactggacagcgagaaaaaaaacgcaagcgatctgctgtatttcagcaagatgatttatatgctgacctatttcctggatggcaaagaaattaatgatctgctgaccacgct
gattagcaaatttgataacatcaaagaatttctgaaaatcatgaaaagctcagccgttgatgttgaatgtgaactgaccgcaggttataaactgtttaatgatagccagcgcattac
caacgaactgttcattgttaaaaacattgcgagcatgcgtaaaccggcaagcagcgcaaaactgaccatgtttcgtgatgcactgaccattctgggtattgatgataacattaccg
atgatcgcattagcgaaatcctgaagctgaaagaaaaaggtaaaggtattcatggcctgcgcaactttattaccaataacgtgattgaaagctcccgctttgtgtacctgatcaaa
tatgcaaatgcccagaaaattcgcaaagtggcgaaaaatgaaaaggtggtgatgtttgtgttaggtggtattccggatacacagattgaacgctattacaaaagctgcgttgaatt
tccggatatgaacagcagcctggaagttaaacgtagtgaactggcacgtatgatcaaaaacatcagcttcgacgatttcaagaacgttaaacagcaggcaaaaggtcgtgaa

aatgttgcaaaagaacgtgccaaagcagtgattggtctgtatctgaccgttatgtatctgctggttaaaaatctggttaacgtgaatgcccgttatgtgattgcaattcattgtctggaa
cgtgatttcggcctgtataaagaaatcattccggaactggcaagcaagaacctgaaaaatgattatcgtattctgagtcagaccctgtgtgaactgtgtgataaaagcccgaacc
tgtttctgaagaaaaacgaacgtctgcgcaaatgcgttgaggtggatattaacaatgcagatagcagcatgacccgcaaatatcgtaattgtattgcacatctgacagttgtgcgc
gaactgaaagaatatattggtgatattcgtaccgtggacagctactttagcatctatcattatgttatgcagcggtgtattaccaaacgcgaaaatgataccaaacaagaagaga
aaatcaaatacgaggacgatctgcttaaaaaccacggctataccaaagattttgtgaaagccctgaatagcccgttcggttataacattccgcgtttcaaaaatctgagcatcga
gcagctgtttgatcgtaatgaatatctgacggaaaaaggcggtggctcgcatcatcatcatcatcactaataagatccggctgctaacaaagcccgaaaggaagctgagttgg
ctgctgccaccgctgagcaataactagcataaccccttggggcctctaaacgggtcttgaggggttttttgctgaaaggaggaactatatccggatatcccgcaagaggcccgg
cagtaccggcataaccaagcctatgcctacagcatccagggtgacggtgccgaggatgacgatgagcgcattgttagatttcatacacggtgcctgactgcgttagcaatttaa
ctgtgataaactaccgcattaaagcttatcgatgataagctgtcaaacatgagaa

Name: pET-28b-RfxCas13d-His (pET-28b-RfxCas13d-His was a gift from Ariel Bazzini & Miguel Angel Moreno-Mateos (Addgene plasmid # 141322 ; http://n2t.net/addgene:141322 ; RRID:Addgene_141322))

Map:

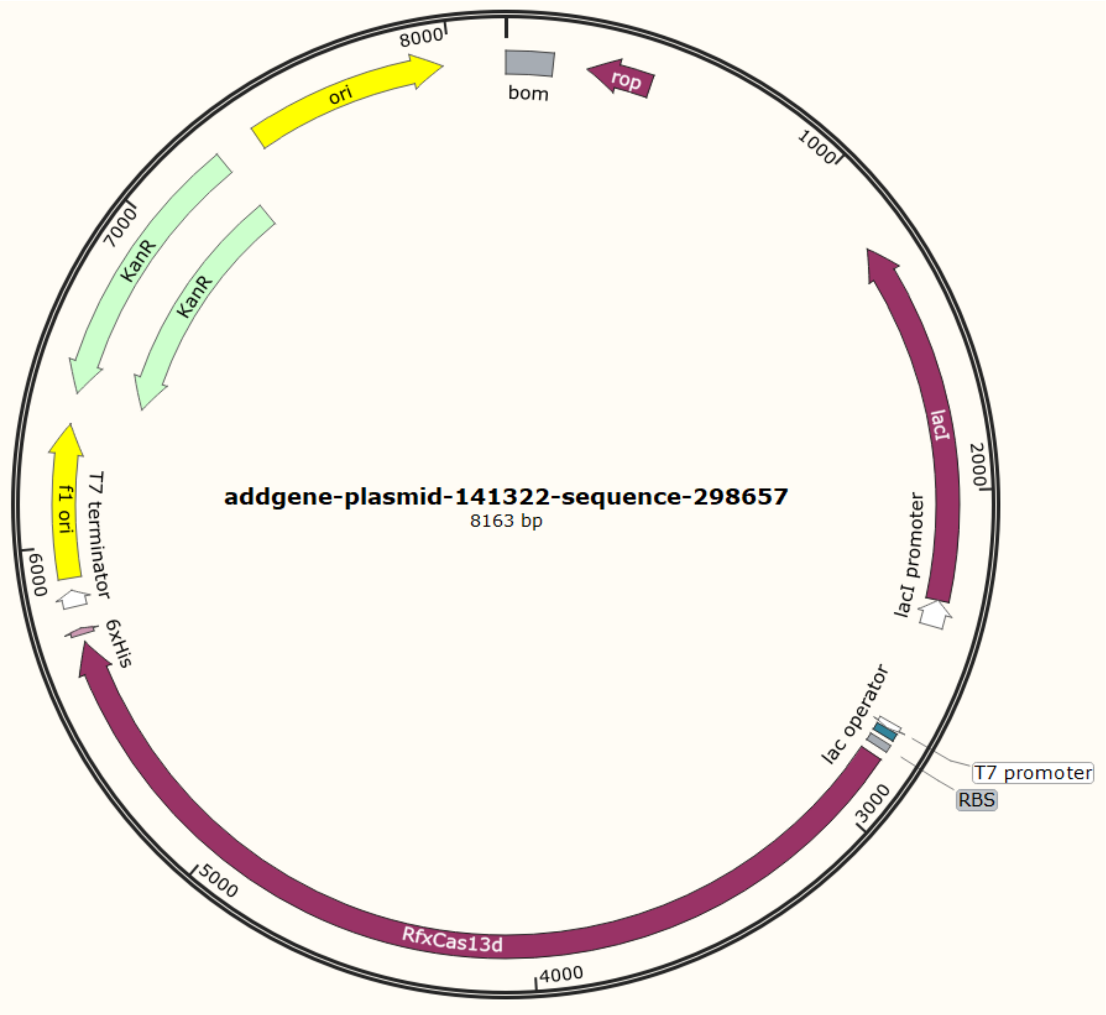

Sequence:

cctgatgcggtattttctccttacgcatctgtgcggtatttcacaccgcaatggtgcactctcagtacaatctgctctgatgccgcatagttaagccagtatacactccgctatcgctac
gtgactgggtcatggctgcgccccgacacccgccaacacccgctgacgcgccctgacgggcttgtctgctcccggcatccgcttacagacaagctgtgaccgtctccgggag
ctgcatgtgtcagaggttttcaccgtcatcaccgaaacgcgcgaggcagctgcggtaaagctcatcagcgtggtcgtgaagcgattcacagatgtctgcctgttcatccgcgtcc
agctcgttgagtttctccagaagcgttaatgtctggcttctgataaagcgggccatgttaagggcggttttttcctgtttggtcactgatgcctccgtgtaagggggatttctgttcatggg
ggtaatgataccgatgaaacgagagaggatgctcacgatacgggttactgatgatgaacatgcccggttactggaacgttgtgagggtaaacaactggcggtatggatgcgg
cgggaccagagaaaaatcactcagggtcaatgccagcgcttcgttaatacagatgtaggtgttccacagggtagccagcagcatcctgcgatgcagatccggaacataatgg

tgcagggcgctgacttccgcgtttccagactttacgaaacacggaaaccgaagaccattcatgttgttgctcaggtcgcagacgttttgcagcagcagtcgcttcacgttcgctcg
cgtatcggtgattcattctgctaaccagtaaggcaaccccgccagcctagccgggtcctcaacgacaggagcacgatcatgcgcacccgtggggccgccatgccggcgata
atggcctgcttctcgccgaaacgtttggtggcgggaccagtgacgaaggcttgagcgagggcgtgcaagattccgaataccgcaagcgacaggccgatcatcgtcgcgctcc
agcgaaagcggtcctcgccgaaaatgacccagagcgctgccggcacctgtcctacgagttgcatgataaagaagacagtcataagtgcggcgacgatagtcatgccccgc
gcccaccggaaggagctgactgggttgaaggctctcaagggcatcggtcgagatcccggtgcctaatgagtgagctaacttacattaattgcgttgcgctcactgcccgctttcc
agtcgggaaacctgtcgtgccagctgcattaatgaatcggccaacgcgcggggagaggcggtttgcgtattgggcgccagggtggtttttcttttcaccagtgagacgggcaac
agctgattgcccttcaccgcctggccctgagagagttgcagcaagcggtccacgctggtttgccccagcaggcgaaaatcctgtttgatggtggttaacggcgggatataacat
gagctgtcttcggtatcgtcgtatcccactaccgagatatccgcaccaacgcgcagcccggactcggtaatggcgcgcattgcgcccagcgccatctgatcgttggcaaccag
catcgcagtgggaacgatgccctcattcagcatttgcatggtttgttgaaaaccggacatggcactccagtcgccttcccgttccgctatcggctgaatttgattgcgagtgagatat
ttatgccagccagccagacgcagacgcgccgagacagaacttaatgggcccgctaacagcgcgatttgctggtgacccaatgcgaccagatgctccacgcccagtcgcgta
ccgtcttcatgggagaaaataatactgttgatgggtgtctggtcagagacatcaagaaataacgccggaacattagtgcaggcagcttccacagcaatggcatcctggtcatcc
agcggatagttaatgatcagcccactgacgcgttgcgcgagaagattgtgcaccgccgctttacaggcttcgacgccgcttcgttctaccatcgacaccaccacgctggcaccc
agttgatcggcgcgagatttaatcgccgcgacaatttgcgacggcgcgtgcagggccagactggaggtggcaacgccaatcagcaacgactgtttgcccgccagttgttgtgc
cacgcggttgggaatgtaattcagctccgccatcgccgcttccactttttcccgcgttttcgcagaaacgtggctggcctggttcaccacgcgggaaacggtctgataagagaca
ccggcatactctgcgacatcgtataacgttactggtttcacattcaccaccctgaattgactctcttccgggcgctatcatgccataccgcgaaaggttttgcgccattcgatggtgtc
cgggatctcgacgctctcccttatgcgactcctgcattaggaagcagcccagtagtaggttgaggccgttgagcaccgccgccgcaaggaatggtgcatgcaaggagatggc
gcccaacagtcccccggccacggggcctgccaccatacccacgccgaaacaagcgctcatgagcccgaagtggcgagcccgatcttccccatcggtgatgtcggcgatat
aggcgccagcaaccgcacctgtggcgccggtgatgccggccacgatgcgtccggcgtagaggatcgagatctcgatcccgcgaaattaatacgactcactataggggaatt
gtgagcggataacaattcccctctagaaataattttgtttaactttaagaaggagatataccatggcgagcgaggccagcatcgaaaaaaaaaagtccttcgccaagggcatg
ggcgtgaagtccacactcgtgtccggctccaaagtgtacatgacaaccttcgccgaaggcagcgacgccaggctggaaaagatcgtggagggcgacagcatcaggagcg
tgaatgagggcgaggccttcagcgctgaaatggccgataaaaacgccggctataagatcggcaacgccaaattcagccatcctaagggctacgccgtggtggctaacaac
cctctgtatacaggacccgtccagcaggatatgctcggcctgaaggaaactctggaaaagaggtacttcggcgagagcgctgatggcaatgacaatatttgtatccaggtgatc
cataacatcctggacattgaaaaaatcctcgccgaatacattaccaacgccgcctacgccgtcaacaatatctccggcctggataaggacattattggattcggcaagttctcca
cagtgtatacctacgacgaattcaaagaccccgagcaccatagggccgctttcaacaataacgataagctcatcaacgccatcaaggcccagtatgacgagttcgacaactt
cctcgataaccccagactcggctatttcggccaggcctttttcagcaaggagggcagaaattacatcatcaattacggcaacgaatgctatgacattctggccctcctgagcgga
ctgaggcactgggtggtccataacaacgaagaagagtccaggatctccaggacctggctctacaacctcgataagaacctcgacaacgaatacatctccaccctcaactacc
tctacgacaggatcaccaatgagctgaccaactccttctccaagaactccgccgccaacgtgaactatattgccgaaactctgggaatcaaccctgccgaattcgccgaacaa
tatttcagattcagcattatgaaagagcagaaaaacctcggattcaatatcaccaagctcagggaagtgatgctggacaggaaggatatgtccgagatcaggaaaaatcata
aggtgttcgactccatcaggaccaaggtctacaccatgatggactttgtgatttataggtattacatcgaagaggatgccaaggtggctgccgccaataagtccctccccgataat
gagaagtccctgagcgagaaggatatctttgtgattaacctgaggggctccttcaacgacgaccagaaggatgccctctactacgatgaagctaatagaatttggagaaagct
cgaaaatatcatgcacaacatcaaggaatttaggggaaacaagacaagagagtataagaagaaggacgcccctagactgcccagaatcctgcccgctggccgtgatgtttc
cgccttcagcaaactcatgtatgccctgaccatgttcctggatggcaaggagatcaacgacctcctgaccaccctgattaataaattcgataacatccagagcttcctgaaggtg
atgcctctcatcggagtcaacgctaagttcgtggaggaatacgcctttttcaaagactccgccaagatcgccgatgagctgaggctgatcaagtccttcgctagaatgggagaa
cctattgccgatgccaggagggccatgtatatcgacgccatccgtattttaggaaccaacctgtcctatgatgagctcaaggccctcgccgacaccttttccctggacgagaacg
gaaacaagctcaagaaaggcaagcacggcatgagaaatttcattattaataacgtgatcagcaataaaaggttccactacctgatcagatacggtgatcctgcccacctccat
gagatcgccaaaaacgaggccgtggtgaagttcgtgctcggcaggatcgctgacatccagaaaaaacagggccagaacggcaagaaccagatcgacaggtactacgaa
acttgtatcggaaaggataagggcaagagcgtgagcgaaaaggtggacgctctcacaaagatcatcaccggaatgaactacgaccaattcgacaagaaaaggagcgtca
ttgaggacaccggcagggaaaacgccgagagggagaagtttaaaaagatcatcagcctgtacctcaccgtgatctaccacatcctcaagaatattgtcaatatcaacgccag
gtacgtcatcggattccattgcgtcgagcgtgatgctcaactgtacaaggagaaaggctacgacatcaatctcaagaaactggaagagaagggattcagctccgtcaccaag
ctctgcgctggcattgatgaaactgcccccgataagagaaaggacgtggaaaaggagatggctgaaagagccaaggagagcattgacagcctcgagagcgccaacccc
aagctgtatgccaattacatcaaatacagcgacgagaagaaagccgaggagttcaccaggcagattaacagggagaaggccaaaaccgccctgaacgcctacctgagg
aacaccaagtggaatgtgatcatcagggaggacctcctgagaattgacaacaagacatgtaccctgttcagaaacaaggccgtccacctggaagtggccaggtatgtccac
gcctatatcaacgacattgccgaggtcaattcctacttccaactgtaccattacatcatgcagagaattatcatgaatgagaggtacgagaaaagcagcggaaaggtgtccga
gtacttcgacgctgtgaatgacgagaagaagtacaacgataggctcctgaaactgctgtgtgtgcctttcggctactgtatccccaggtttaagaacctgagcatcgaggccctgt
tcgataggaacgaggccgccaagttcgacaaggagaaaaagaaggtgtccggcaattccggatccggagcggccgcactcgagcaccaccaccaccaccactgagatc
cggctgctaacaaagcccgaaaggaagctgagttggctgctgccaccgctgagcaataactagcataaccccttggggcctctaaacgggtcttgaggggttttttgctgaaag
gaggaactatatccggattggcgaatgggacgcgccctgtagcggcgcattaagcgcggcgggtgtggtggttacgcgcagcgtgaccgctacacttgccagcgccctagcg
cccgctcctttcgctttcttcccttcctttctcgccacgttcgccggctttccccgtcaagctctaaatcgggggctccctttagggttccgatttagtgctttacggcacctcgaccccaa
aaaacttgattagggtgatggttcacgtagtgggccatcgccctgatagacggtttttcgccctttgacgttggagtccacgttctttaatagtggactcttgttccaaactggaacaac
actcaaccctatctcggtctattcttttgatttataagggattttgccgatttcggcctattggttaaaaaatgagctgatttaacaaaaatttaacgcgaattttaacaaaatattaacgct

tacaatttaggtggcacttttcggggaaatgtgcgcggaacccctatttgtttatttttctaaatacattcaaatatgtatccgctcatgaattaattcttagaaaaactcatcgagcatca
aatgaaactgcaatttattcatatcaggattatcaataccatatttttgaaaaagccgtttctgtaatgaaggagaaaactcaccgaggcagttccataggatggcaagatcctggt
atcggtctgcgattccgactcgtccaacatcaatacaacctattaatttcccctcgtcaaaaataaggttatcaagtgagaaatcaccatgagtgacgactgaatccggtgagaat
ggcaaaagtttatgcatttctttccagacttgttcaacaggccagccattacgctcgtcatcaaaatcactcgcatcaaccaaaccgttattcattcgtgattgcgcctgagcgagac
gaaatacgcgatcgctgttaaaaggacaattacaaacaggaatcgaatgcaaccggcgcaggaacactgccagcgcatcaacaatattttcacctgaatcaggatattcttct
aatacctggaatgctgttttcccggggatcgcagtggtgagtaaccatgcatcatcaggagtacggataaaatgcttgatggtcggaagaggcataaattccgtcagccagttta
gtctgaccatctcatctgtaacatcattggcaacgctacctttgccatgtttcagaaacaactctggcgcatcgggcttcccatacaatcgatagattgtcgcacctgattgcccgac
attatcgcgagcccatttatacccatataaatcagcatccatgttggaatttaatcgcggcctagagcaagacgtttcccgttgaatatggctcataacaccccttgtattactgtttat
gtaagcagacagttttattgttcatgaccaaaatcccttaacgtgagttttcgttccactgagcgtcagaccccgtagaaaagatcaaaggatcttcttgagatcctttttttctgcgcgt
aatctgctgcttgcaaacaaaaaaaccaccgctaccagcggtggtttgtttgccggatcaagagctaccaactctttttccgaaggtaactggcttcagcagagcgcagatacca
aatactgtccttctagtgtagccgtagttaggccaccacttcaagaactctgtagcaccgcctacatacctcgctctgctaatcctgttaccagtggctgctgccagtggcgataagt
cgtgtcttaccgggttggactcaagacgatagttaccggataaggcgcagcggtcgggctgaacggggggttcgtgcacacagcccagcttggagcgaacgacctacaccg
aactgagatacctacagcgtgagctatgagaaagcgccacgcttcccgaagggagaaaggcggacaggtatccggtaagcggcagggtcggaacaggagagcgcacg
agggagcttccagggggaaacgcctggtatctttatagtcctgtcgggtttcgccacctctgacttgagcgtcgatttttgtgatgctcgtcaggggggcggagcctatggaaaaac
gccagcaacgcggcctttttacggttcctggccttttgctggccttttgctcacatgttctttcctgcgttatcccctgattctgtggataaccgtattaccgcctttgagtgagctgataccg
ctcgccgcagccgaacgaccgagcgcagcgagtcagtgagcgaggaagcggaagagcg

Name: pEF1-a_LbuCas13a-HA-IRES-BFP-2A-BSD (Addgene ID = 251219)

Map:

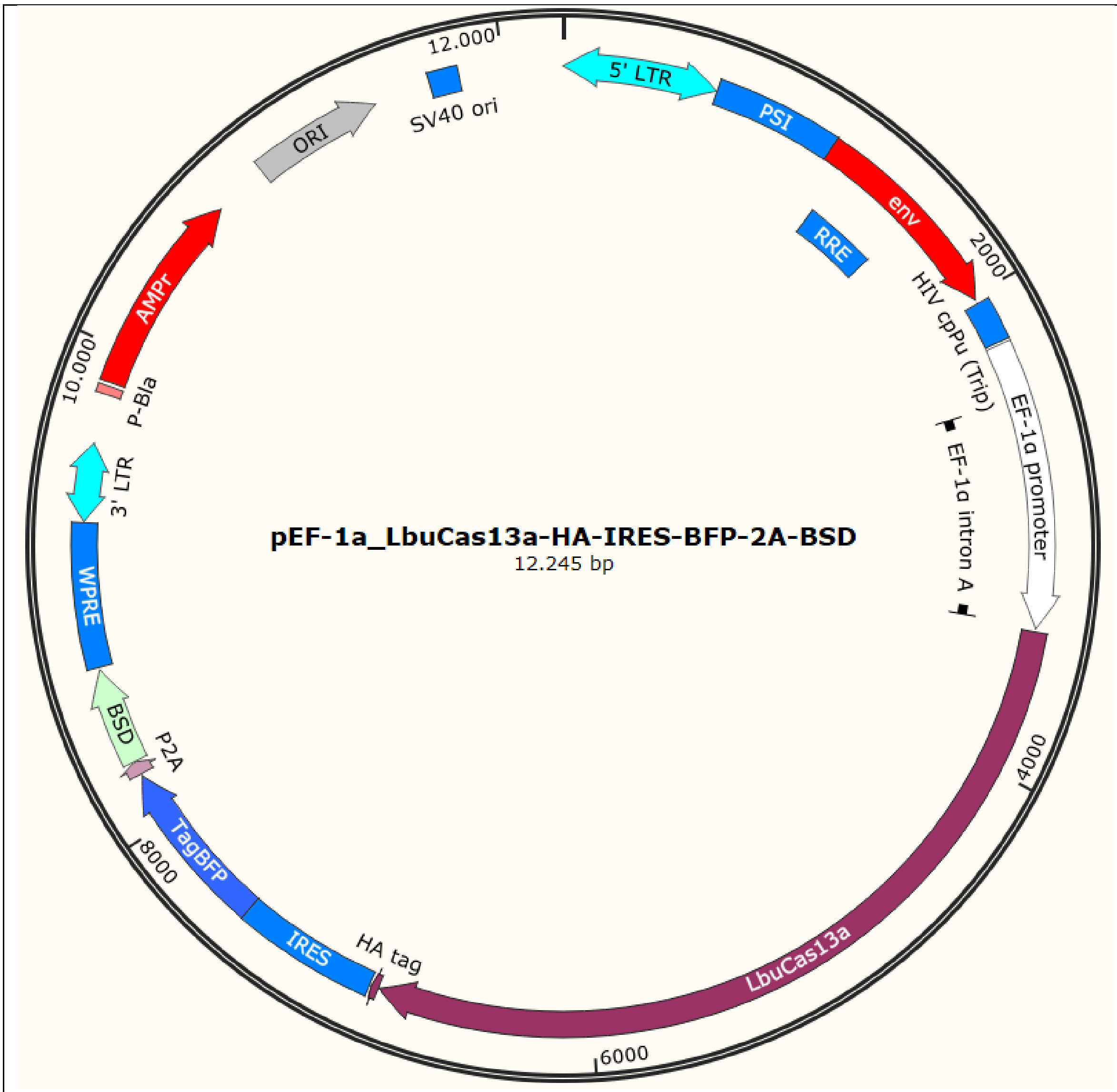

Sequence:

tggaagggctaattcactcccaaagaagacaagatatccttgatctgtggatctaccacacacaaggctacttccctgattagcagaactacacaccagggccaggggtcag
atatccactgacctttggatggtgctacaagctagtaccagttgagccagataaggtagaagaggccaataaaggagagaacaccagcttgttacaccctgtgagcctgcatg
ggatggatgacccggagagagaagtgttagagtggaggtttgacagccgcctagcatttcatcacgtggcccgagagctgcatccggagtacttcaagaactgctgatatcga
gcttgctacaagggactttccgctggggactttccagggaggcgtggcctgggcgggactggggagtggcgagccctcagatcctgcatataagcagctgctttttgcctgtact
gggtctctctggttagaccagatctgagcctgggagctctctggctaactagggaacccactgcttaagcctcaataaagcttgccttgagtgcttcaagtagtgtgtgcccgtctgt
tgtgtgactctggtaactagagatccctcagacccttttagtcagtgtggaaaatctctagcagtggcgcccgaacagggacttgaaagcgaaagggaaaccagaggagctct
ctcgacgcaggactcggcttgctgaagcgcgcacggcaagaggcgaggggcggcgactggtgagtacgccaaaaattttgactagcggaggctagaaggagagagatg
ggtgcgagagcgtcagtattaagcgggggagaattagatcgcgatgggaaaaaattcggttaaggccagggggaaagaaaaaatataaattaaaacatatagtatgggca
agcagggagctagaacgattcgcagttaatcctggcctgttagaaacatcagaaggctgtagacaaatactgggacagctacaaccatcccttcagacaggatcagaagaa
cttagatcattatataatacagtagcaaccctctattgtgtgcatcaaaggatagagataaaagacaccaaggaagctttagacaagatagaggaagagcaaaacaaaagta
agaccaccgcacagcaagcggccggccgctgatcttcagacctggaggaggagatatgagggacaattggagaagtgaattatataaatataaagtagtaaaaattgaac
cattaggagtagcacccaccaaggcaaagagaagagtggtgcagagagaaaaaagagcagtgggaataggagctttgttccttgggttcttgggagcagcaggaagcact
atgggcgcagcgtcaatgacgctgacggtacaggccagacaattattgtctggtatagtgcagcagcagaacaatttgctgagggctattgaggcgcaacagcatctgttgca
actcacagtctggggcatcaagcagctccaggcaagaatcctggctgtggaaagatacctaaaggatcaacagctcctggggatttggggttgctctggaaaactcatttgcac
cactgctgtgccttggaatgctagttggagtaataaatctctggaacagatttggaatcacacgacctggatggagtgggacagagaaattaacaattacacaagcttaatacac
tccttaattgaagaatcgcaaaaccagcaagaaaagaatgaacaagaattattggaattagataaatgggcaagtttgtggaattggtttaacataacaaattggctgtggtatat

aaaattattcataatgatagtaggaggcttggtaggtttaagaatagtttttgctgtactttctatagtgaatagagttaggcagggatattcaccattatcgtttcagacccacctccca
accccgaggggacccgacaggcccgaaggaatagaagaagaaggtggagagagagacagagacagatccattcgattagtgaacggatctcgacggtatcgccgaatt
cacaaatggcagtattcatccacaattttaaaagaaaaggggggattggggggtacagtgcaggggaaagaatagtagacataatagcaacagacatacaaactaaaga
attacaaaaacaaattacaaaaattcaaaattttcgggtttattacagggacagcagagatccagtttggactagtcgtgaggctccggtgcccgtcagtgggcagagcgcaca
tcgcccacagtccccgagaagttggggggaggggtcggcaattgaaccggtgcctagagaaggtggcgcggggtaaactgggaaagtgatgtcgtgtactggctccgccttt
ttcccgagggtgggggagaaccgtatataagtgcagtagtcgccgtgaacgttctttttcgcaacgggtttgccgccagaacacaggtaagtgccgtgtgtggttcccgcgggcc
tggcctctttacgggttatggcccttgcgtgccttgaattacttccacctggctgcagtacgtgattcttgatcccgagcttcgggttggaagtgggtgggagagttcgaggccttgcg
cttaaggagccccttcgcctcgtgcttgagttgaggcctggcctgggcgctggggccgccgcgtgcgaatctggtggcaccttcgcgcctgtctcgctgctttcgataagtctctag
ccatttaaaatttttgatgacctgctgcgacgctttttttctggcaagatagtcttgtaaatgcgggccaagatctgcacactggtatttcggtttttggggccgcgggcggcgacggg
gcccgtgcgtcccagcgcacatgttcggcgaggcggggcctgcgagcgcggccaccgagaatcggacgggggtagtctcaagctggccggcctgctctggtgcctggcctc
gcgccgccgtgtatcgccccgccctgggcggcaaggctggcccggtcggcaccagttgcgtgagcggaaagatggccgcttcccggccctgctgcagggagctcaaaatg
gaggacgcggcgctcgggagagcgggcgggtgagtcacccacacaaaggaaaagggcctttccgtcctcagccgtcgcttcatgtgactccacggagtaccgggcgccgt
ccaggcacctcgattagttctcgagcttttggagtacgtcgtctttaggttggggggaggggttttatgcgatggagtttccccacactgagtgggtggagactgaagttaggccag
cttggcacttgatgtaattctccttggaatttgccctttttgagtttggatcttggttcattctcaagcctcagacagtggttcaaagtttttttcttccatttcaggtgtcgtgaagcggccgcc
accatgaaggtaaccaaagtaggaggtatctcacataagaagtataccagtgaagggagacttgtaaagagtgaatcagaggagaatcgcacagacgaacgcttgtctgct
ctgctcaacatgcgcctggatatgtatataaagaacccttcctccacggagacaaaggagaaccagaagaggattgggaagttgaagaagttcttctccaataaaatggtgtat
ctgaaagacaatacccttcccttaagaacggcaaaaaagagaatattgacagagagtactcagagactgacatacttgagtcagacgtacgcgacaaaaagaacttcgca
gttctgaagagaaaatttacctgaatgaaaacgtgaatagcgaagaactggaggtgtttagaaacgatatcaagaaaaaactcaataaaatcaactcactgaaatacagttttga
gaagaataaagctaactaccagaaaatcaacgagaacaatatcgagaaggtggaaggtaagtccaaaagaaatataatttacgactattacagagaatctgcgaaaagg
gacgcgtacgtatccaatgttaaggaggcatttgataagctttacaaagaagaagatattgctaagttggtacttgaaatagaaaacctcactaaactcgagaaatacaaaatc
cgagagttttaccacgagataattggccgaaaaaatgataaagagaattttgctaagattatatacgaggaaatacaaaatgtaaacaacatgaaggagcttatagaaaaggt
cccggatatgtctgaactgaaaaaatcacaagtttttacaaatactacctcgacaaggaggagctgaacgacaagaatatcaaatatgccttctgccactttgtcgaaattgag
atgagtcaactgctcaagaactatgtgtacaagcgactctccaacattagcaatgacaaaatcaaacgaattttcgaataccagaatttgaagaagttgatagaaaacaagctg
cttaataaactcgatacttacgttcggaactgtgggaaatataactattaccttcaggatggcgaaatagccacgtcagactttattgcacggaacaggcaaaatgaggcctttct
gaggaatattatcggagtcagcagtgtcgcttattttagtctgcggaacatcctggagacagagaacgaaaacgatataactggacgcatgcgcggaaagacggtaaaaaat
aataaaggtgaagaaaagtacgttagtggggaggtagataagatttataatgaaaataagaagaacgaagttaaagagaacttgaagatgttctacagttatgatttcaatatg
gacaacaagaacgaaatcgaagatttttttgctaatattgacgaagctatctccagcatcaggcatggtatagtgcacttcaatcttgagttggaaggcaaggacatttttgcgttca
agaacatcgccccaagcgaaataagcaaaaagatgttccagaacgagataaatgagaagaaattgaaattgaaaatcttccggcaattgaactcagccaacgttttcagata
cctcgaaaagtataagatcctcaattacttgaaacgcacacgattcgaatttgttaacaagaatattccgttcgtccctagctttaccaagctttatagccgcatcgatgatctgaag
aattcccttgggatatactggaagacacctaaaactaatgacgataataaaacgaaggagataatcgatgcacagatatacttgttgaaaaatatatactacggcgagtttctta
attatttcatgtcaaataatgggaatttttttgaaatttcaaaagaaattatagaactgaataagaacgacaagcgcaaccttaagactggcttttacaaacttcagaaatttgagga
catccaagaaaagatcccgaaggagtatttggcgaacattcaaagcctttacatgataaatgccgggaaccaggatgaagaagaaaaagacacctatattgattttatccaga
aaatctttttgaagggttttatgacttacctcgcgaacaatggaagactcagcttgatctatataggcagtgacgaggagactaacacgagtttggccgaaaagaagcaggagtt
tgataaattccttaaaaagtatgaacaaaataataacattaagataccatacgaaataaacgagtttcttcgcgaaatcaaattgggaaacatccttaagtatacggagaggctt
aacatgttttaccttatcctcaaactcctcaaccacaaggagctcaccaacctgaaagggagcctcgaaaagtaccagtccgcgaataaagaggaggccttctccgaccaac
ttgaacttattaatcttctgaacctggacaataatagagtcacggaagattttgagttggaggcggatgaaattggcaaattccttgattttaatggcaataaagtcaaagacaaca
aagaattgaagaaattcgacactaacaagatatattttgacggggagaatatcatcaaacatagagcccttttacaacatcaaaaaatatgggatgctcaatttgcttgaaaaaat
agctgataaggccgggtacaagatctcaatcgaagaactgaaaaaatactctaacaagaagaatgagattgagaaaaatcacaagatgcaagaaaacctccaccgcaag
tatgcgcggcctcgcaaggacgagaagttcacagatgaagactacgagtcttacaagcaggccatcgaaaatatcgaggaatatactcacctcaaaaacaaggtagaattt
aatgaactcaatctgctgcaagggctgctccttcgcatccttcacaggctcgtgggatacacgtccatttgggaacgcgatttgcggtttcggctcaaggggggaatttccggagaa
tcagtatatcgaagaaatattcaacttcgagaacaaaaagaatgtgaagtataagggtggacagatcgtagaaaagtatattaagttttacaaggaactgcaccagaacgatg
aggtcaagatcaataaatacagctccgctaatatcaaggtgctcaaacaggaaaaaaaggacttgtacattcgaaactatatagcgcactttaactatatcccccatgctgaaa
tatcactccttgaagtcttggaaaaacctcagaaaactttgagctacgatcgaaagctgaagaacgcagtcatgaaatctgttgttgacatactcaaggagtatggatttgtcgcaa
cgtttaagatcggggcggacaaaaagatcggaattcagacgctcgaatcagaaaagattgtgcatctgaagaacctcaaaaagaaaaagttgatgacagacagacaggaactcc
gaagaattgtgcaagctcgtgaagattatgtttgagtataaaatggaagaaaagaaaagtgaaaacggatacccttatgacgtacctgactatgcttaaggatccctcccccc
ccctaacgttactggccgaagccgcttggaataaggccggtgtgcgtttgtctatatgttattttccaccatattgccgtcttttggcaatgtgagggcccggaaacctggccctgtctt
cttgacgagcattcctaggggtctttcccctctcgccaaaggaatgcaaggtctgttgaatgtcgtgaaggaagcagttcctctggaagcttcttgaagacaaacaacgtctgtag
cgaccctttgcaggcagcggaaccccccacctggcgacaggtgcctctgcggccaaaagccacgtgtataagatacacctgcaaaggcggcacaaccccagtgccacgtt
gtgagttggatagttgtggaaagagtcaaatggctctcctcaagcgtattcaacaaggggctgaaggatgcccagaaggtaccccattgtatgggatctgatctggggcctcggt
gcacatgctttacatgtgtttagtcgaggttaaaaaaacgtctaggccccccgaaccacggggacgtggttttcctttgaaaaacacgatgataatatggccacacatatgagcg

agctgattaaggagaacatgcacatgaagctgtacatggagggcaccgtggacaaccatcacttcaagtgcacatccgagggcgaaggcaagccctacgagggcaccca
gaccatgagaatcaaggtggtcgagggcggccctctccccttcgccttcgacatcctggctactagcttcctctacggcagcaagaccttcatcaaccacacccagggcatccc
cgacttcttcaagcagtccttccctgagggcttcacatgggagagagtcaccacatacgaagacgggggcgtgctgaccgctacccaggacaccagcctccaggacggctg
cctcatctacaacgtcaagatcagaggggtgaacttcacatccaacggccctgtgatgcagaagaaaacactcggctgggaggccttcaccgagacgctgtaccccgctga
cggcggcctggaaggcagaaacgacatggccctgaagctcgtgggcgggagccatctgatcgcaaacatcaagaccacatatagatccaagaaacccgctaagaacctc
aagatgcctggcgtctactatgtggactacagactggaaagaatcaaggaggccaacaacgagacctacgtcgagcagcacgaggtggcagtggccagatactgcgacct
ccctagcaaactggggcacaagcttaatggatccggcgcaacaaacttctctctgctgaaacaagccggagatgtcgaagagaatcctggaccgatggccaagcctttgtctc
aagaagaatccaccctcattgaaagagcaacggctacaatcaacagcatccccatctctgaagactacagcgtcgccagcgcagctctctctagcgacggccgcatcttcac
tggtgtcaatgtatatcattttactgggggaccttgtgcagaactcgtggtgctgggcactgctgctgctgcggcagctggcaacctgacttgtatcgtcgcgatcggaaatgagaa
caggggcatcttgagcccctgcggacggtgccgacaggtgcttctcgatctgcatcctgggatcaaagccatagtgaaggacagtgatggacagccgacggcagttgggatt
cgtgaattgctgccctctggttatgtgtgggagggctaagatcgatagatcctaatcaacctctggattacaaaatttgtgaaagattgactggtattcttaactatgttgctccttttacg
ctatgtggatacgctgctttaatgcctttgtatcatgctattgcttcccgtatggctttcattttctcctccttgtataaatcctggttgctgtctctttatgaggagttgtggcccgttgtcaggc
aacgtggcgtggtgtgcactgtgtttgctgacgcaacccccactggttggggcattgccaccacctgtcagctcctttccgggactttcgctttccccctccctattgccacggcgga
actcatcgccgcctgccttgcccgctgctggacaggggctcggctgttgggcactgacaattccgtggtgttgtcggggaaatcatcgtcctttccttggctgctcgcctgtgttgcca
cctggattctgcgcgggacgtccttctgctacgtcccttcggccctcaatccagcggaccttccttcccgcggcctgctgccggctctgcggcctcttccgcgtcttcgccttcgccct
cagacgagtcggatctccctttgggccgcctccccgcctggtacctttaagaccaatgacttacaaggcagctgtagatcttagccactttttaaaagaaaaggggggactgga
agggctaattcactcccaacgaagacaagatcacctgcaggacaggcgcgccctgctttttgcttgtactgggtctctctggttagaccagatctgagcctgggagctctctggct
aactagggaacccactgcttaagcctcaataaagcttgccttgagtgcttcaagtagtgtgtgcccgtctgttgtgtgactctggtaactagagatccctcagacccttttagtcagtg
tggaaaatctctagcacccgggcgattaaggaaagggctagatcattcttgaagacgaaagggcctcgtgatacgcctatttttataggttaatgtcatgataataatggtttcttag
acgtcaggtggcacttttcggggaaatgtgcgcggaacccctatttgtttatttttctaaatacattcaaatatgtatccgctcatgagacaataaccctgataaatgcttcaataatatt
gaaaaaggaagagtatgagtattcaacatttccgtgtcgcccttattcccttttttgcggcattttgccttcctgtttttgctcacccagaaacgctggtgaaagtaaaagatgctgaag
atcagttgggtgcacgagtgggttacatcgaactggatctcaacagcggtaagatccttgagagttttcgccccgaagaacgttttccaatgatgagcacttttaaagttctgctatg
tggcgcggtattatcccgtgttgacgccgggcaagagcaactcggtcgccgcatacactattctcagaatgacttggttgagtactcaccagtcacagaaaagcatcttacggat
ggcatgacagtaagagaattatgcagtgctgccataaccatgagtgataacactgcggccaacttacttctgacaacgatcggaggaccgaaggagctaaccgcttttttgcac
aacatgggggatcatgtaactcgccttgatcgttgggaaccggagctgaatgaagccataccaaacgacgagcgtgacaccacgatgcctgtagcaatggcaacaacgttg
cgcaaactattaactggcgaactacttactctagcttcccggcaacaattaatagactggatggaggcggataaagttgcaggaccacttctgcgctcggcccttccggctggct
ggtttattgctgataaatctggagccggtgagcgtgggtctcgcggtatcattgcagcactggggccagatggtaagccctcccgtatcgtagttatctacacgacggggagtcag
gcaactatggatgaacgaaatagacagatcgctgagataggtgcctcactgattaagcattggtaactgtcagaccaagtttactcatatatactttagattgatttaaaacttcattt
ttaatttaaaaggatctaggtgaagatcctttttgataatctcatgaccaaaatcccttaacgtgagttttcgttccactgagcgtcagaccccgtagaaaagatcaaaggatcttctt
gagatcctttttttctgcgcgtaatctgctgcttgcaaacaaaaaaaccaccgctaccagcggtggtttgtttgccggatcaagagctaccaactctttttccgaaggtaactggcttc
agcagagcgcagataccaaatactgttcttctagtgtagccgtagttaggccaccacttcaagaactctgtagcaccgcctacatacctcgctctgctaatcctgttaccagtggct
gctgccagtggcgataagtcgtgtcttaccgggttggactcaagacgatagttaccggataaggcgcagcggtcgggctgaacggggggttcgtgcacacagcccagcttgg
agcgaacgacctacaccgaactgagatacctacagcgtgagctatgagaaagcgccacgcttcccgaagggagaaaggcggacaggtatccggtaagcggcagggtcg
gaacaggagagcgcacgagggagcttccagggggaaacgcctggtatctttatagtcctgtcgggtttcgccacctctgacttgagcgtcgatttttgtgatgctcgtcaggggggg
cggagcctatggaaaaacgccagcaacgcggcctttttacggttcctggccttttgctggccttttgctcacatgttctttcctgcgttatcccctgattctgtggataaccgtattaccgc
ctttgagtgagctgataccgctcgccgcagccgaacgaccgagcgcagcgagtcagtgagcgaggaagcggaagagcgcccaatacgcaaaccgcctctccccgcgcgt
tggccgattcattaatgcagcaagctcatggctgactaattttttttatttatgcagaggccgaggccgcctcggcctctgagctattccagaagtagtgaggaggcttttttggaggc
ctaggcttttgcaaaaagctccccgtggcacgacaggtttcccgactggaaagcgggcagtgagcgcaacgcaattaatgtgagttagctcactcattaggcaccccaggcttt
acactttatgcttccggctcgtatgttgtgtggaattgtgagcggataacaatttcacacaggaaacagctatgacatgattacgaatttcacaaataaagcatttttttcactgcattct
agttgtggtttgtccaaactcatcaatgtatcttatcatgtctggatcaactggataactcaagctaaccaaaatcatcccaaacttcccaccccataccctattaccactgccaatta
cctgtggtttcatttactctaaacctgtgattcctctgaattattttcattttaaagaaattgtatttgttaaatatgtactacaaacttagtagt

Name: pEF-1a_RfxCas13d-HA-IRES-BFP-2A-BSD (Addgene ID = 251220)

Map:

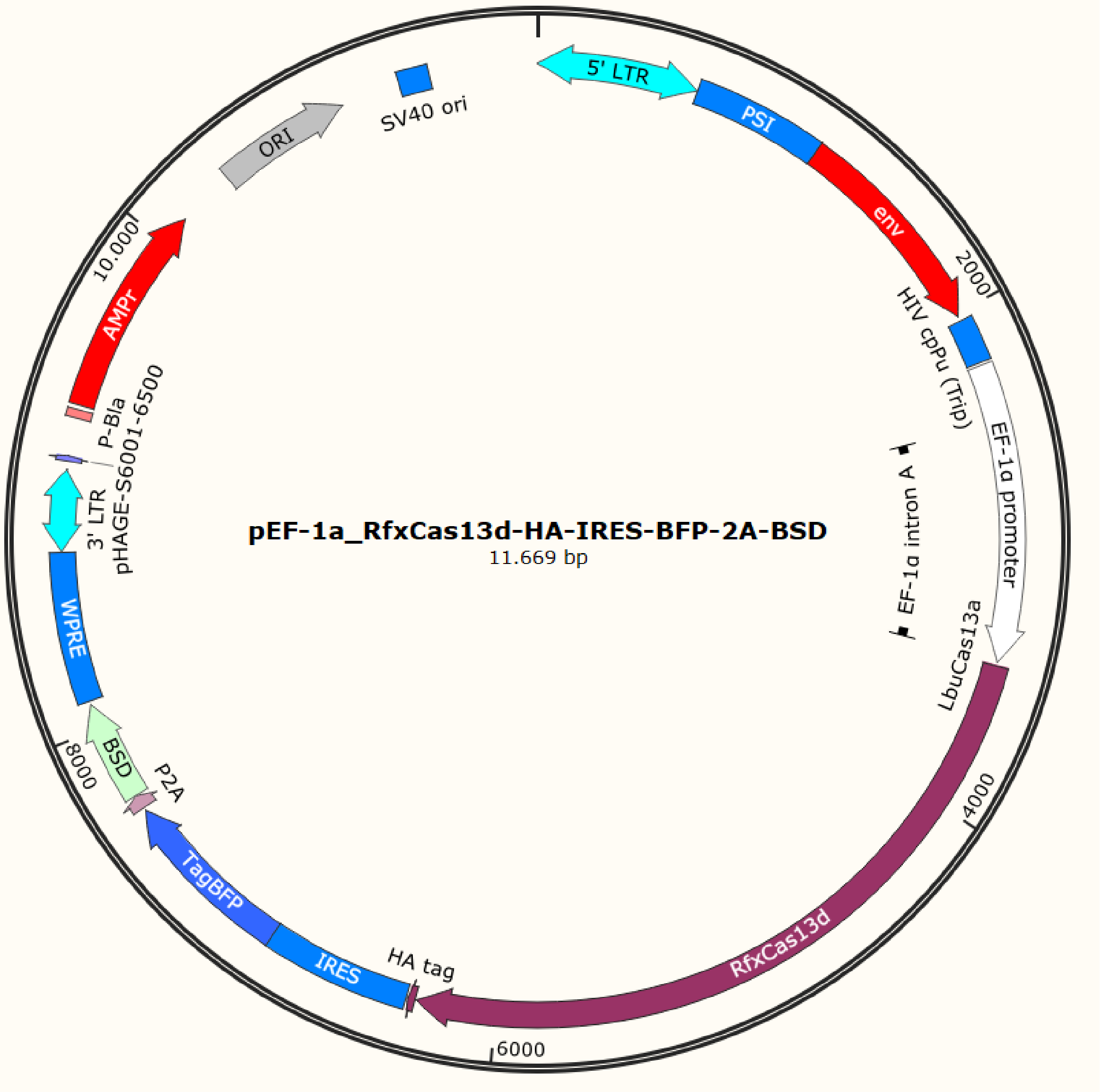

Sequence:

tggaagggctaattcactcccaaagaagacaagatatccttgatctgtggatctaccacacacaaggctacttccctgattagcagaactacacaccagggccaggggtcag
atatccactgacctttggatggtgctacaagctagtaccagttgagccagataaggtagaagaggccaataaaggagagaacaccagcttgttacaccctgtgagcctgcatg
ggatggatgacccggagagagaagtgttagagtggaggtttgacagccgcctagcatttcatcacgtggcccgagagctgcatccggagtacttcaagaactgctgatatcga
gcttgctacaagggactttccgctggggactttccagggaggcgtggcctgggcgggactggggagtggcgagccctcagatcctgcatataagcagctgctttttgcctgtact
gggtctctctggttagaccagatctgagcctgggagctctctggctaactagggaacccactgcttaagcctcaataaagcttgccttgagtgcttcaagtagtgtgtgcccgtctgt
tgtgtgactctggtaactagagatccctcagacccttttagtcagtgtggaaaatctctagcagtggcgcccgaacagggacttgaaagcgaaagggaaaccagaggagctct
ctcgacgcaggactcggcttgctgaagcgcgcacggcaagaggcgaggggcggcgactggtgagtacgccaaaaattttgactagcggaggctagaaggagagagatg
ggtgcgagagcgtcagtattaagcgggggagaattagatcgcgatgggaaaaaattcggttaaggccagggggaaagaaaaaatataaattaaaacatatagtatgggca
agcagggagctagaacgattcgcagttaatcctggcctgttagaaacatcagaaggctgtagacaaatactgggacagctacaaccatcccttcagacaggatcagaagaa
cttagatcattatataatacagtagcaaccctctattgtgtgcatcaaaggatagagataaaagacaccaaggaagctttagacaagatagaggaagagcaaaacaaaagta
agaccaccgcacagcaagcggccggccgctgatcttcagacctggaggaggagatatgagggacaattggagaagtgaattatataaatataaagtagtaaaaattgaac
cattaggagtagcacccaccaaggcaaagagaagagtggtgcagagagaaaaaagagcagtgggaataggagctttgttccttgggttcttgggagcagcaggaagcact
atgggcgcagcgtcaatgacgctgacggtacaggccagacaattattgtctggtatagtgcagcagcagaacaatttgctgagggctattgaggcgcaacagcatctgttgca
actcacagtctggggcatcaagcagctccaggcaagaatcctggctgtggaaagatacctaaaggatcaacagctcctggggatttggggttgctctggaaaactcatttgcac
cactgctgtgccttggaatgctagttggagtaataaatctctggaacagatttggaatcacacgacctggatggagtgggacagagaaattaacaattacacaagcttaatacac
tccttaattgaagaatcgcaaaaccagcaagaaaagaatgaacaagaattattggaattagataaatgggcaagtttgtggaattggtttaacataacaaattggctgtggtatat

aaaattattcataatgatagtaggaggcttggtaggtttaagaatagtttttgctgtactttctatagtgaatagagttaggcagggatattcaccattatcgtttcagacccacctccca
accccgaggggacccgacaggcccgaaggaatagaagaagaaggtggagagagagacagagacagatccattcgattagtgaacggatctcgacggtatcgccgaatt
cacaaatggcagtattcatccacaattttaaaagaaaaggggggattggggggtacagtgcaggggaaagaatagtagacataatagcaacagacatacaaactaaaga
attacaaaaacaaattacaaaaattcaaaattttcgggtttattacagggacagcagagatccagtttggactagtcgtgaggctccggtgcccgtcagtgggcagagcgcaca
tcgcccacagtccccgagaagttggggggaggggtcggcaattgaaccggtgcctagagaaggtggcgcggggtaaactgggaaagtgatgtcgtgtactggctccgccttt
ttcccgagggtgggggagaaccgtatataagtgcagtagtcgccgtgaacgttctttttcgcaacgggtttgccgccagaacacaggtaagtgccgtgtgtggttcccgcgggcc
tggcctctttacgggttatggcccttgcgtgccttgaattacttccacctggctgcagtacgtgattcttgatcccgagcttcgggttggaagtgggtgggagagttcgaggccttgcg
cttaaggagccccttcgcctcgtgcttgagttgaggcctggcctgggcgctggggccgccgcgtgcgaatctggtggcaccttcgcgcctgtctcgctgctttcgataagtctctag
ccatttaaaatttttgatgacctgctgcgacgctttttttctggcaagatagtcttgtaaatgcgggccaagatctgcacactggtatttcggtttttggggccgcgggcggcgacggg
gcccgtgcgtcccagcgcacatgttcggcgaggcggggcctgcgagcgcggccaccgagaatcggacgggggtagtctcaagctggccggcctgctctggtgcctggcctc
gcgccgccgtgtatcgccccgccctgggcggcaaggctggcccggtcggcaccagttgcgtgagcggaaagatggccgcttcccggccctgctgcagggagctcaaaatg
gaggacgcggcgctcgggagagcgggcgggtgagtcacccacacaaaggaaaagggcctttccgtcctcagccgtcgcttcatgtgactccacggagtaccgggcgccgt
ccaggcacctcgattagttctcgagcttttggagtacgtcgtctttaggttggggggaggggttttatgcgatggagtttccccacactgagtgggtggagactgaagttaggccag
cttggcacttgatgtaattctccttggaatttgccctttttgagtttggatcttggttcattctcaagcctcagacagtggttcaaagtttttttcttccatttcaggtgtcgtgaagcggccgcc
accatgatcgaaaaaaaaaagtccttcgccaagggcatgggcgtgaagtccacactcgtgtccggctccaaagtgtacatgacaaccttcgccgaaggcagcgacgccag
gctggaaaagatcgtggagggcgacagcatcaggagcgtgaatgagggcgaggccttcagcgctgaaatggccgataaaaacgccggctataagatcggcaacgccaa
attcagccatcctaagggctacgccgtggtggctaacaaccctctgtatacaggacccgtccagcaggatatgctcggcctgaaggaaactctggaaaagaggtacttcggcg
agagcgctgatggcaatgacaatatttgtatccaggtgatccataacatcctggacattgaaaaaatcctcgccgaatacattaccaacgccgcctacgccgtcaacaatatctc
cggcctggataaggacattattggattcggcaagttctccacagtgtatacctacgacgaattcaaagaccccgagcaccatagggccgctttcaacaataacgataagctcat
caacgccatcaaggcccagtatgacgagttcgacaacttcctcgataaccccagactcggctatttcggccaggcctttttcagcaaggagggcagaaattacatcatcaatta
cggcaacgaatgctatgacattctggccctcctgagcggactgaggcactgggtggtccataacaacgaagaagagtccaggatctccaggacctggctctacaacctcgat
aagaacctcgacaacgaatacatctccaccctcaactacctctacgacaggatcaccaatgagctgaccaactccttctccaagaactccgccgccaacgtgaactatattgc
cgaaactctgggaatcaaccctgccgaattcgccgaacaatatttcagattcagcattatgaaagagcagaaaaacctcggattcaatatcaccaagctcagggaagtgatgc
tggacaggaaggatatgtccgagatcaggaaaaatcataaggtgttcgactccatcaggaccaaggtctacaccatgatggactttgtgatttataggtattacatcgaagagg
atgccaaggtggctgccgccaataagtccctccccgataatgagaagtccctgagcgagaaggatatctttgtgattaacctgaggggctccttcaacgacgaccagaaggat
gccctctactacgatgaagctaatagaatttggagaaagctcgaaaatatcatgcacaacatcaaggaatttaggggaaacaagacaagagagtataagaagaaggacgc
ccctagactgcccagaatcctgcccgctggccgtgatgtttccgccttcagcaaactcatgtatgccctgaccatgttcctggatggcaaggagatcaacgacctcctgaccacc
ctgattaataaattcgataacatccagagcttcctgaaggtgatgcctctcatcggagtcaacgctaagttcgtggaggaatacgcctttttcaaagactccgccaagatcgccga
tgagctgaggctgatcaagtccttcgctagaatgggagaacctattgccgatgccaggagggccatgtatatcgacgccatccgtattttaggaaccaacctgtcctatgatgag
ctcaaggccctcgccgacaccttttccctggacgagaacggaaacaagctcaagaaaggcaagcacggcatgagaaatttcattattaataacgtgatcagcaataaaaggt
tccactacctgatcagatacggtgatcctgcccacctccatgagatcgccaaaaacgaggccgtggtgaagttcgtgctcggcaggatcgctgacatccagaaaaaacaggg
ccagaacggcaagaaccagatcgacaggtactacgaaacttgtatcggaaaggataagggcaagagcgtgagcgaaaaggtggacgctctcacaaagatcatcaccgg
aatgaactacgaccaattcgacaagaaaaggagcgtcattgaggacaccggcagggaaaacgccgagagggagaagtttaaaaagatcatcagcctgtacctcaccgtg
atctaccacatcctcaagaatattgtcaatatcaacgccaggtacgtcatcggattccattgcgtcgagcgtgatgctcaactgtacaaggagaaaggctacgacatcaatctca
agaaactggaagagaagggattcagctccgtcaccaagctctgcgctggcattgatgaaactgcccccgataagagaaaggacgtggaaaaggagatggctgaaagagc
caaggagagcattgacagcctcgagagcgccaaccccaagctgtatgccaattacatcaaatacagcgacgagaagaaagccgaggagttcaccaggcagattaacag
ggagaaggccaaaaccgccctgaacgcctacctgaggaacaccaagtggaatgtgatcatcagggaggacctcctgagaattgacaacaagacatgtaccctgttcagaa
acaaggccgtccacctggaagtggccaggtatgtccacgcctatatcaacgacattgccgaggtcaattcctacttccaactgtaccattacatcatgcagagaattatcatgaat
gagaggtacgagaaaagcagcggaaaggtgtccgagtacttcgacgctgtgaatgacgagaagaagtacaacgataggctcctgaaactgctgtgtgtgcctttcggctact
gtatccccaggtttaagaacctgagcatcgaggccctgttcgataggaacgaggccgccaagttcgacaaggagaaaaagaaggtgtccggcaattccggataccccttatga
cgtacctgactatgcttaaggatccctcccccccccctaacgttactggccgaagccgcttggaataaggccggtgtgcgtttgtctatatgttattttccaccatattgccgtcttttgg
caatgtgagggcccggaaacctggccctgtcttcttgacgagcattcctaggggtctttcccctctcgccaaaggaatgcaaggtctgttgaatgtcgtgaaggaagcagttcctct
ggaagcttcttgaagacaaacaacgtctgtagcgaccctttgcaggcagcggaaccccccacctggcgacaggtgcctctgcggccaaaagccacgtgtataagatacacc
tgcaaaggcggcacaaccccagtgccacgttgtgagttggatagttgtggaaagagtcaaatggctctcctcaagcgtattcaacaaggggctgaaggatgcccagaaggta
ccccattgtatgggatctgatctggggcctcggtgcacatgctttacatgtgtttagtcgaggttaaaaaaacgtctaggccccccgaaccacggggacgtggttttcctttgaaaa
acacgatgataatatggccacacatatgagcgagctgattaaggagaacatgcacatgaagctgtacatggagggcaccgtggacaaccatcacttcaagtgcacatccga
gggcgaaggcaagccctacgagggcacccagaccatgagaatcaaggtggtcgagggcggccctctcccctttcgccttcgacatcctggctactagcttcctctacggcagc
aagaccttcatcaaccacacccagggcatccccgacttcttcaagcagtccttccctgagggcttcacatgggagagagtcaccacatacgaagacgggggcgtgctgaccg
ctacccaggacaccagcctccaggacggctgcctcatctacaacgtcaagatcagaggggtgaacttcacatccaacggccctgtgatgcagaagaaaacactcggctgg
gaggccttcaccgagacgctgtaccccgctgacggcggcctggaaggcagaaacgacatggccctgaagctcgtgggcgggagccatctgatcgcaaacatcaagacca

catatagatccaagaaacccgctaagaacctcaagatgcctggcgtctactatgtggactacagactggaaagaatcaaggaggccaacaacgagacctacgtcgagcag
cacgaggtggcagtggccagatactgcgacctccctagcaaactggggcacaagcttaatggatccggcgcaacaaacttctctctgctgaaacaagccggagatgtcgaa
gagaatcctggaccgatggccaagcctttgtctcaagaagaatccaccctcattgaaagagcaacggctacaatcaacagcatccccatctctgaagactacagcgtcgcca
gcgcagctctctctagcgacggccgcatcttcactggtgtcaatgtatatcattttactgggggaccttgtgcagaactcgtggtgctgggcactgctgctgctgcggcagctggca
acctgacttgtatcgtcgcgatcggaaatgagaacaggggcatcttgagcccctgcggacggtgccgacaggtgcttctcgatctgcatcctgggatcaaagccatagtgaag
gacagtgatggacagccgacggcagttgggattcgtgaattgctgccctctggttatgtgtgggagggctaagatcgatagatcctaatcaacctctggattacaaaatttgtgaa
agattgactggtattcttaactatgttgctccttttacgctatgtggatacgctgctttaatgcctttgtatcatgctattgcttcccgtatggctttcattttctcctccttgtataaatcctggttg
ctgtctctttatgaggagttgtggcccgttgtcaggcaacgtggcgtggtgtgcactgtgtttgctgacgcaacccccactggttggggcattgccaccacctgtcagctcctttccgg
gactttcgctttccccctccctattgccacggcggaactcatcgccgcctgccttgcccgctgctggacaggggctcggctgttgggcactgacaattccgtggtgttgtcggggaa
atcatcgtcctttccttggctgctcgcctgtgttgccacctggattctgcgcgggacgtccttctgctacgtcccttcggccctcaatccagcggaccttccttcccgcggcctgctgcc
ggctctgcggcctcttccgcgtcttcgccttcgccctcagacgagtcggatctccctttgggccgcctccccgcctggtacctttaagaccaatgacttacaaggcagctgtagatct
tagccactttttaaaagaaaaggggggactggaagggctaattcactcccaacgaagacaagatcacctgcaggacaggcgcgccctgctttttgcttgtactgggtctctctgg
ttagaccagatctgagcctgggagctctctggctaactagggaacccactgcttaagcctcaataaagcttgccttgagtgcttcaagtagtgtgtgcccgtctgttgtgtgactctg
gtaactagagatccctcagacccttttagtcagtgtggaaaatctctagcacccgggcgattaaggaaagggctagatcattcttgaagacgaaagggcctcgtgatacgcctat
ttttataggttaatgtcatgataataatggtttcttagacgtcaggtggcacttttcggggaaatgtgcgcggaacccctatttgtttatttttctaaatacattcaaatatgtatccgctcat
gagacaataaccctgataaatgcttcaataatattgaaaaaggaagagtatgagtattcaacatttccgtgtcgcccttattcccttttttgcggcattttgccttcctgtttttgctcaccc
agaaacgctggtgaaagtaaaagatgctgaagatcagttgggtgcacgagtgggttacatcgaactggatctcaacagcggtaagatccttgagagttttcgccccgaagaac
gttttccaatgatgagcacttttaaagttctgctatgtggcgcggtattatcccgtgttgacgccgggcaagagcaactcggtcgccgcatacactattctcagaatgacttggttgag
tactcaccagtcacagaaaagcatcttacggatggcatgacagtaagagaattatgcagtgctgccataaccatgagtgataacactgcggccaacttacttctgacaacgatc
ggaggaccgaaggagctaaccgcttttttgcacaacatgggggatcatgtaactcgccttgatcgttgggaaccggagctgaatgaagccataccaaacgacgagcgtgaca
ccacgatgcctgtagcaatggcaacaacgttgcgcaaactattaactggcgaactacttactctagcttcccggcaacaattaatagactggatggaggcggataaagttgcag
gaccacttctgcgctcggcccttccggctggctggtttattgctgataaatctggagccggtgagcgtgggtctcgcggtatcattgcagcactggggccagatggtaagccctcc
cgtatcgtagttatctacacgacggggagtcaggcaactatggatgaacgaaatagacagatcgctgagataggtgcctcactgattaagcattggtaactgtcagaccaagttt
actcatatatactttagattgatttaaaacttcatttttaatttaaaaggatctaggtgaagatcctttttgataatctcatgaccaaaatcccttaacgtgagttttcgttccactgagcgtc
agaccccgtagaaaagatcaaaggatcttcttgagatcctttttttctgcgcgtaatctgctgcttgcaaacaaaaaaaccaccgctaccagcggtggtttgtttgccggatcaaga
gctaccaactctttttccgaaggtaactggcttcagcagagcgcagataccaaatactgttcttctagtgtagccgtagttaggccaccacttcaagaactctgtagcaccgcctac
atacctcgctctgctaatcctgttaccagtggctgctgccagtggcgataagtcgtgtcttaccgggttggactcaagacgatagttaccggataaggcgcagcggtcgggctga
acggggggttcgtgcacacagcccagcttggagcgaacgacctacaccgaactgagatacctacagcgtgagctatgagaaagcgccacgcttcccgaagggagaaag
gcggacaggtatccggtaagcggcagggtcggaacaggagagcgcacgagggagcttccagggggaaacgcctggtatctttatagtcctgtcgggtttcgccacctctgac
ttgagcgtcgatttttgtgatgctcgtcaggggggcggagcctatggaaaaacgccagcaacgcggcctttttacggttcctggccttttgctggccttttgctcacatgttctttcctgc
gttatcccctgattctgtggataaccgtattaccgcctttgagtgagctgataccgctcgccgcagccgaacgaccgagcgcagcgagtcagtgagcgaggaagcggaagag
cgcccaatacgcaaaccgcctctccccgcgcgttggccgattcattaatgcagcaagctcatggctgactaattttttttatttatgcagaggccgaggccgcctcggcctctgagc
tattccagaagtagtgaggaggcttttttggaggcctaggcttttgcaaaaagctccccgtggcacgacaggtttcccgactggaaagcgggcagtgagcgcaacgcaattaat
gtgagttagctcactcattaggcaccccaggctttacactttatgcttccggctcgtatgttgtgtggaattgtgagcggataacaatttcacacaggaaacagctatgacatgattac
gaatttcacaaataaagcatttttttcactgcattctagttgtggtttgtccaaactcatcaatgtatcttatcatgtctggatcaactggataactcaagctaaccaaaatcatcccaaa
cttcccaccccatacccctattaccactgccaattacctgtggtttcatttactctaaacctgtgattcctctgaattattttcattttaaagaaattgtatttgttaaatatgtactacaaactta
gtagt

Name: pEF-1a_EGFP_IRES_BSD_WPRE (Addgene ID = 251221)

Map:

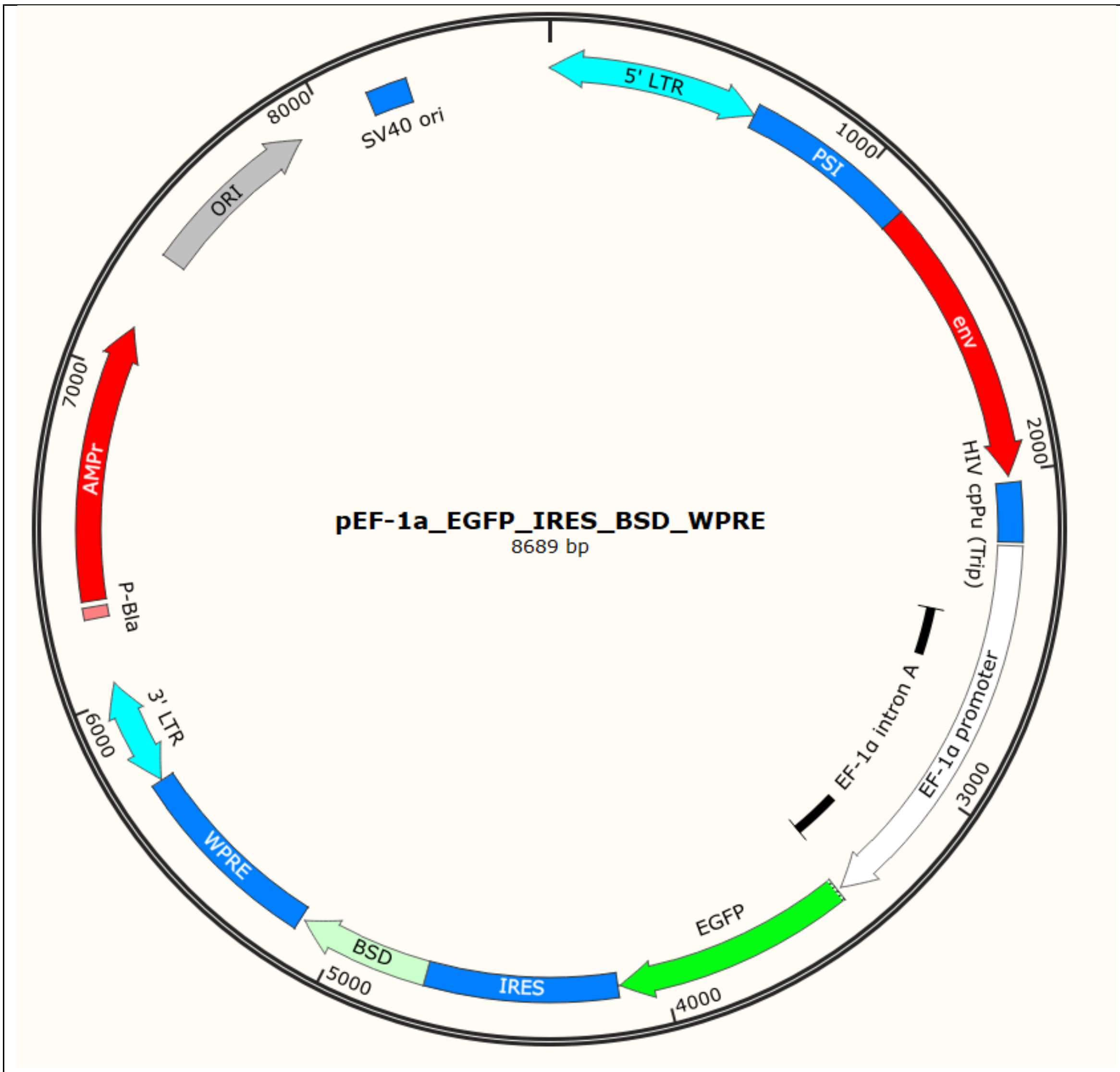

Sequence:

tggaagggctaattcactcccaaagaagacaagatatccttgatctgtggatctaccacacacaaggctacttccctgattagcagaactacacaccagggccaggggtcag
atatccactgacctttggatggtgctacaagctagtaccagttgagccagataaggtagaagaggccaataaaggagagaacaccagcttgttacaccctgtgagcctgcatg
ggatggatgacccggagagagaagtgttagagtggaggtttgacagccgcctagcatttcatcacgtggcccgagagctgcatccggagtacttcaagaactgctgatatcga
gcttgctacaagggactttccgctggggactttccagggaggcgtggcctgggcgggactggggagtggcgagccctcagatcctgcatataagcagctgctttttgcctgtact
gggtctctctggttagaccagatctgagcctgggagctctctggctaactagggaacccactgcttaagcctcaataaagcttgccttgagtgcttcaagtagtgtgtgcccgtctgt
tgtgtgactctggtaactagagatccctcagacccttttagtcagtgtggaaaatctctagcagtggcgcccgaacagggacttgaaagcgaaagggaaaccagaggagctct
ctcgacgcaggactcggcttgctgaagcgcgcacggcaagaggcgaggggcggcgactggtgagtacgccaaaaattttgactagcggaggctagaaggagagagatg
ggtgcgagagcgtcagtattaagcgggggagaattagatcgcgatgggaaaaaattcggttaaggccagggggaaagaaaaaatataaattaaaacatatagtatgggca
agcagggagctagaacgattcgcagttaatcctggcctgttagaaacatcagaaggctgtagacaaatactgggacagctacaaccatcccttcagacaggatcagaagaa
cttagatcattatataatacagtagcaaccctctattgtgtgcatcaaaggatagagataaaagacaccaaggaagctttagacaagatagaggaagagcaaaacaaaagta
agaccaccgcacagcaagcggccggccgctgatcttcagacctggaggaggagatatgagggacaattggagaagtgaattatataaatataaagtagtaaaaattgaac
cattaggagtagcacccaccaaggcaaagagaagagtggtgcagagagaaaaaagagcagtgggaataggagctttgttccttgggttcttgggagcagcaggaagcact
atgggcgcagcgtcaatgacgctgacggtacaggccagacaattattgtctggtatagtgcagcagcagaacaatttgctgagggctattgaggcgcaacagcatctgttgca
actcacagtctggggcatcaagcagctccaggcaagaatcctggctgtggaaagatacctaaaggatcaacagctcctggggatttggggttgctctggaaaactcatttgcac
cactgctgtgccttggaatgctagttggagtaataaatctctggaacagatttggaatcacacgacctggatggagtgggacagagaaattaacaattacacaagcttaatacac
tccttaattgaagaatcgcaaaaccagcaagaaaagaatgaacaagaattattggaattagataaatgggcaagtttgtggaattggtttaacataacaaattggctgtggtatat
aaaattattcataatgatagtaggaggcttggtaggtttaagaatagtttttgctgtactttctatagtgaatagagttaggcagggatattcaccattatcgtttcagacccacctccca

accccgaggggacccgacaggcccgaaggaatagaagaagaaggtggagagagagacagagacagatccattcgattagtgaacggatctcgacggtatcgccgaatt
cacaaatggcagtattcatccacaattttaaaagaaaagggggggattggggggtacagtgcaggggaaagaatagtagacataatagcaacagacatacaaactaaaga
attacaaaaacaaattacaaaaattcaaaattttcgggtttattacagggacagcagagatccagtttggactagtcgtgaggctccggtgcccgtcagtgggcagagcgcaca
tcgcccacagtccccgagaagttggggggaggggtcggcaattgaaccggtgcctagagaaggtggcgcggggtaaactgggaaagtgatgtcgtgtactggctccgccttt
ttcccgagggtgggggagaaccgtatataagtgcagtagtcgccgtgaacgttctttttcgcaacgggtttgccgccagaacacaggtaagtgccgtgtgtggttcccgcgggcc
tggcctctttacgggttatggcccttgcgtgccttgaattacttccacctggctgcagtacgtgattcttgatcccgagcttcgggttggaagtgggtgggagagttcgaggccttgcg
cttaaggagccccttcgcctcgtgcttgagttgaggcctggcctgggcgctggggccgccgcgtgcgaatctggtggcaccttcgcgcctgtctcgctgctttcgataagtctctag
ccatttaaaatttttgatgacctgctgcgacgctttttttctggcaagatagtcttgtaaatgcgggccaagatctgcacactggtatttcggtttttggggccgcgggcggcgacggg
gcccgtgcgtcccagcgcacatgttcggcgaggcggggcctgcgagcgcggccaccgagaatcggacgggggtagtctcaagctggccggcctgctctggtgcctggcctc
gcgccgccgtgtatcgccccgccctgggcggcaaggctggcccggtcggcaccagttgcgtgagcggaaagatggccgcttcccggccctgctgcagggagctcaaaatg
gaggacgcggcgctcgggagagcgggcgggtgagtcacccacacaaaggaaaagggcctttccgtcctcagccgtcgcttcatgtgactccacggagtaccgggcgccgt
ccaggcacctcgattagttctcgagcttttggagtacgtcgtctttaggttggggggaggggttttatgcgatggagtttccccacactgagtgggtggagactgaagttaggccag
cttggcacttgatgtaattctccttggaatttgccctttttgagtttggatcttggttcattctcaagcctcagacagtggttcaaagtttttttcttccatttcaggtgtcgtgaagcggccgcc
accatggtgagcaagggcgaggagctgttcaccggggtggtgcccatcctggtcgagctggacggcgacgtaaacggccacaagttcagcgtgtccggcgagggcgagg
gcgatgccacctacggcaagctgaccctgaagttcatctgcaccaccggcaagctgcccgtgccctggcccaccctcgtgaccaccctgacctacggcgtgcagtgcttcagc
cgctaccccgaccacatgaagcagcacgacttcttcaagtccgccatgcccgaaggctacgtccaggagcgcaccatcttcttcaaggacgacggcaactacaagacccgc
gccgaggtgaagttcgagggcgacaccctggtgaaccgcatcgagctgaagggcatcgacttcaaggaggacggcaacatcctggggcacaagctggagtacaactaca
acagccacaacgtctatatcatggccgacaagcagaagaacggcatcaaggtgaacttcaagatccgccacaacatcgaggacggcagcgtgcagctcgccgaccacta
ccagcagaacacccccatcggcgacggccccgtgctgctgcccgacaaccactacctgagcacccagtccgccctgagcaaagaccccaacgagaagcgcgatcacat
ggtcctgctggagttcgtgaccgccgccgggatcactctcggcatggacgagctgtacaagtaaggatccctcccccccccctaacgttactggccgaagccgcttggaataa
ggccggtgtgcgtttgtctatatgttattttccaccatattgccgtcttttggcaatgtgagggcccggaaacctggccctgtcttcttgacgagcattcctaggggtctttcccctctcgc
caaaggaatgcaaggtctgttgaatgtcgtgaaggaagcagttcctctggaagcttcttgaagacaaacaacgtctgtagcgaccctttgcaggcagcggaaccccccacctg
gcgacaggtgcctctgcggccaaaagccacgtgtataagatacacctgcaaaggcggcacaaccccagtgccacgttgtgagttggatagttgtggaaagagtcaaatggct
ctcctcaagcgtattcaacaaggggctgaaggatgcccagaaggtaccccattgtatgggatctgatctggggcctcggtgcacatgctttacatgtgtttagtcgaggttaaaaa
aacgtctaggccccccgaaccacggggacgtggttttcctttgaaaaacacgatgataatatggccacacatatggccaagcctttgtctcaagaagaatccaccctcattgaa
agagcaacggctacaatcaacagcatccccatctctgaagactacagcgtcgccagcgcagctctctctagcgacggccgcatcttcactggtgtcaatgtatatcattttactgg
gggaccttgtgcagaactcgtggtgctgggcactgctgctgctgcggcagctggcaacctgacttgtatcgtcgcgatcggaaatgagaacaggggcatcttgagcccctgcg
gacggtgccgacaggtgcttctcgatctgcatcctgggatcaaagccatagtgaaggacagtgatggacagccgacggcagttgggattcgtgaattgctgccctctggttatgt
gtgggagggctaaatcgatagatcctaatcaacctctggattacaaaatttgtgaaagattgactggtattcttaactatgttgctccttttacgctatgtggatacgctgctttaatgcct
ttgtatcatgctattgcttcccgtatggctttcattttctcctccttgtataaatcctggttgctgtctctttatgaggagttgtggcccgttgtcaggcaacgtggcgtggtgtgcactgtgtttg
ctgacgcaacccccactggttggggcattgccaccacctgtcagctcctttccgggactttcgctttccccctccctattgccacggcggaactcatcgccgcctgccttgcccgct
gctggacaggggctcggctgttgggcactgacaattccgtggtgttgtcggggaaatcatcgtcctttccttggctgctcgcctgtgttgccacctggattctgcgcgggacgtccttc
tgctacgtcccttcggccctcaatccagcggaccttccttcccgcggcctgctgccggctctgcggcctcttccgcgtcttcgccttcgccctcagacgagtcggatctccctttgggc
cgcctccccgcctggtacctttaagaccaatgacttacaaggcagctgtagatcttagccactttttaaaagaaaaggggggactggaagggctaattcactcccaacgaaga
caagatcacctgcaggacaggcgcgccctgctttttgcttgtactgggtctctctggttagaccagatctgagcctgggagctctctggctaactagggaacccactgcttaagcct
caataaagcttgccttgagtgcttcaagtagtgtgtgcccgtctgttgtgtgactctggtaactagagatccctcagacccttttagtcagtgtggaaaatctctagcacccgggcgat
taaggaaagggctagatcattcttgaagacgaaagggcctcgtgatacgcctatttttataggttaatgtcatgataataatggtttcttagacgtcaggtggcacttttcggggaaat
gtgcgcggaacccctatttgtttatttttctaaatacattcaaatatgtatccgctcatgagacaataaccctgataaatgcttcaataatattgaaaaaggaagagtatgagtattca
acatttccgtgtcgcccttattcccttttttgcggcattttgccttcctgtttttgctcacccagaaacgctggtgaaagtaaaagatgctgaagatcagttgggtgcacgagtgggttac
atcgaactggatctcaacagcggtaagatccttgagagttttcgccccgaagaacgttttccaatgatgagcacttttaaagttctgctatgtggcgcggtattatcccgtgttgacgc
cgggcaagagcaactcggtcgccgcatacactattctcagaatgacttggttgagtactcaccagtcacagaaaagcatcttacggatggcatgacagtaagagaattatgca
gtgctgccataaccatgagtgataacactgcggccaacttacttctgacaacgatcggaggaccgaaggagctaaccgcttttttgcacaacatgggggatcatgtaactcgcc
ttgatcgttgggaaccggagctgaatgaagccataccaaacgacgagcgtgacaccacgatgcctgtagcaatggcaacaacgttgcgcaaactattaactggcgaactact
tactctagcttcccggcaacaattaatagactggatggaggcggataaagttgcaggaccacttctgcgctcggcccttccggctggctggtttattgctgataaatctggagccg
gtgagcgtgggtctcgcggtatcattgcagcactggggccagatggtaagccctcccgtatcgtagttatctacacgacggggagtcaggcaactatggatgaacgaaataga
cagatcgctgagataggtgcctcactgattaagcattggtaactgtcagaccaagtttactcatatatactttagattgatttaaaacttcatttttaatttaaaaggatctaggtgaaga
tcctttttgataatctcatgaccaaaatcccttaacgtgagttttcgttccactgagcgtcagaccccgtagaaaagatcaaaggatcttcttgagatcctttttttctgcgcgtaatctgc
tgcttgcaaacaaaaaaaccaccgctaccagcggtggtttgtttgtcggatcaagagctaccaactctttttccgaaggtaactggcttcagcagagcgcagataccaaatactg
ttcttctagtgtagccgtagttaggccaccacttcaagaactctgtagcaccgcctacatacctcgctctgctaatcctgttaccagtggctgctgccagtggcgataagtcgtgtctt
accgggttggactcaagacgatagttaccggataaggcgcagcggtcgggctgaacggggggttcgtgcacacagcccagcttggagcgaacgacctacaccgaactga

gatacctacagcgtgagctatgagaaagcgccacgcttcccgaagggagaaaggcggacaggtatccggtaagcggcagggtcggaacaggagagcgcacgaggga
gcttccagggggaaacgcctggtatctttatagtcctgtcgggtttcgccacctctgacttgagcgtcgatttttgtgatgctcgtcaggggggcggagcctatggaaaaacgccag
caacgcggcctttttacggttcctggccttttgctggccttttgctcacatgttctttcctgcgttatcccctgattctgtggataaccgtattaccgcctttgagtgagctgataccgctcgc
cgcagccgaacgaccgagcgcagcgagtcagtgagcgaggaagcggaagagcgcccaatacgcaaaccgcctctccccgcgcgttggccgattcattaatgcagcaag
ctcatggctgactaattttttttatttatgcagaggccgaggccgcctcggcctctgagctattccagaagtagtgaggaggcttttttggaggcctaggcttttgcaaaaagctcccc
gtggcacgacaggtttcccgactggaaagcgggcagtgagcgcaacgcaattaatgtgagttagctcactcattaggcaccccaggctttacactttatgcttccggctcgtatgt
tgtgtggaattgtgagcggataacaatttcacacaggaaacagctatgacatgattacgaatttcacaaataaagcatttttttcactgcattctagttgtggtttgtccaaactcatca
atgtatcttatcatgtctggatcaactggataactcaagctaaccaaaatcatcccaaacttcccaccccataccctattaccactgccaattacctgtggtttcatttactctaaacct
gtgattcctctgaattattttcattttaaagaaattgtatttgttaaatatgtactacaaacttagtagt

Name: pEF-1a_mScarlet_IRES_BSD_WPRE (Addgene ID = 251222)

Map:

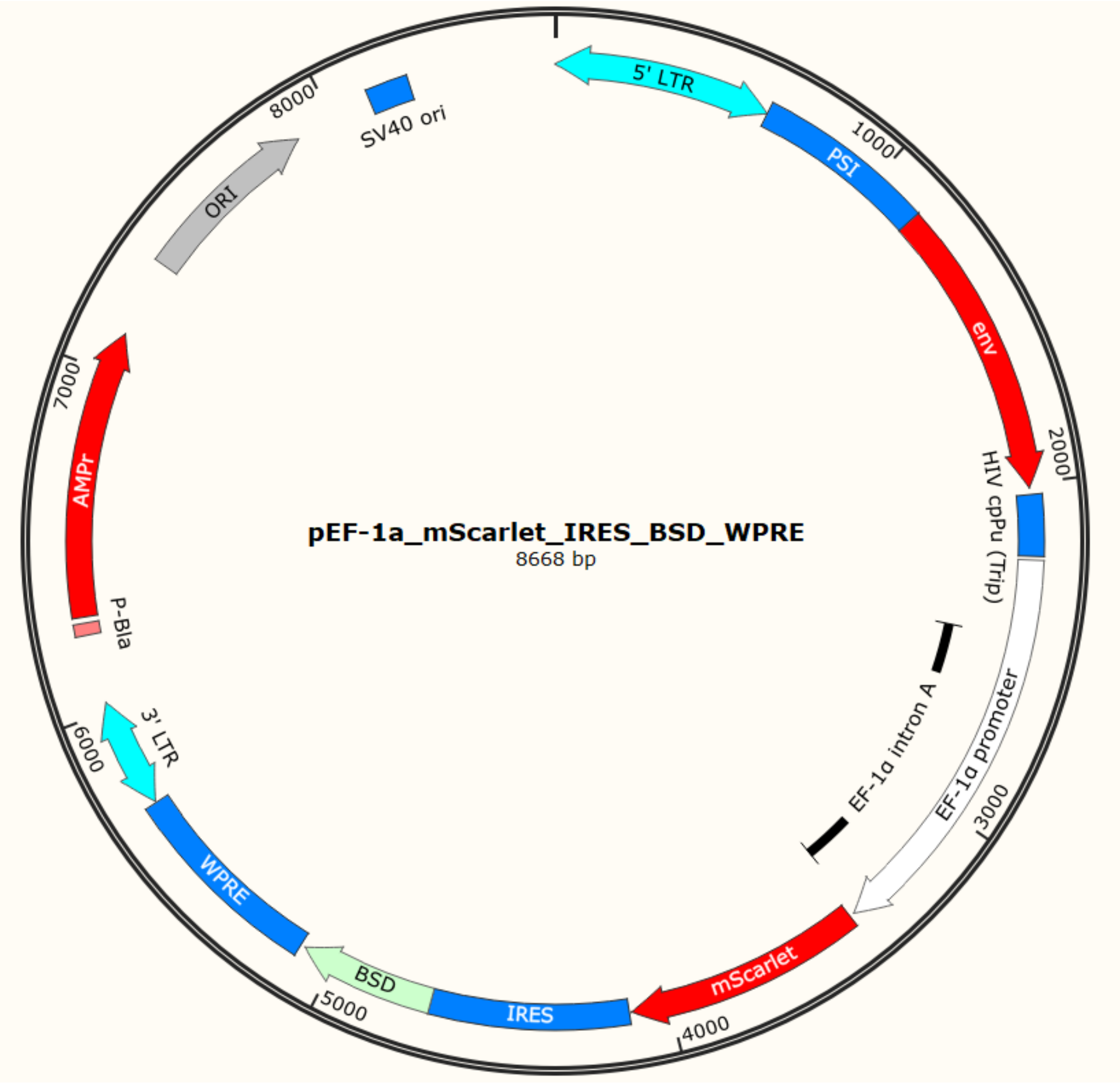

Sequence:

tggaagggctaattcactcccaaagaagacaagatatccttgatctgtggatctaccacacacaaggctacttccctgattagcagaactacacaccagggccaggggtcag
atatccactgacctttggatggtgctacaagctagtaccagttgagccagataaggtagaagaggccaataaaggagagaacaccagcttgttacaccctgtgagcctgcatg
ggatggatgacccggagagagaagtgttagagtggaggtttgacagccgcctagcatttcatcacgtggcccgagagctgcatccggagtacttcaagaactgctgatatcga
gcttgctacaagggactttccgctggggactttccagggaggcgtggcctgggcgggactggggagtggcgagccctcagatcctgcatataagcagctgctttttgcctgtact

gggtctctctggttagaccagatctgagcctgggagctctctggctaactagggaacccactgcttaagcctcaataaagcttgccttgagtgcttcaagtagtgtgtgcccgtctgt
tgtgtgactctggtaactagagatccctcagacccttttagtcagtgtggaaaatctctagcagtggcgcccgaacagggacttgaaagcgaaagggaaaccagaggagctct
ctcgacgcaggactcggcttgctgaagcgcgcacggcaagaggcgaggggcggcgactggtgagtacgccaaaaattttgactagcggaggctagaaggagagagatg
ggtgcgagagcgtcagtattaagcgggggagaattagatcgcgatgggaaaaaattcggttaaggccagggggaaagaaaaaatataaattaaaacatatagtatgggca
agcagggagctagaacgattcgcagttaatcctggcctgttagaaacatcagaaggctgtagacaaatactgggacagctacaaccatcccttcagacaggatcagaagaa
cttagatcattatataatacagtagcaaccctctattgtgtgcatcaaaggatagagataaaagacaccaaggaagctttagacaagatagaggaagagcaaaacaaaagta
agaccaccgcacagcaagcggccggccgctgatcttcagacctggaggaggagatatgagggacaattggagaagtgaattatataaatataaagtagtaaaaattgaac
cattaggagtagcacccaccaaggcaaagagaagagtggtgcagagagaaaaaagagcagtgggaataggagctttgttccttgggttcttgggagcagcaggaagcact
atgggcgcagcgtcaatgacgctgacggtacaggccagacaattattgtctggtatagtgcagcagcagaacaatttgctgagggctattgaggcgcaacagcatctgttgca
actcacagtctggggcatcaagcagctccaggcaagaatcctggctgtggaaagatacctaaaggatcaacagctcctggggatttggggttgctctggaaaactcatttgcac
cactgctgtgccttggaatgctagttggagtaataaatctctggaacagatttggaatcacacgacctggatggagtgggacagagaaattaacaattacacaagcttaatacac
tccttaattgaagaatcgcaaaaccagcaagaaaagaatgaacaagaattattggaattagataaatgggcaagtttgtggaattggtttaacataacaaattggctgtggtatat
aaaattattcataatgatagtaggaggcttggtaggtttaagaatagtttttgctgtactttctatagtgaatagagttaggcagggatattcaccattatcgtttcagacccacctccca
accccgaggggacccgacaggcccgaaggaatagaagaagaaggtggagagagagacagagacagatccattcgattagtgaacggatctcgacggtatcgccgaatt
cacaaatggcagtattcatccacaattttaaaagaaaaggggggattggggggtacagtgcaggggaaagaatagtagacataatagcaacagacatacaaactaaaga
attacaaaaacaaattacaaaaattcaaaattttcgggtttattacagggacagcagagatccagtttggactagtcgtgaggctccggtgcccgtcagtgggcagagcgcaca
tcgcccacagtccccgagaagttggggggaggggtcggcaattgaaccggtgcctagagaaggtggcgcggggtaaactgggaaagtgatgtcgtgtactggctccgccttt
ttcccgagggtgggggagaaccgtatataagtgcagtagtcgccgtgaacgttctttttcgcaacgggtttgccgccagaacacaggtaagtgccgtgtgtggttcccgcgggcc
tggcctctttacgggttatggcccttgcgtgccttgaattacttccacctggctgcagtacgtgattcttgatcccgagcttcgggttggaagtgggtgggagagttcgaggccttgcg
cttaaggagccccttcgcctcgtgcttgagttgaggcctggcctgggcgctggggccgccgcgtgcgaatctggtggcaccttcgcgcctgtctcgctgctttcgataagtctctag
ccatttaaaatttttgatgacctgctgcgacgctttttttctggcaagatagtcttgtaaatgcgggccaagatctgcacactggtatttcggtttttggggccgcgggcggcgacggg
gcccgtgcgtcccagcgcacatgttcggcgaggcggggcctgcgagcgcggccaccgagaatcggacgggggtagtctcaagctggccggcctgctctggtgcctggcctc
gcgccgccgtgtatcgccccgccctgggcggcaaggctggcccggtcggcaccagttgcgtgagcggaaagatggccgcttcccggccctgctgcagggagctcaaaatg
gaggacgcggcgctcgggagagcgggcgggtgagtcacccacacaaaggaaaagggcctttccgtcctcagccgtcgcttcatgtgactccacggagtaccgggcgccgt
ccaggcacctcgattagttctcgagcttttggagtacgtcgtctttaggttggggggaggggttttatgcgatggagtttccccacactgagtgggtggagactgaagttaggccag
cttggcacttgatgtaattctccttggaatttgccctttttgagtttggatcttggttcattctcaagcctcagacagtggttcaaagtttttttcttccatttcaggtgtcgtgaagcggccgcc
accatggtgagcaagggcgaggcagtgatcaaggagttcatgcggttcaaggtgcacatggagggctccatgaacggccacgagttcgagatcgagggcgagggcgagg
gccgcccctacgagggcacccagaccgccaagctgaaggtgaccaagggtggccccctgcccttctcctgggacatcctgtcccctcagttcatgtacggctccagggccttc
accaagcaccccgccgacatccccgactactataagcagtccttccccgagggcttcaagtgggagcgcgtgatgaacttcgaggacggcggcgccgtgaccgtgaccca
ggacacctccctggaggacggcaccctgatctacaaggtgaagctccgcggcaccaacttccctcctgacggccccgtaatgcagaagaagacaatgggctgggaagcgt
ccaccgagcggttgtaccccgaggacggcgtgctgaagggcgacattaagatggccctgcgcctgaaggacggcggccgctacctggcggacttcaagaccacctacaa
ggccaagaagcccgtgcagatgcccggcgcctacaacgtcgaccgcaagttggacatcacctcccacaacgaggactacaccgtggtggaacagtacgaacgctccgag
ggccgccactccaccggcggcatggacgagctgtacaagtaaggatccctcccccccccctaacgttactggccgaagccgcttggaataaggccggtgtgcgtttgtctatat
gttattttccaccatattgccgtcttttggcaatgtgagggcccggaaacctggccctgtcttcttgacgagcattcctaggggtctttcccctctcgccaaaggaatgcaaggtctgtt
gaatgtcgtgaaggaagcagttcctctggaagcttcttgaagacaaacaacgtctgtagcgaccctttgcaggcagcggaaccccccacctggcgacaggtgcctctgcggc
caaaagccacgtgtataagatacacctgcaaaggcggcacaaccccagtgccacgttgtgagttggatagttgtggaaagagtcaaatggctctcctcaagcgtattcaacaa
ggggctgaaggatgcccagaaggtaccccattgtatgggatctgatctggggcctcggtgcacatgctttacatgtgtttagtcgaggttaaaaaaacgtctaggccccccgaac
cacggggacgtggttttcctttgaaaaacacgatgataatatggccacacatatggccaagcctttgtctcaagaagaatccaccctcattgaaagagcaacggctacaatcaa
cagcatccccatctctgaagactacagcgtcgccagcgcagctctctctagcgacggccgcatcttcactggtgtcaatgtatatcattttactggggggaccttgtgcagaactcgt
ggtgctgggcactgctgctgctgcggcagctggcaacctgacttgtatcgtcgcgatcggaaatgagaacaggggcatcttgagcccctgcggacggtgccgacaggtgcttct
cgatctgcatcctgggatcaaagccatagtgaaggacagtgatggacagccgacggcagttgggattcgtgaattgctgccctctggttatgtgtgggagggctaaatcgatag
atcctaatcaacctctggattacaaaatttgtgaaagattgactggtattcttaactatgttgctccttttacgctatgtggatacgctgctttaatgcctttgtatcatgctattgcttcccgta
tggctttcattttctcctccttgtataaatcctggttgctgtctctttatgaggagttgtggcccgttgtcaggcaacgtggcgtggtgtgcactgtgtttgctgacgcaacccccactggtt
ggggcattgccaccacctgtcagctcctttccgggactttcgctttccccctccctattgccacggcggaactcatcgccgcctgccttgcccgctgctggacaggggctcggctgtt
gggcactgacaattccgtggtgttgtcggggaaatcatcgtcctttccttggctgctcgcctgtgttgccacctggattctgcgcgggacgtccttctgctacgtcccttcggccctcaa
tccagcggaccttccttcccgcggcctgctgccggctctgcggcctcttccgcgtcttcgccttcgccctcagacgagtcggatctccctttgggccgcctccccgcctggtaccttta
agaccaatgacttacaaggcagctgtagatcttagccactttttaaaagaaaaggggggactggaagggctaattcactcccaacgaagacaagatcacctgcaggacagg
cgcgccctgctttttgcttgtactgggtctctctggttagaccagatctgagcctgggagctctctggctaactagggaacccactgcttaagcctcaataaagcttgccttgagtgctt
caagtagtgtgtgcccgtctgttgtgtgactctggtaactagagatccctcagacccttttagtcagtgtggaaaatctctagcacccgggcgattaaggaaagggctagatcattct
tgaagacgaaagggcctcgtgatacgcctatttttataggttaatgtcatgataataatggtttcttagacgtcaggtggcacttttcggggaaatgtgcgcggaacccctatttgttta

tttttctaaatacattcaaatatgtatccgctcatgagacaataaccctgataaatgcttcaataatattgaaaaaggaagagtatgagtattcaacatttccgtgtcgcccttattccct
tttttgcggcattttgccttcctgtttttgctcacccagaaacgctggtgaaagtaaaagatgctgaagatcagttgggtgcacgagtgggttacatcgaactggatctcaacagcgg
taagatccttgagagttttcgccccgaagaacgttttccaatgatgagcacttttaaagttctgctatgtggcgcggtattatcccgtgttgacgccgggcaagagcaactcggtcgc
cgcatacactattctcagaatgacttggttgagtactcaccagtcacagaaaagcatcttacggatggcatgacagtaagagaattatgcagtgctgccataaccatgagtgata
acactgcggccaacttacttctgacaacgatcggaggaccgaaggagctaaccgcttttttgcacaacatgggggatcatgtaactcgccttgatcgttgggaaccggagctga
atgaagccataccaaacgacgagcgtgacaccacgatgcctgtagcaatggcaacaacgttgcgcaaactattaactggcgaactacttactctagcttcccggcaacaatta
atagactggatggaggcggataaagttgcaggaccacttctgcgctcggcccttccggctggctggtttattgctgataaatctggagccggtgagcgtgggtctcgcggtatcatt
gcagcactggggccagatggtaagccctcccgtatcgtagttatctacacgacggggagtcaggcaactatggatgaacgaaatagacagatcgctgagataggtgcctcac
tgattaagcattggtaactgtcagaccaagtttactcatatatactttagattgatttaaaacttcatttttaatttaaaaggatctaggtgaagatcctttttgataatctcatgaccaaaat
cccttaacgtgagttttcgttccactgagcgtcagaccccgtagaaaagatcaaaggatcttcttgagatcctttttttctgcgcgtaatctgctgcttgcaaacaaaaaaaccaccg
ctaccagcggtggtttgtttgtcggatcaagagctaccaactctttttccgaaggtaactggcttcagcagagcgcagataccaaatactgttcttctagtgtagccgtagttaggcc
accacttcaagaactctgtagcaccgcctacatacctcgctctgctaatcctgttaccagtggctgctgccagtggcgataagtcgtgtcttaccgggttggactcaagacgatagt
taccggataaggcgcagcggtcgggctgaacggggggttcgtgcacacagcccagcttggagcgaacgacctacaccgaactgagatacctacagcgtgagctatgaga
aagcgccacgcttcccgaagggagaaaggcggacaggtatccggtaagcggcagggtcggaacaggagagcgcacgagggagcttccagggggaaacgcctggtatc
tttatagtcctgtcgggtttcgccacctctgacttgagcgtcgatttttgtgatgctcgtcaggggggcggagcctatggaaaaacgccagcaacgcggcctttttacggttcctggcc
ttttgctggccttttgctcacatgttctttcctgcgttatcccctgattctgtggataaccgtattaccgcctttgagtgagctgataccgctcgccgcagccgaacgaccgagcgcagc
gagtcagtgagcgaggaagcggaagagcgcccaatacgcaaaccgcctctccccgcgcgttggccgattcattaatgcagcaagctcatggctgactaattttttttatttatgc
agaggccgaggccgcctcggcctctgagctattccagaagtagtgaggaggcttttttggaggcctaggcttttgcaaaaagctccccgtggcacgacaggtttcccgactgga
aagcgggcagtgagcgcaacgcaattaatgtgagttagctcactcattaggcaccccaggctttacactttatgcttccggctcgtatgttgtgtggaattgtgagcggataacaat
ttcacacaggaaacagctatgacatgattacgaatttcacaaataaagcatttttttcactgcattctagttgtggtttgtccaaactcatcaatgtatcttatcatgtctggatcaactgg
ataactcaagctaaccaaaatcatcccaaacttcccaccccataccctattaccactgccaattacctgtggtttcatttactctaaacctgtgattcctctgaattattttcattttaaag
aaattgtatttgttaaatatgtactacaaacttagtagt

Supplementary References

1. Cao, X. *et al.* Tissue microenvironment dictates the state of human induced pluripotent stem cell-derived endothelial cells of distinct developmental origin in 3D cardiac microtissues. Preprint at https://doi.org/10.1101/2022.11.22.517426 (2022).

2. Zhao, Z., Shang, P., Sage, F. & Geijsen, N. Ligation-assisted homologous recombination enables precise genome editing by deploying both MMEJ and HDR. *Nucleic Acids Res* **50**, e62–e62 (2022).

3. Love, M. I., Huber, W. & Anders, S. Moderated estimation of fold change and dispersion for RNA-seq data with DESeq2. *Genome Biol* **15**, (2014).

4. Ge, S. X., Jung, D., Jung, D. & Yao, R. ShinyGO: A graphical gene-set enrichment tool for animals and plants. *Bioinformatics* **36**, 2628–2629 (2020).

5. Szklarczyk, D. *et al.* The STRING database in 2023: protein-protein association networks and functional enrichment analyses for any sequenced genome of interest. *Nucleic Acids Res* **51**, D638–D646 (2023).

6. Shi, P. *et al.* Collateral activity of the CRISPR/RfxCas13d system in human cells. *Commun Biol* **6**, (2023).

7. Li, Y. *et al.* The collateral activity of RfxCas13d can induce lethality in a RfxCas13d knock-in mouse model. *Genome Biol* **24**, 1–25 (2023).

8. Brogan, D. J. *et al.* Development of a Rapid and Sensitive CasRx-Based Diagnostic Assay for SARS-CoV-2. *ACS Sens* **6**, 3957–3966 (2021).

9. Li, L. *et al.* A field-deployable method for single and multiplex detection of DNA or RNA from pathogens using Cas12 and Cas13. *Sci China Life Sci* **65**, 1456–1465 (2022).

10. East-Seletsky, A. *et al.* RNA Targeting by Functionally Orthogonal Type VI-A CRISPR-Cas Enzymes. *Mol Cell* **66**, 373–383 (2017).

11. Tieu, V. *et al.* A versatile CRISPR-Cas13d platform for multiplexed transcriptomic regulation and metabolic engineering in primary human T cells. *Cell* **187**, 1278–1295 (2024).

12. Wessels, H. H. *et al.* Prediction of on-target and off-target activity of CRISPR–Cas13d guide RNAs using deep learning. *Nat Biotechnol* **42**, 628–637 (2024).
