# Supplementary material for: Temporal dynamics of collateral RNA cleavage by LbuCas13a in human cells
Source: Commun Biol. 2026 Jan 19;9:233. doi: 10.1038/s42003-026-09511-3 (PMC12901986; doi:10.1038/s42003-026-09511-3)
Supplement: Supplementary file 3 — Description of Additional Supplementary Files [file 42003_2026_9511_MOESM3_ESM.pdf]

Description of Additional Supplementary Files:

File name: Supplementary Data 1

Description: The source data behind the graphs in the paper.

File name: Supplementary Data 2

Description: The source data behind the graphs in the Supplementary Figures

File name: Supplementary Movie 1

Description: Representative example of the live imaging of HAP1 cells after iTOP with LbuCas13a and a dEGFP targeting guide RNA. These HAP1 cells do not express the target RNA, and thus function as the no target control. Annexin V staining is shown in red, CellTox Green staining is shown in green. Quantification is provided in Figure 5b.

File name: Supplementary Movie 2

Description: Representative example of the live imaging of HAP1-dEGFP cells after iTOP with LbuCas13a and a dEGFP targeting guide RNA. Annexin V staining is shown in red, CellTox Green staining is shown in green. Quantification is provided in Figure 5b.
