# Supplementary material for: Temporal dynamics of collateral RNA cleavage by LbuCas13a in human cells
Source: Commun Biol. 2026 Jan 19;9:233. doi: 10.1038/s42003-026-09511-3 (PMC12901986; doi:10.1038/s42003-026-09511-3)
Supplement: Supplementary file 8 — Reporting Summary [file 42003_2026_9511_MOESM8_ESM.pdf]

nature portfolio

Corresponding author(s): P. Shang, N. Geijsen

Last updated by author(s): Dec 17, 2025

Reporting Summary

Nature Portfolio wishes to improve the reproducibility of the work that we publish. This form provides structure for consistency and transparency in reporting. For further information on Nature Portfolio policies, see our Editorial Policies and the Editorial Policy Checklist.

Statistics

For all statistical analyses, confirm that the following items are present in the figure legend, table legend, main text, or Methods section.

n/a	Confirmed	
☐	☒	The exact sample size (*n*) for each experimental group/condition, given as a discrete number and unit of measurement
☐	☒	A statement on whether measurements were taken from distinct samples or whether the same sample was measured repeatedly
☐	☒	The statistical test(s) used AND whether they are one- or two-sided<br>*Only common tests should be described solely by name; describe more complex techniques in the Methods section.*
☐	☒	A description of all covariates tested
☐	☒	A description of any assumptions or corrections, such as tests of normality and adjustment for multiple comparisons
☐	☒	A full description of the statistical parameters including central tendency (e.g. means) or other basic estimates (e.g. regression coefficient) AND variation (e.g. standard deviation) or associated estimates of uncertainty (e.g. confidence intervals)
☐	☒	For null hypothesis testing, the test statistic (e.g. *F*, *t*, *r*) with confidence intervals, effect sizes, degrees of freedom and *P* value noted<br>*Give P values as exact values whenever suitable.*
☒	☐	For Bayesian analysis, information on the choice of priors and Markov chain Monte Carlo settings
☒	☐	For hierarchical and complex designs, identification of the appropriate level for tests and full reporting of outcomes
☒	☐	Estimates of effect sizes (e.g. Cohen's *d*, Pearson's *r*), indicating how they were calculated

Our web collection on statistics for biologists contains articles on many of the points above.

Software and code

Policy information about availability of computer code

Data collection	CellProfiler 4.2.8 was used to quantify the number of AnnexinV and CellToxGreen positive cells from the live imaging experiment.
Data analysis	Scripts for the RNA-seq analysis, total RNA-seq analysis and Nanopore sequencing coverage calculation and analysis and are available on GitHub (https://github.com/Geijsenlab/cas13). RNA-seq and total RNA-seq reads were trimmed with TrimGalore version 0.6.6, mapped with STAR version 2.7.11b and generated a count table from mapped reads with FeatureCounts 2.0.3. Differential gene expression analysis was performed with DESeq2 version 1.48.1. Functional enrichment analysis was performed with ShinyGo version 0.82 or on string-db.org version 12.0. Nanopore reads with trimmed with PoreChop (https://github.com/rrwick/Porechop), and mapped to the transcriptome with minimap2 2.21-r1071.

For manuscripts utilizing custom algorithms or software that are central to the research but not yet described in published literature, software must be made available to editors and reviewers. We strongly encourage code deposition in a community repository (e.g. GitHub). See the Nature Portfolio guidelines for submitting code & software for further information.

Data

Policy information about availability of data

All manuscripts must include a data availability statement. This statement should provide the following information, where applicable:

- Accession codes, unique identifiers, or web links for publicly available datasets
- A description of any restrictions on data availability
- For clinical datasets or third party data, please ensure that the statement adheres to our policy

The RNA-seq data has been deposited in the NCBI Gene Expression Omnibus (GSE220759). The total RNA-seq data with ERCC spike-in RNAs has been deposited at the NCBI Gene Expression Omnibus (GSE308909). The Nanopore sequencing data has been deposited at the NCBI Sequence Read Archive (PRJNA912090). All other source data is available in Supplementary Data 1 (main figures) and 2 (supplementary figures).

Research involving human participants, their data, or biological material

Policy information about studies with human participants or human data. See also policy information about sex, gender (identity/presentation), and sexual orientation and race, ethnicity and racism.

Reporting on sex and gender	No experiments involving human subjects were conducted
Reporting on race, ethnicity, or other socially relevant groupings	No experiments involving human subjects were conducted
Population characteristics	No experiments involving human subjects were conducted
Recruitment	No experiments involving human subjects were conducted
Ethics oversight	NA

Note that full information on the approval of the study protocol must also be provided in the manuscript.

Field-specific reporting

Please select the one below that is the best fit for your research. If you are not sure, read the appropriate sections before making your selection.

☒ Life sciences ☐ Behavioural & social sciences ☐ Ecological, evolutionary & environmental sciences

For a reference copy of the document with all sections, see nature.com/documents/nr-reporting-summary-flat.pdf

Life sciences study design

All studies must disclose on these points even when the disclosure is negative.

Sample size	We used three biological replicates unless otherwise noted in the figure legends. No predetermined sample-size calculation was performed.
Data exclusions	Data were excluded only in cases of clearly defined technical failure, established quality-control metrics not being met, or pre-existing sample abnormalities that precluded meaningful comparison.<br>Nanopore replicate 1, sample 0 minutes after iTOP, dEGFP targeting condition, was excluded due to failed library prep and consequently too few reads for analysis.<br>Total RNA-seq with ERCC spike-in RNA: Replicate 3 of the empty iTOP condition at the 50 minutes after iTOP timepoint was excluded from analysis because it failed library prep QC (too low concentration), had a high percentage of rRNA mapped reads, and low correlation between ERCC spike-in RNA counts and their concentrations.<br>In figure 2, one replicate of 18S rRNA targeting in U2OS (due to unstable baseline fluorescence, but collateral cleavage fragment were visible as expected), one replicate of GAPDH targeting in U2OS (extremely low signal), and one replicate of the non-targeting treatment in ARPE19 (no signal past 18S rRNA peak, but no degradation visible in the 18S rRNA peak and preceding area as expected) were excluded due to technical bioanalyzer artifacts.<br>In the live imaging experiment in figure 3b, one replicate in HAP1 of the empty iTOP (CTRL), SP1 and SP2 treatments was excluded due to having a 2-3x higher starting number of annexin V and celltox green cells than all other samples.
Replication	Replicates refer to independent biological replicates. All results were reproducible.
Randomization	For all cell culture experiments, cells in each condition were splits taken from the same dish.
Blinding	Authors were not blinded.

Reporting for specific materials, systems and methods

We require information from authors about some types of materials, experimental systems and methods used in many studies. Here, indicate whether each material, system or method listed is relevant to your study. If you are not sure if a list item applies to your research, read the appropriate section before selecting a response.

Materials & experimental systems

n/a	Involved in the study
☐	☒ Antibodies
☐	☒ Eukaryotic cell lines
☒	☐ Palaeontology and archaeology
☒	☐ Animals and other organisms
☒	☐ Clinical data
☒	☐ Dual use research of concern
☒	☐ Plants

Methods

n/a	Involved in the study
☒	☐ ChIP-seq
☐	☒ Flow cytometry
☒	☐ MRI-based neuroimaging

Antibodies

Antibodies used	GAPDH loading control antibody (ThermoFisher, MA5-15738), anti-HA tag (ThermoFisher, 26183), Goat Anti-Mouse IgG H&L (HRP) (Abcam, ab6789)
Validation	GAPDH loading control antibody (ThermoFisher, MA5-15738): Thermofisher classifies it as "advanced verification", by knockdown.<br>anti-HA tag (ThermoFisher, 26183): Thermofisher classifies it as "advanced verification", by relative expression.

Eukaryotic cell lines

Policy information about cell lines and Sex and Gender in Research

Cell line source(s)	HAP1 cells were a gift from Dr. Brummelkamp, NKI Amsterdam. RH30 and RD cells were a gift from Dr. Gerben Schaaf, Erasmus MC. HEK293T cells were ordered from Takara (#632180). U2OS and ARPE19 cells were ordered from ATCC (HTB-96 and CRL-2302 respectively).
Authentication	No cell line authentication was performed
Mycoplasma contamination	All cell lines were tested for mycoplasma monthly.
Commonly misidentified lines (See ICLAC register)	NA

Plants

Seed stocks	NA
Novel plant genotypes	NA
Authentication	NA

Flow Cytometry

Plots

Confirm that:

☒ The axis labels state the marker and fluorochrome used (e.g. CD4-FITC).

☒ The axis scales are clearly visible. Include numbers along axes only for bottom left plot of group (a 'group' is an analysis of identical markers).

☒ All plots are contour plots with outliers or pseudocolor plots.

☒ A numerical value for number of cells or percentage (with statistics) is provided.

Methodology

Sample preparation	Cells were trypsonized, collected in culture medium, pelleted by centrifugation and resuspended in PBS + 5% FBS for analysis or culture medium for sorting.
Instrument	BD™ FACSCanto II, BD FACS Jazz, Cytoflex SRT
Software	FACSDiva, CytExpert SRT
Cell population abundance	Most experiments used FACS as an analyzer. HAP-GFP cells sorted for different GFP intensities were verified by qPCR. Cells sorted for expressing LbuCas13a-BFP after plasmid transfection were not assessed for purity post sort due to low positive cell numbers. The fluorescence of the RD-EGFP and RH30-mScarlet lines was confirmed on a bench top fluorescent microscope.
Gating strategy	Cells were first gated on a FSC-A vs SSC-A plot (P1). Then doublets were excluded on a FSC-Width vs FSC-H plot (P2), and by a SSC-Width vs SSC-H plot (P3). An example is shown in supplementary figure 18a. The percentage of fluorophore "A" or "B" positive cells in the P3 population was assessed by plotting the fluorphores against each other. Gates were set using fluorophore negative and single positive cells.

☒ Tick this box to confirm that a figure exemplifying the gating strategy is provided in the Supplementary Information.
